# Stochastic Navier–Stokes Equations on a Thin Spherical Domain

**DOI:** 10.1007/s00245-020-09702-2

**Published:** 2020-07-11

**Authors:** Zdzisław Brzeźniak, Gaurav Dhariwal, Quoc Thong Le Gia

**Affiliations:** 1 Department of Mathematics, University of York, Heslington, York, YO10 5DD UK; 2 Institute of Analysis and Scientific Computing, Vienna University of Technology, Vienna, Austria; 3 School of Mathematics and Statistics, University of New South Wales, Sydney, NSW 2052 Australia

**Keywords:** Stochastic Navier–Stokes equations, Navier–Stokes equations on a sphere, Singular limit, Primary 60H15, Secondary 35R60, 35Q30, 76D05

## Abstract

Incompressible Navier–Stokes equations on a thin spherical domain $Q_\varepsilon$ along with free boundary conditions under a random forcing are considered. The convergence of the martingale solution of these equations to the martingale solution of the stochastic Navier–Stokes equations on a sphere $\mathbb {S}^2$ as the thickness converges to zero is established.

## Introduction

For various motivations, partial differential equations in thin domains have been studied extensively in the last few decades; e.g. Babin and Vishik [4], Ciarlet [16], Ghidaglia and Temam [18], Marsden et al. [37] and references there in. The study of the Navier–Stokes equations (NSE) on thin domains originates in a series of papers by Hale and Raugel [20–22] concerning the reaction-diffusion and damped wave equations on thin domains. Raugel and Sell [44, 45] proved the global existence of strong solutions to NSE on thin domains for large initial data and forcing terms, in the case of purely periodic and periodic-Dirichlet boundary conditions. Later, by applying a contraction principle argument and carefully analysing the dependence of the solution on the first eigenvalue of the corresponding Laplace operator, Arvin [2] showed global existence of strong solutions of the Navier–Stokes equations on thin three-dimensional domains for large data. Temam and Ziane [52] generalised the results of [44, 45] to other boundary conditions. Moise et al. [41] proved global existence of strong solutions for initial data larger than in [45]. Iftimie [26] showed the existence and uniqueness of solutions for less regular initial data which was further improved by Iftimie and Raugel [27] by reducing the regularity and increasing the size of initial data and forcing.

In the context of thin spherical shells, large-scale atmospheric dynamics that play an important role in global climate models and weather prediction can be described by the 3-dimensional Navier–Stokes equations in a thin rotating spherical shell [34, 35]. Temam and Ziane in [53] gave the mathematical justification for the primitive equations of the atmosphere and the oceans which are known to be the fundamental equations of meteorology and oceanography [36, 43]. The atmosphere is a compressible fluid occupying a thin layer around the Earth and whose dynamics can be described by the 3D compressible Navier–Stokes equations in thin layers. In [53] it was assumed that the atmosphere is incompressible and hence a 3D incompressible NSE on thin spherical shells could be used as a mathematical model. They proved that the averages in the radial direction of the strong solutions (whose existence for physically relevant initial data was established in the same article) to the NSE on the thin spherical shells converge to the solution of the NSE on the sphere as the thickness converges to zero. In a recent paper Saito [46] studied the 3D Boussinesq equations in thin spherical domains and proved the convergence of the average of weak solutions of the 3D Boussinesq equations to a 2D problem. More recent work on incompressible viscous fluid flows in a thin spherical shell was carried out in [23–25].

For the deterministic NSE on the sphere, Il’in and Filatov [28–30] considered the existence and uniqueness of solutions while Temam and Wang [51] considered inertial forms of NSE on spheres. Brzeźniak et al. proved the existence and uniqueness of the solutions to the stochastic NSE on the rotating two dimensional sphere and also proved the existence of an asymptotically compact random dynamical system [9]. Recently, Brzeźniak et al. established [10] the existence of random attractors for the NSE on two dimensional sphere under random forcing irregular in space and time deducing the existence of an invariant measure.

The main objective of this article is to establish the convergence of the martingale solution of the stochastic Navier–Stokes equations (SNSE) on a thin spherical domain $Q_\varepsilon$, whose existence can be established as in the forthcoming paper [7] to the martingale solution of the stochastic Navier–Stokes equations on a two dimensional sphere ${\mathbb {S}^2}$ [9] as thickness $\varepsilon$ of the spherical domain converges to zero. In this way we also give another proof for the existence of a martingale solution for stochastic NSE on the unit sphere ${\mathbb {S}^2}$.

We study the stochastic Navier–Stokes equations (SNSE) for incompressible fluid

1 $$\begin{aligned} d \widetilde{u}_\varepsilon - [ \nu \varDelta \widetilde{u}_\varepsilon - (\widetilde{u}_\varepsilon \cdot \nabla ) \widetilde{u}_\varepsilon - \nabla \widetilde{p}_\varepsilon ]dt = \widetilde{f}_\varepsilon dt + \widetilde{G}_\varepsilon \, d\widetilde{W}_\varepsilon (t)&\quad \text { in } Q_\varepsilon \times (0,T), \end{aligned}$$

2 $$\begin{aligned} \mathrm{div}\;\widetilde{u}_\varepsilon = 0&\quad \text { in } Q_\varepsilon \times (0,T), \end{aligned}$$

in thin spherical shells

3 $$\begin{aligned} Q_\varepsilon := \left\{ \mathbf{y} \in \mathbb {R}^3 : 1 \le |\mathbf{y}| \le 1+ \varepsilon \right\} , \text { where } 0< \varepsilon < 1/2, \end{aligned}$$

along with free boundary conditions

4 $$\begin{aligned} \widetilde{u}_\varepsilon \cdot \mathbf {n} = 0, \quad \mathrm{curl}\;\widetilde{u}_\varepsilon \times \mathbf {n} = 0&\quad \text { on } \partial Q_\varepsilon \times (0,T), \end{aligned}$$

5 $$\begin{aligned} \widetilde{u}_\varepsilon (0, \cdot ) = \widetilde{u}_0^\varepsilon&\quad \text { in } Q_\varepsilon . \end{aligned}$$

In the above, $\widetilde{u}_\varepsilon =(\widetilde{u}_\varepsilon ^r, \widetilde{u}_\varepsilon ^\lambda , \widetilde{u}_\varepsilon ^\varphi )$ is the fluid velocity field, *p* is the pressure, $\nu >0$ is a (fixed) kinematic viscosity, $\widetilde{u}_0^\varepsilon$ is a divergence free vector field on $Q_\varepsilon$ and $\mathbf {n}$ is the unit outer normal vector to the boundary $\partial Q_\varepsilon$ and $\widetilde{W}_\varepsilon (t)$, $t \ge 0$ is an $\mathbb {R}^N$-valued Wiener process in some probability space $\left( \varOmega , \mathcal {F}, \mathbb {F}, \mathbb {P}\right)$ to be defined precisely later.

The main result of this article is Theorem 3, which establishes the convergence of the radial averages of the martingale solution (see Definition 1) of the 3D stochastic equations (1)–(5), as the thickness of the shell $\varepsilon \rightarrow 0$, to a martingale solution *u* (see Definition 2) of the following stochastic Navier–Stokes equations on the unit sphere ${\mathbb {S}^2}\subset {\mathbb R}^3$:

6 $$\begin{aligned} du - \left[ \nu \varvec{{\Delta }}^\prime \, u - (u \cdot \nabla ^\prime ) u - \nabla ^\prime p \right] dt = f dt + G\,dW&\quad \text { in } {\mathbb {S}^2}\times (0,T), \end{aligned}$$

7 $$\begin{aligned} \mathrm{{div}^\prime }u = 0&\quad \text { in } {\mathbb {S}^2}\times (0,T), \end{aligned}$$

8 $$\begin{aligned} u(0, \cdot ) = u_0&\quad \text { in } {\mathbb {S}^2}, \end{aligned}$$

where $u=(u_\lambda , u_\varphi )$ and $\varvec{{\Delta }}^\prime \,$, $\nabla ^\prime$ are the Laplace–de Rham operator and the surface gradient on ${\mathbb {S}^2}$ respectively. Assumptions on initial data and external forcing will be specified later.

The paper is organised as follows. We introduce necessary functional spaces in Sect. 2. In Sect. 3, we define some averaging operators and give their properties. Stochastic Navier–Stokes equations on thin spherical domains are introduced in Sect. 4 and a priori estimates for the radially averaged velocity are obtained which are later used to prove the convergence of the radial average of a martingale solution of stochastic NSE on thin spherical shell (see (1)–(5)) to a martingale solution of the stochastic NSE on the sphere (see (6)–(8)) with vanishing thickness.

## Preliminaries

A point $\mathbf{y}\in Q_\varepsilon$ could be represented by the Cartesian coordinates $\mathbf{y}= \left( x, y, z\right)$ or $\mathbf{y}= \left( r, \lambda , \varphi \right)$ in spherical coordinates, where

$$\begin{aligned} x = r \sin \lambda \cos \varphi \quad y = r \sin \lambda \sin \varphi \quad z = r \cos \lambda , \end{aligned}$$

for $$r \in (1, 1+\varepsilon )$$, $$\lambda \in [0,\pi ]$$ and $$\varphi \in [0, 2 \pi )$$.

For $p \in [1, \infty )$, by $L^p(Q_\varepsilon )$, we denote the Banach space of (equivalence-classes of) Lebesgue measurable $\mathbb {R}$-valued *p*th power integrable functions on $Q_\varepsilon$. The $\mathbb {R}^3$-valued *p*th power integrable vector fields will be denoted by $\mathbb {L}^p(Q_\varepsilon )$. The norm in $\mathbb {L}^p(Q_\varepsilon )$ is given by

$$\begin{aligned} \Vert u\Vert _{\mathbb {L}^p(Q_\varepsilon )} := \left( \int _{Q_\varepsilon }|u(\mathbf{y})|^p\,d\mathbf{y}\right) ^{1/p}, \qquad u \in \mathbb {L}^p(Q_\varepsilon ). \end{aligned}$$

If $p=2$, then $\mathbb {L}^2(Q_\varepsilon )$ is a Hilbert space with the inner product given by

$$\begin{aligned} \left( u, {\mathrm {v}}\right) _{{\mathbb {L}^2(Q_\varepsilon )}} := \int _{Q_\varepsilon } u(\mathbf{y}) \cdot {\mathrm {v}}(\mathbf{y})\,d\mathbf{y}, \qquad u, {\mathrm {v}}\in {\mathbb {L}^2(Q_\varepsilon )}.\end{aligned}$$

By $\mathbb {H}^1(Q_\varepsilon ) = \mathbb {W}^{1,2}(Q_\varepsilon )$, we will denote the Sobolev space consisting of all $u \in {\mathbb {L}^2(Q_\varepsilon )}$ for which there exist weak derivatives $D_i u \in {\mathbb {L}^2(Q_\varepsilon )}$, $i = 1,2,3$. It is a Hilbert space with the inner product given by

$$\begin{aligned} \left( u, {\mathrm {v}}\right) _{\mathbb {H}^1(Q_\varepsilon )} := \left( u, {\mathrm {v}}\right) _{\mathbb {L}^2(Q_\varepsilon )}+ \left( \nabla u, \nabla {\mathrm {v}}\right) _{\mathbb {L}^2(Q_\varepsilon )}, \qquad u, {\mathrm {v}}\in \mathbb {H}^1(Q_\varepsilon ), \end{aligned}$$

where

$$\begin{aligned} \left( \nabla u, \nabla {\mathrm {v}}\right) _{\mathbb {L}^2(Q_\varepsilon )}= \sum _{i=1}^3\int _{Q_\varepsilon } D_i u(\mathbf{y}) \cdot D_i {\mathrm {v}}(\mathbf{y})\,d\mathbf{y}. \end{aligned}$$

The Lebesgue and Sobolev spaces on the sphere ${\mathbb {S}^2}$ will be denoted by $\mathbb {L}^p({\mathbb {S}^2})$ and $\mathbb {W}^{s,q}({\mathbb {S}^2})$ respectively for $p, q \ge 1$ and $s \ge 0$. In particular, we will write $\mathbb {H}^1({\mathbb {S}^2})$ for $\mathbb {W}^{1,2}({\mathbb {S}^2})$.

### Functional Setting on the Shell $Q_\varepsilon$

We will use the following classical spaces on $Q_\varepsilon :$

$$\begin{aligned} \mathrm { H}_\varepsilon&= \left\{ u \in {\mathbb {L}^2(Q_\varepsilon )}:\mathrm{div}\;u = 0 \text{ in } Q_\varepsilon ,\,\, u \cdot \mathbf {n} = 0\,\, \text{ on } \partial Q_\varepsilon \right\} ,\\ \mathrm { V}_\varepsilon&= \left\{ u \in \mathbb {H}^1(Q_\varepsilon ) :\mathrm{div}\;u = 0 \text{ in } Q_\varepsilon ,\,\, u \cdot \mathbf {n} = 0\,\, \text{ on } \partial Q_\varepsilon \right\} ,\\&= \mathbb {H}^1(Q_\varepsilon ) \cap \mathrm { H}_\varepsilon . \end{aligned}$$

On $\mathrm { H}_\varepsilon$, we consider the inner product and the norm inherited from ${\mathbb {L}^2(Q_\varepsilon )}$ and denote them by $\left( \cdot , \cdot \right) _{\mathrm { H}_\varepsilon }$ and $\Vert \cdot \Vert _{\mathrm { H}_\varepsilon }$ respectively, that is

$$\begin{aligned} \left( u, {\mathrm {v}}\right) _{\mathrm { H}_\varepsilon } := \left( u, {\mathrm {v}}\right) _{\mathbb {L}^2(Q_\varepsilon )}, \qquad \Vert u\Vert _{\mathrm { H}_\varepsilon } := \Vert u\Vert _{\mathbb {L}^2(Q_\varepsilon )},\qquad u,{\mathrm {v}}\in \mathrm { H}_\varepsilon . \end{aligned}$$

Let us define a bilinear map $\mathrm {a}_\varepsilon :\mathrm { V}_\varepsilon \times \mathrm { V}_\varepsilon \rightarrow \mathbb {R}$ by

9 $$\begin{aligned} \mathrm {a}_\varepsilon (u,{\mathrm {v}}) := \left( \mathrm{curl}\;u, \mathrm{curl}\;{\mathrm {v}}\right) _{\mathbb {L}^2(Q_\varepsilon )},\qquad u,{\mathrm {v}}\in \mathrm { V}_\varepsilon , \end{aligned}$$

where

$$\begin{aligned}\mathrm{curl}\;u = \nabla \times u,\end{aligned}$$

and for $u \in \mathrm { V}_\varepsilon$, we define

10 $$\begin{aligned} \Vert u\Vert _{\mathrm { V}_\varepsilon }^2 := \mathrm {a}_\varepsilon (u,u) = \Vert \mathrm{curl}\;u\Vert ^2_{{\mathbb {L}^2(Q_\varepsilon )}}. \end{aligned}$$

Note that for $u \in \mathrm { V}_\varepsilon$, $\Vert u\Vert _{\mathrm { V}_\varepsilon } = 0$ implies that *u* is a constant vector and $u\cdot \mathbf {n} = 0$ on $\partial Q_\varepsilon$ i.e., *u* is tangent to $Q_\varepsilon$ for every $\mathbf{y}\in \partial Q_\varepsilon$, and thus must be 0. Hence $\Vert \cdot \Vert _{\mathrm { V}_\varepsilon }$ is a norm on $\mathrm { V}_\varepsilon$ (other properties can be verified easily). Under this norm $\mathrm { V}_\varepsilon$ is a Hilbert space with the inner product given by

$$\begin{aligned} \left( u, {\mathrm {v}}\right) _{\mathrm { V}_\varepsilon } := \left( \mathrm{curl}\;u, \mathrm{curl}\;{\mathrm {v}}\right) _{{\mathbb {L}^2(Q_\varepsilon )}}, \qquad u, {\mathrm {v}}\in \mathrm { V}_\varepsilon . \end{aligned}$$

We denote the dual pairing between $\mathrm { V}_\varepsilon$ and $\mathrm { V}^\prime _\varepsilon$ by $\langle \cdot , \cdot \rangle _\varepsilon$, that is $\langle \cdot , \cdot \rangle _\varepsilon := {}_{\mathrm { V}_\varepsilon ^\prime }\langle \cdot , \cdot \rangle _{\mathrm { V}_\varepsilon }$. By the Lax–Milgram theorem, there exists a unique bounded linear operator $\mathcal {A}_\varepsilon :\mathrm { V}_\varepsilon \rightarrow \mathrm { V}_\varepsilon ^\prime$ such that we have the following equality$:$

11 $$\begin{aligned} \langle \mathcal {A}_\varepsilon u, {\mathrm {v}}\rangle _\varepsilon = \left( u,{\mathrm {v}}\right) _{\mathrm { V}_\varepsilon }, \qquad u, {\mathrm {v}}\in \mathrm { V}_\varepsilon . \end{aligned}$$

The operator $\mathcal {A}_\varepsilon$ is closely related to the Stokes operator $\mathrm { A}_\varepsilon$ defined by

12 $$\begin{aligned} \begin{aligned} \mathrm {D}(\mathrm { A}_\varepsilon )&= \left\{ u \in \mathrm { V}_\varepsilon : \mathcal {A}_\varepsilon u \in \mathrm { H}_\varepsilon , \; \mathrm{curl}\;u \times \mathbf {n} = 0 \text{ on } \partial Q_\varepsilon \right\} ,\\ \mathrm {A}_\varepsilon u&= \mathcal {A}_\varepsilon u, \qquad u \in \mathrm {D}(\mathrm { A}_\varepsilon ). \end{aligned} \end{aligned}$$

The Stokes operator $\mathrm { A}_\varepsilon$ is a non-negative self-adjoint operator in $\mathrm { H}_\varepsilon$ (see Appendix B). Also note that

$$\begin{aligned} \mathrm {D}(\mathrm { A}_\varepsilon ) = \left\{ u \in \mathbb {H}^2(Q_\varepsilon ) :\mathrm{div}\;u = 0 \text{ in } Q_\varepsilon ,\,\, u \cdot \mathbf {n} = 0 \text{ and } \mathrm{curl}\;u \times \mathbf {n} = 0 \text{ on } \partial Q_\varepsilon \right\} . \end{aligned}$$

We recall the Leray–Helmholtz projection operator $P_\varepsilon$, which is the orthogonal projector of ${\mathbb {L}^2(Q_\varepsilon )}$ onto $\mathrm { H}_\varepsilon$. Using this, the Stokes operator $\mathrm { A}_\varepsilon$ can be characterised as follows$:$

13 $$\begin{aligned} \mathrm { A}_\varepsilon u = P_\varepsilon (-\varDelta u), \qquad u \in \mathrm {D}(\mathrm { A}_\varepsilon ). \end{aligned}$$

We also have the following characterisation of the Stokes operator $\mathrm { A}_\varepsilon$ [53, Lemma 1.1]$:$

14 $$\begin{aligned} \mathrm { A}_\varepsilon u =\mathrm {curl} \left( \mathrm{curl}\;u\right) , \qquad u \in \mathrm {D}(\mathrm { A}_\varepsilon ). \end{aligned}$$

For $u \in \mathrm { V}_\varepsilon$, ${\mathrm {v}}\in \mathrm {D}(\mathrm { A}_\varepsilon )$, we have the following identity (see Lemma 28)

15 $$\begin{aligned} \left( \mathrm{curl}\;u, \mathrm{curl}\;{\mathrm {v}}\right) _{{\mathbb {L}^2(Q_\varepsilon )}} = \left( u, \mathrm { A}_\varepsilon {\mathrm {v}}\right) _{{\mathbb {L}^2(Q_\varepsilon )}}. \end{aligned}$$

Let $b_\varepsilon$ be the continuous trilinear from on $\mathrm { V}_\varepsilon$ defined by$:$

16 $$\begin{aligned} b_\varepsilon (u, {\mathrm {v}}, {\mathrm {w}}) = \int _{Q_\varepsilon }\left( u \cdot \nabla \right) {\mathrm {v}}\cdot {\mathrm {w}}\,d\mathbf{y},\qquad u,{\mathrm {v}},{\mathrm {w}}\in \mathrm { V}_\varepsilon . \end{aligned}$$

We denote by $B_\varepsilon$ the bilinear mapping from $\mathrm { V}_\varepsilon \times \mathrm { V}_\varepsilon$ to $\mathrm { V}_\varepsilon ^\prime$ by

$$\begin{aligned} \left\langle B_\varepsilon (u,{\mathrm {v}}), {\mathrm {w}}\right\rangle _\varepsilon = b_\varepsilon (u,{\mathrm {v}},{\mathrm {w}}), \qquad u,{\mathrm {v}}, {\mathrm {w}}\in \mathrm { V}_\varepsilon , \end{aligned}$$

and we set

$$\begin{aligned}B_\varepsilon (u) = B_\varepsilon (u,u).\end{aligned}$$

Let us also recall the following properties of the form $b_\varepsilon$, which directly follows from the definition of $b_\varepsilon$ $:$

17 $$\begin{aligned} b_\varepsilon (u,{\mathrm {v}},{\mathrm {w}}) = - b_\varepsilon (u,{\mathrm {w}},{\mathrm {v}}),\qquad u,{\mathrm {v}}, {\mathrm {w}}\in \mathrm { V}_\varepsilon . \end{aligned}$$

In particular,

18 $$\begin{aligned} \left\langle B_\varepsilon (u,{\mathrm {v}}), {\mathrm {v}}\right\rangle _\varepsilon = b_\varepsilon (u, {\mathrm {v}}, {\mathrm {v}}) = 0, \qquad u,{\mathrm {v}}\in \mathrm { V}_\varepsilon . \end{aligned}$$

### Functional Setting on the Sphere ${\mathbb {S}^2}$

Let $s\ge 0$. The Sobolev space $H^s({\mathbb {S}^2})$ is the space of all scalar functions $\psi \in L^2({\mathbb {S}^2})$ such that $(-{\varDelta ^\prime })^{s/2} \psi \in L^2({\mathbb {S}^2})$, where ${\varDelta ^\prime }$ is the Laplace–Beltrami operator on the sphere (see (177)). We similarly define $\mathbb {H}^s({\mathbb {S}^2})$ as the space of all vector fields $u \in \mathbb {L}^2({\mathbb {S}^2})$ such that $(-\varvec{{\Delta }}^\prime \, )^{s/2} u \in {\mathbb {L}^2({\mathbb {S}^2})}$, where $\varvec{{\Delta }}^\prime \,$ is the Laplace–de Rham operator on the sphere (see (180)).

For $s \ge 0$, $\left( H^s({\mathbb {S}^2}), \Vert \cdot \Vert _{H^{s}({\mathbb {S}^2})}\right)$ and $\left( \mathbb {H}^s({\mathbb {S}^2}), \Vert \cdot \Vert _{\mathbb {H}^{s}({\mathbb {S}^2})}\right)$ are Hilbert spaces under the respective norms, where

19 $$\begin{aligned} \Vert \psi \Vert ^2_{H^s({\mathbb {S}^2})} = \Vert \psi \Vert ^2_{L^2({\mathbb {S}^2})} + \Vert (-{\varDelta ^\prime })^{s/2} \psi \Vert ^2_{L^2({\mathbb {S}^2})}, \qquad \psi \in H^s({\mathbb {S}^2}) \end{aligned}$$

and

20 $$\begin{aligned} \Vert u\Vert ^2_{\mathbb {H}^s({\mathbb {S}^2})} = \Vert u\Vert ^2_{\mathbb {L}^2({\mathbb {S}^2})} + \Vert (-\varvec{{\Delta }}^\prime \, )^{s/2} u\Vert ^2_{\mathbb {L}^2({\mathbb {S}^2})}, \qquad u \in \mathbb {H}^s({\mathbb {S}^2}). \end{aligned}$$

By the Hodge decomposition theorem [3, Theorem 1.72] the space of $C^\infty$ smooth vector fields on ${\mathbb {S}^2}$ can be decomposed into three components:

21 $$\begin{aligned} C^\infty (T{\mathbb {S}^2}) = \mathcal {G}\oplus \mathcal {V}\oplus \mathcal {H}, \end{aligned}$$

where

22 $$\begin{aligned} \mathcal {G}= \{\nabla ^\prime \psi : \psi \in C^\infty ({\mathbb {S}^2})\},\quad \mathcal {V}= \{\mathrm{curl}^\prime \psi : \psi \in C^\infty ({\mathbb {S}^2})\}, \end{aligned}$$

and $\mathcal {H}$ is the finite-dimensional space of harmonic vector fields. Since the sphere is simply connected, $\mathcal {H}= \{0\}$. We introduce the following spaces

$$\begin{aligned} \mathrm { H}= & {} \text{ closure } \text{ of } \mathcal {V} \text{ in } \mathbb {L}^2({\mathbb {S}^2}), \\ \mathrm { V}= & {} \text{ closure } \text{ of } \mathcal {V} \text{ in } \mathbb {H}^1({\mathbb {S}^2}). \end{aligned}$$

Note that it is known (see [50])

$$\begin{aligned} \mathrm { H}= & {} \{u \in \mathbb {L}^2({\mathbb {S}^2}): \mathrm{div}^\prime u =0\}, \\ \mathrm { V}= & {} \mathrm { H}\cap \mathbb {H}^1({\mathbb {S}^2}). \end{aligned}$$

Given a tangential vector field *u* on ${\mathbb {S}^2}$, we can find vector field $\tilde{u}$ defined on some neighbourhood of ${\mathbb {S}^2}$ such that their restriction to ${\mathbb {S}^2}$ is equal to *u*, that is $\tilde{u}\vert _{{\mathbb {S}^2}} = u \in T{\mathbb {S}^2}$. Then we define

23 $$\begin{aligned} \mathrm{curl}^\prime u (\mathbf{x}) := (\mathbf{x}\cdot (\nabla \times \tilde{u}))\vert _{{\mathbb {S}^2}} = (\mathbf{x}\cdot \mathrm{curl\;} \tilde{u})\vert _{{\mathbb {S}^2}}. \end{aligned}$$

Since $\mathbf{x}$ is orthogonal to the tangent plane $T_{\mathbf{x}} {\mathbb {S}^2}$, $\mathrm{curl}^\prime \; u$ is the normal component of $\nabla \times \tilde{u}$. It could be identified with a normal vector field when needed.

We define the bilinear form $a: \mathrm { V}\times \mathrm { V}\rightarrow \mathbb {R}$ by

$$\begin{aligned} a(u,{\mathrm {v}}) := (\mathrm{curl}^\prime u, \mathrm{curl}^\prime {\mathrm {v}})_{\mathbb {L}^2({\mathbb {S}^2})}, \qquad u, {\mathrm {v}}\in \mathrm { V}. \end{aligned}$$

The bilinear from *a* satisfies $a(u,{\mathrm {v}}) \le \Vert u\Vert _{\mathbb {H}^1({\mathbb {S}^2})} \Vert {\mathrm {v}}\Vert _{\mathbb {H}^1({\mathbb {S}^2})}$ and hence is continuous on $\mathrm { V}$. So by the Riesz representation theorem, there exists a unique operator $\mathcal {A}: \mathrm { V}\rightarrow \mathrm { V}^\prime$ such that $a(u, {\mathrm {v}}) = {}_{\mathrm { V}^\prime }\langle \mathcal {A}u, {\mathrm {v}}\rangle _{\mathrm { V}}$ for $u, {\mathrm {v}}\in \mathrm { V}$. Using the Poincaré inequality, we also have $a(u,u) \ge \alpha \Vert u\Vert ^2_{\mathrm { V}}$, for some positive constant $\alpha$, which means *a* is coercive in $\mathrm { V}$. Hence, by the Lax–Milgram theorem, the operator $\mathcal {A}: \mathrm { V}\rightarrow \mathrm { V}^\prime$ is an isomorphism.

Next we define an operator $\mathrm { A}$ in $\mathrm { H}$ as follows:

24 $$\begin{aligned} {\left\{ \begin{array}{ll} \mathrm {D}(\mathrm { A}) &{}:= \{ u \in \mathrm { V}: \mathcal {A}u \in \mathrm { H}\}, \\ \mathrm { A}u &{}:= \mathcal {A}u, \quad u \in \mathrm {D}(\mathrm { A}). \end{array}\right. } \end{aligned}$$

By Cattabriga [15], see also Temam [49, p. 56], one can show that $\mathrm { A}$ is a non-negative self-adjoint operator in $\mathrm { H}$. Moreover, $\mathrm { V}= \mathrm {D}(\mathrm { A}^{1/2})$, see [49, p. 57].

Let *P* be the orthogonal projection from ${\mathbb {L}^2({\mathbb {S}^2})}$ to $\mathrm { H}$, called the Leray–Helmholtz projection. It can be shown, see [19, p. 104], that

25 $$\begin{aligned} \mathrm {D}(\mathrm { A}) = \mathbb {H}^2({\mathbb {S}^2}) \cap \mathrm { H}, \qquad \mathrm { A}u = P\left( -\varvec{{\Delta }}^\prime \, u\right) , \quad u \in \mathrm {D}(\mathrm { A}). \end{aligned}$$

$\mathrm {D}(\mathrm { A})$ along with the graph norm

$$\begin{aligned} \Vert u\Vert _{\mathrm {D}(\mathrm { A})}^2 := \Vert u\Vert _{{\mathbb {L}^2({\mathbb {S}^2})}}^2 + \Vert \mathrm { A}u\Vert ^2_{{\mathbb {L}^2({\mathbb {S}^2})}}, \quad u \in \mathrm {D}(\mathrm { A}), \end{aligned}$$

forms a Hilbert space with the inner product

$$\begin{aligned} \langle u, {\mathrm {v}}\rangle _{\mathrm {D}(\mathrm { A})} := \left( u, {\mathrm {v}}\right) _{\mathbb {L}^2({\mathbb {S}^2})}+ \left( \mathrm { A}u, \mathrm { A}{\mathrm {v}}\right) _{\mathbb {L}^2({\mathbb {S}^2})}, \quad u, {\mathrm {v}}\in \mathrm {D}(\mathrm { A}). \end{aligned}$$

Note that $\mathrm {D}(\mathrm { A})$-norm is equivalent to $\mathbb {H}^2({\mathbb {S}^2})$-norm. For more details about the Stokes operator on the sphere and fractional power $\mathrm { A}^{s}$ for $s \ge 0$, see [9, Sec. 2.2].

Given two tangential vector fields *u* and ${\mathrm {v}}$ on ${\mathbb {S}^2}$, we can find vector fields $\tilde{u}$ and $\tilde{{\mathrm {v}}}$ defined on some neighbourhood of ${\mathbb {S}^2}$ such that their restrictions to ${\mathbb {S}^2}$ are equal to, respectively, *u* and ${\mathrm {v}}$. Then we define the covariant derivative

$$\begin{aligned}{}[\nabla ^\prime _{{\mathrm {v}}} u](\mathbf{x}) = \pi _{\mathbf{x}} \left( \sum _{i=1}^3 \tilde{{\mathrm {v}}}_i(\mathbf{x}) \partial _i \tilde{u}(\mathbf{x})\right) = \pi _\mathbf{x}( (\tilde{{\mathrm {v}}}(\mathbf{x}) \cdot \nabla ) \tilde{u}(\mathbf{x}) ), \quad \mathbf{x}\in {\mathbb {S}^2}, \end{aligned}$$

where $\pi _\mathbf{x}$ is the orthogonal projection from $\mathbb {R}^3$ onto the tangent space $T_\mathbf{x}{\mathbb {S}^2}$ to ${\mathbb {S}^2}$ at $\mathbf{x}$. By decomposing $\tilde{u}$ and $\tilde{{\mathrm {v}}}$ into tangential and normal components and using orthogonality, one can show that

26 $$\begin{aligned} \pi _\mathbf{x}( \tilde{u} \times \tilde{{\mathrm {v}}}) = u \times ( (\mathbf{x}\cdot {\mathrm {v}}) x) + (\mathbf{x}\cdot u)\mathbf{x}\times {\mathrm {v}}= u \times ( (\mathbf{x}\cdot v) \mathbf{x}), \quad \mathbf{x}\in {\mathbb {S}^2}, \end{aligned}$$

where in the last equality, we use the fact that $\mathbf{x}\cdot {\mathrm {v}}= 0$ for any tangential vector ${\mathrm {v}}$.

We set ${\mathrm {v}}= u$ and use the formula

$$\begin{aligned} (\tilde{u} \cdot \nabla )\tilde{u} = \nabla \frac{|\tilde{u}|^2}{2} - \tilde{u} \times (\nabla \times \tilde{u}) \end{aligned}$$

to obtain

$$\begin{aligned} {[}\nabla ^\prime _u u](\mathbf{x}) = \nabla ^\prime \frac{|u|^2}{2} - \pi _\mathbf{x}\left( \tilde{u} \times (\nabla \times \tilde{u})\right) . \end{aligned}$$

Using (26) for the vector fields $\tilde{u}$ and $\tilde{{\mathrm {v}}} = \nabla \times \tilde{u} =\mathrm{curl}\;\tilde{u}$, we have

$$\begin{aligned} \pi _\mathbf{x}( \tilde{u} \times (\nabla \times \tilde{u})) = u \times ( (\mathbf{x}\cdot \mathrm{curl } u)\mathbf{x}) = u \times \mathrm{curl }^\prime u. \end{aligned}$$

Thus

$$\begin{aligned} \nabla ^\prime _u u = \nabla ^\prime \frac{|u|^2}{2} - u \times \mathrm{curl }^\prime u. \end{aligned}$$

We consider the trilinear form *b* on $\mathrm { V}\times \mathrm { V}\times \mathrm { V}$, defined by

27 $$\begin{aligned} b({\mathrm {v}}, {\mathrm {w}}, z) = (\nabla ^\prime _{{\mathrm {v}}} {\mathrm {w}},z) = \int _{{\mathbb {S}^2}} \nabla ^\prime _{{\mathrm {v}}} {\mathrm {w}}\cdot z\,d\sigma (\mathbf{x}), \quad {\mathrm {v}}, {\mathrm {w}}, z \in \mathrm { V}, \end{aligned}$$

where $d \sigma (\mathbf {x})$ is the surface measure on $\mathbb {S}^2$.

## Averaging Operators and Their Properties

In this section we recall the averaging operators which were first introduced by Raugel and Sell [44, 45] for thin domains. Later, Temam and Ziane [53] adapted those averaging operators to thin spherical domains, introduced some additional operators and proved their properties using the spherical coordinate system. Recently, Saito [46] used these averaging operators to study Boussinesq equations in thin spherical domains. We closely follow [46, 53] to describe our averaging operators and provide proofs for some of the properties mentioned below.

Let $M_\varepsilon :\mathcal {C}(Q_\varepsilon , \mathbb {R}) \rightarrow \mathcal {C}({\mathbb {S}^2}, \mathbb {R})$ be a map that projects functions defined on $Q_\varepsilon$ to functions defined on ${\mathbb {S}^2}$ and is defined by

28 $$\begin{aligned} M_\varepsilon \psi (\mathbf{x}) := \dfrac{1}{\varepsilon } \int _1^{1+\varepsilon } r \psi (r \mathbf{x})\,dr, \qquad \mathbf{x}\in {\mathbb {S}^2}. \end{aligned}$$

### Remark 1

We will use the Cartesian and spherical coordinates interchangeably in this paper. For example, if $\mathbf{x}\in {\mathbb {S}^2}$ then we will identify it by $\mathbf{x}= (\lambda , \varphi )$ where $\lambda \in [0, \pi ]$ and $\varphi \in [0, 2 \pi )$.

### Lemma 1

The map $M_\varepsilon$ as defined in (28) is continuous (and linear) w.r.t norms $L^2(Q_\varepsilon )$ and $L^2({\mathbb {S}^2})$. Moreover,

29 $$\begin{aligned} \Vert M_\varepsilon \psi \Vert _{L^2({\mathbb {S}^2})}^2 \le \frac{1}{\varepsilon }\Vert \psi \Vert ^2_{L^2(Q_\varepsilon )}, \qquad \psi \in L^2(Q_\varepsilon ). \end{aligned}$$

### Proof

Take $\psi \in \mathcal {C}({Q}_\varepsilon )$ then by the definition of $M_\varepsilon$ we have

$$\begin{aligned} M_\varepsilon \psi (\mathbf{x}) = \dfrac{1}{\varepsilon } \int _1^{1+\varepsilon } r \psi (r\mathbf{x})\,dr. \end{aligned}$$

Thus, using the Cauchy–Schwarz inequality we have

$$\begin{aligned} \Vert M_\varepsilon \psi \Vert _{L^2({\mathbb {S}^2})}^2&= \int _{\mathbb {S}^2}|M_\varepsilon \psi (\mathbf{x})|^2\,d\sigma (\mathbf{x}) = \int _{\mathbb {S}^2}\left| \dfrac{1}{\varepsilon } \int _{1}^{1+\varepsilon } r \psi (r \mathbf{x})\,dr\right| ^2\,d\sigma (\mathbf{x}) \\&\le \dfrac{1}{\varepsilon ^2} \int _{\mathbb {S}^2}\left( \int _{1}^{1+\varepsilon }r^2 |\psi (r \mathbf{x})|^2\,dr \int _{1}^{1+\varepsilon }dr \right) \,d \sigma (\mathbf{x}) \\&= \dfrac{1}{\varepsilon ^2} \cdot \varepsilon \int _{1}^{1+\varepsilon }\int _{\mathbb {S}^2}r^2 |\psi (r \mathbf{x})|^2\,dr\,d\sigma (\mathbf{x}) = \frac{1}{\varepsilon } \Vert \psi \Vert ^2_{L^2(Q_\varepsilon )}, \end{aligned}$$

where the last equality follows from the fact that

$$\begin{aligned} \int _{Q_\varepsilon } d\mathbf{y}= \int _{1}^{1+\varepsilon }\int _{\mathbb {S}^2}r^2\,dr\,d\sigma (\mathbf{x}) \end{aligned}$$

is the volume integral over the spherical shell $Q_\varepsilon$ in spherical coordinates, with

$$\begin{aligned} d\sigma (\mathbf{x}) = \sin {\lambda }\,d\lambda \,d\varphi , \end{aligned}$$

being the Lebesgue measure over a unit sphere. Therefore, we obtain

30 $$\begin{aligned} \Vert M_\varepsilon \psi \Vert ^2_{L^2({\mathbb {S}^2})} \le \frac{1}{\varepsilon }\Vert \psi \Vert ^2_{L^2(Q_\varepsilon )}, \end{aligned}$$

and hence the map is bounded and we can infer (29). $\square$

### Corollary 1

The map $M_\varepsilon$ as defined in (28) has a unique extension, which without the abuse of notation will be denoted by the same symbol $M_\varepsilon :L^2(Q_\varepsilon ) \rightarrow L^2({\mathbb {S}^2})$.

### Proof

Since $\mathcal {C}(Q_\varepsilon )$ is dense in $L^2(Q_\varepsilon )$ and $M_\varepsilon :L^2(Q_\varepsilon ) \rightarrow L^2({\mathbb {S}^2})$ is a bounded map thus by the Riesz representation theorem there exists a unique extension. $\square$

### Lemma 2

The following map

31 $$\begin{aligned} {R}_\varepsilon :L^2({\mathbb {S}^2}) \ni \psi \mapsto \frac{1}{|\cdot |} \psi \left( \frac{\cdot }{|\cdot |}\right) \in L^2(Q_\varepsilon ) \end{aligned}$$

is bounded and

$$\begin{aligned} \Vert {R}_\varepsilon \Vert ^2_{\mathcal {L}(L^2({\mathbb {S}^2}), L^2(Q_\varepsilon ))} = \varepsilon . \end{aligned}$$

### Proof

It is sufficient to consider $\psi \in \mathcal {C}({\mathbb {S}^2})$. For $\psi \in \mathcal {C}({\mathbb {S}^2})$, we have

$$\begin{aligned} \Vert {R}_\varepsilon \psi \Vert _{L^2(Q_\varepsilon )}^2 = \int _{Q_\varepsilon }|{\mathrm {v}}(\mathbf{y})|^2\,d\mathbf{y}= \int _{1}^{1+\varepsilon }r^2 \int _{{\mathbb {S}^2}} |{\mathrm {v}}(r\mathbf{x})|^2\,d\sigma (\mathbf{x})\,dr, \end{aligned}$$

where ${\mathrm {v}}(\mathbf{y}) = \frac{1}{|\mathbf{y}|}\psi \left( \frac{\mathbf{y}}{|\mathbf{y}|}\right)$. But, for $\mathbf{x}\in {\mathbb {S}^2}$

$$\begin{aligned} {\mathrm {v}}(r\mathbf{x}) = \frac{1}{|r\mathbf{x}|}\psi \left( \frac{r\mathbf{x}}{|r\mathbf{x}|}\right) = \frac{1}{r}\psi (\mathbf{x}). \end{aligned}$$

So

$$\begin{aligned} \Vert {R}_\varepsilon \psi \Vert _{L^2(Q_\varepsilon )}^2&= \int _{1}^{1+\varepsilon }r^2 \frac{1}{r^2} \int _{\mathbb {S}^2}|\psi (\mathbf{x})|^2\,d \sigma (\mathbf{x})\,dr = \varepsilon \Vert \psi \Vert ^2_{L^2({\mathbb {S}^2})}, \end{aligned}$$

thus, showing that the map ${R}_\varepsilon$ is bounded w.r.t. $L^2({\mathbb {S}^2})$ and $L^2(Q_\varepsilon )$ norms. $\square$

### Lemma 3

Let $\psi \in W^{1,p}({\mathbb {S}^2})$ for $p \ge 2$. Then for $\varepsilon \in (0,1)$ there exists a constant $C > 0$ independent of $\varepsilon$ such that

$$\begin{aligned} \Vert \nabla {R}_\varepsilon \psi \Vert ^p_{L^p(Q_\varepsilon )} \le C \varepsilon \Vert \psi \Vert _{W^{1,p}({\mathbb {S}^2})}^p. \end{aligned}$$

### Proof

By the definition of the map ${R}_\varepsilon$ (see (31)), and identities (172), (176) for the scalar function $\psi \in W^{1,p}({\mathbb {S}^2})$, we have for $Q_\varepsilon \ni \mathbf{y}= r\mathbf{x}$, $r \in (1,1+\varepsilon )$ and $\mathbf{x}\in {\mathbb {S}^2}$,

$$\begin{aligned} \nabla ({R}_\varepsilon [\psi ](\mathbf{y}))&= \frac{\partial }{\partial r} \left( {R}_\varepsilon [\psi ](\mathbf{y})\right) \widehat{e_r} + \frac{1}{r} \frac{\partial }{\partial \lambda } \left( {R}_\varepsilon [\psi ](\mathbf{y})\right) \widehat{e_\lambda } + \frac{1}{r \sin \lambda } \frac{\partial }{\partial \varphi } \left( {R}_\varepsilon [\psi ](\mathbf{y})\right) \widehat{e_\varphi } \\&= \frac{\partial }{\partial r} \left( \frac{\psi (\mathbf{x})}{r}\right) \widehat{e_r} + \frac{1}{r} \frac{\partial }{\partial \lambda } \left( \frac{\psi (\mathbf{x})}{r}\right) \widehat{e_\lambda } + \frac{1}{r \sin \lambda } \frac{\partial }{\partial \varphi } \left( \frac{\psi (\mathbf{x})}{r}\right) \widehat{e_\varphi } \\&= - \frac{1}{r^2} \psi (\mathbf{x}) \widehat{e_r} + \frac{1}{r^2} \frac{\partial }{\partial \lambda } \left( \psi (\mathbf{x})\right) \widehat{e_\lambda } + \frac{1}{r^2 \sin \lambda } \frac{\partial }{\partial \varphi } \left( \psi (\mathbf{x})\right) \widehat{e_\varphi }. \end{aligned}$$

Hence,

$$\begin{aligned} \Vert \nabla {R}_\varepsilon \psi \Vert ^p_{L^p(Q_\varepsilon )}&= \int _{Q_\varepsilon } |\nabla ({R}_\varepsilon [\psi ](\mathbf{y}))|^p\,d\mathbf{y}\\&= \int _{1}^{1+\varepsilon }\int _{\mathbb {S}^2}\frac{1}{r^{2p}}\left( |\psi (\mathbf{x})|^p + |\nabla ^\prime \psi (\mathbf{x})|^p\right) r^2 d\sigma (\mathbf{x})dr \\&= -\frac{r^{3 - 2p}}{2p - 3}\bigg \vert ^{1+\varepsilon }_1\left( \Vert \psi \Vert ^p_{L^p({\mathbb {S}^2})} + \Vert \nabla ^\prime \psi \Vert ^p_{L^p({\mathbb {S}^2})}\right) \\&\le C(p) \varepsilon \Vert \psi \Vert ^p_{W^{1,p}({\mathbb {S}^2})}. \end{aligned}$$

$$\square $$

### Lemma 4

Let $\psi \in H^2({\mathbb {S}^2})$. Then for $\varepsilon \in (0,1)$

32 $$\begin{aligned} \Vert \varDelta {R}_\varepsilon \psi \Vert ^2_{L^2(Q_\varepsilon )} \le \varepsilon \Vert \varDelta ' \psi \Vert ^2_{L^2({\mathbb {S}^2})}. \end{aligned}$$

### Proof

Let $$\psi \in H^2({\mathbb {S}^2})$$, then

$$\begin{aligned} {[}{R}_\varepsilon \psi ](\mathbf{y}) = \frac{1}{|\mathbf{y}|} \psi \left( \frac{\mathbf{y}}{|\mathbf{y}|}\right) , \quad \mathbf{y}\in Q_\varepsilon . \end{aligned}$$

Therefore, for every $Q_\varepsilon \ni \mathbf{y}= r\mathbf{x}$, $r \in (1, 1+\varepsilon )$ and $x \in {\mathbb {S}^2}$, we have (see (171) and (177) for the definition of Laplace–Beltrami operator)

$$\begin{aligned} \varDelta ([{R}_\varepsilon \psi ](\mathbf{y}))&= \frac{\partial ^2}{\partial r^2} \left( {R}_\varepsilon \psi (\mathbf{y})\right) + \frac{2}{r} \frac{\partial }{\partial r} \left( {R}_\varepsilon \psi (\mathbf{y})\right) + \frac{1}{r^2} \varDelta ^\prime \left( {R}_\varepsilon \psi (\mathbf{y})\right) \\&= \frac{\partial ^2}{\partial r^2}\left( \frac{\psi (\mathbf{x})}{r}\right) + \frac{2}{r} \frac{\partial }{\partial r}\left( \frac{\psi (\mathbf{x})}{r}\right) + \frac{1}{r^2} \varDelta ^\prime \left( \frac{\psi (\mathbf{x})}{r}\right) \\&= \frac{2}{r^3}\psi (\mathbf{x}) - \frac{2}{r^3}\psi (\mathbf{x}) + \frac{1}{r^3}\varDelta ^\prime \psi (\mathbf{x})\\&= \frac{1}{r^3}\varDelta ^\prime \psi (\mathbf{x}). \end{aligned}$$

Hence

$$\begin{aligned} \Vert \varDelta \left( {R}_\varepsilon \psi \right) \Vert ^2_{L^2(Q_\varepsilon )}&= \int _{Q_\varepsilon } |\varDelta \left( [{R}_\varepsilon \psi ](\mathbf{y})\right) |^2 d\mathbf{y}= \int _{1}^{1+\varepsilon }\int _{\mathbb {S}^2}\frac{1}{r^6} |\varDelta ^\prime \psi (\mathbf{x})|^2 r^2 d\sigma (\mathbf{x})\,dr \\&= -\frac{1}{3r^3}\Big |_{1}^{1+\varepsilon }\Vert \varDelta ^\prime \psi \Vert ^2_{L^2({\mathbb {S}^2})} = \frac{\left( (1+\varepsilon )^3 - 1\right) }{3(1+\varepsilon )^3}\Vert \varDelta ^\prime \psi \Vert ^2_{L^2({\mathbb {S}^2})} \\&= \frac{\varepsilon ^3 + 3\varepsilon ^2 + 3\varepsilon }{(1+\varepsilon )^3}\Vert \varDelta ^\prime \psi \Vert ^2_{L^2({\mathbb {S}^2})}. \end{aligned}$$

Since $\varepsilon \in (0,1)$, the inequality (32) holds. $\square$

### Remark 2

It is easy to check that the dual operator $M_\varepsilon ^*:L^2({\mathbb {S}^2}) \rightarrow L^2(Q_\varepsilon )$ is given by

33 $$\begin{aligned} \left( M_\varepsilon ^*\psi \right) (\mathbf{y}) = \frac{1}{\varepsilon }(R_\varepsilon \psi )(\mathbf{y}), \qquad \mathbf{y}\in Q_\varepsilon . \end{aligned}$$

Next we define another map

34 $$\begin{aligned} \widehat{{M}}_\varepsilon&= {R}_\varepsilon \circ M_\varepsilon :L^2(Q_\varepsilon ) \rightarrow L^2(Q_\varepsilon ). \end{aligned}$$

Courtesy of Corollary 1 and Lemma 2, $\widehat{{M}}_\varepsilon$ is well-defined and bounded. Using definitions of maps $R_\varepsilon$ and $M_\varepsilon$ , we have

$$\begin{aligned} \widehat{{M}}_\varepsilon :\psi \mapsto \left\{ \mathbf{y}\mapsto \frac{1}{|\mathbf{y}|} \left( M_\varepsilon \psi \right) \left( \frac{\mathbf{y}}{|\mathbf{y}|}\right) \right\} . \end{aligned}$$

### Lemma 5

Let $\psi \in L^2(Q_\varepsilon )$, then we have the following scaling property

35 $$\begin{aligned} \Vert \widehat{{M}}_\varepsilon \psi \Vert ^2_{L^2(Q_\varepsilon )} = \varepsilon \Vert M_\varepsilon \psi \Vert ^2_{L^2({\mathbb {S}^2})}, \qquad \psi \in L^2(Q_\varepsilon ). \end{aligned}$$

### Proof

Let $\psi \in L^2(Q_\varepsilon )$. Then by the defintion of the map $\widehat{{M}}_\varepsilon$, we have

$$\begin{aligned} \Vert \widehat{{M}}_\varepsilon \psi \Vert ^2_{L^2(Q_\varepsilon )}&= \int _{Q_\varepsilon }|\widehat{{M}}_\varepsilon \psi (\mathbf{y})|^2\,d\mathbf{y}\\&= \int _{1}^{1+\varepsilon }\int _{\mathbb {S}^2}\frac{1}{r^2}|M_\varepsilon \psi (\mathbf{x})|^2 r^2 d\sigma (\mathbf{x})\,dr = \varepsilon \Vert M_\varepsilon \psi \Vert ^2_{L^2({\mathbb {S}^2})}. \end{aligned}$$

$$\square $$

The normal component of a function $\psi$ defined on $Q_\varepsilon$ when projected to ${\mathbb {S}^2}$ is given by the map $\widehat{N}_\varepsilon$ which is defined by

36 $$\begin{aligned} \widehat{N}_\varepsilon = \mathrm{{Id}} - \widehat{{M}}_\varepsilon , \end{aligned}$$

i.e.

$$\begin{aligned} \widehat{N}_\varepsilon :L^2(Q_\varepsilon ) \ni \psi \mapsto \psi - \widehat{{M}}_\varepsilon \psi \in L^2(Q_\varepsilon ). \end{aligned}$$

The following result establishes an important property of the map $\widehat{N}_\varepsilon$.

### Lemma 6

Let $$\psi \in L^2(Q_\varepsilon )$$, then

37 $$\begin{aligned} \int _{1}^{1+\varepsilon }r \widehat{N}_\varepsilon \psi \,dr = 0, \qquad \text{ a.e. } \text{ on } {\mathbb {S}^2}. \end{aligned}$$

### Proof

Let us choose and fix $\psi \in L^2(Q_\varepsilon )$. Then by the definitions of the operators involved we have the following equality in $L^2({\mathbb {S}^2})$:

$$\begin{aligned} \int _{1}^{1+\varepsilon }r \widehat{N}_\varepsilon \psi \,dr = \varepsilon M_\varepsilon \widehat{N}_\varepsilon \psi . \end{aligned}$$

Therefore ,we deduce that in order to prove equality (37), it is sufficient to show that

$$\begin{aligned} M_\varepsilon \widehat{N}_\varepsilon = 0. \end{aligned}$$

Hence, by taking into account definitions (36) of $\widehat{N}_\varepsilon$ and (34) of $\widehat{{M}}_\varepsilon$ , we infer that it is sufficient to prove that

$$\begin{aligned} M_\varepsilon = M_\varepsilon \circ {R}_\varepsilon \circ M_\varepsilon . \end{aligned}$$

Let us choose $\psi \in \mathcal {C}(Q_\varepsilon )$ and put $\phi = M_\varepsilon \psi$, i.e.

$$\begin{aligned} \phi (\mathbf{x}) = \frac{1}{\varepsilon }\int _{1}^{1+\varepsilon }r \psi (r \mathbf{x})\,dr. \end{aligned}$$

Note that

$$\begin{aligned} {R}_\varepsilon \phi (\rho \mathbf{x}) = \frac{1}{\rho } \phi (\mathbf{x}), \quad \rho \in (1, 1+ \varepsilon ),\, \mathbf{x}\in {\mathbb {S}^2}. \end{aligned}$$

Thus, we infer that

$$\begin{aligned} M_\varepsilon \left[ {R}_\varepsilon \phi \right] (\mathbf{x})&= \frac{1}{\varepsilon }\int _{1}^{1+\varepsilon }\rho {R}_\varepsilon \phi (\rho \mathbf{x})\,d\rho \\&= \frac{1}{\varepsilon }\int _{1}^{1+\varepsilon }\rho \frac{1}{\rho } \phi (\mathbf{x})\,dr = \phi (\mathbf{x}) = M_\varepsilon \psi (\mathbf{x}), \; \mathbf{x}\in {\mathbb {S}^2}. \end{aligned}$$

Thus, we proved $M_\varepsilon \circ {R}_\varepsilon \circ M_\varepsilon \psi = M_\varepsilon \psi$ for every $\psi \in \mathcal {C}({Q}_\varepsilon )$. Since $\mathcal {C}({Q}_\varepsilon )$ is dense in $L^2(Q_\varepsilon )$ and the maps $M_\varepsilon$ and $M_\varepsilon \circ {R}_\varepsilon \circ M_\varepsilon$ are bounded in $L^2(Q_\varepsilon )$, we conclude that we have proved (37). $\square$

### Lemma 7

For all $\psi , \xi \in L^2(Q_\varepsilon )$, we have

38 $$\begin{aligned} \left( \widehat{{M}}_\varepsilon \psi , \widehat{N}_\varepsilon \xi \right) _{L^2(Q_\varepsilon )} = 0. \end{aligned}$$

### Proof

Let $$\psi , \xi \in L^2(Q_\varepsilon )$$, then

$$\begin{aligned} \left( \widehat{{M}}_\varepsilon \psi , \widehat{N}_\varepsilon \xi \right) _{L^2(Q_\varepsilon )}&= \int _{Q_\varepsilon } \widehat{{M}}_\varepsilon \psi (\mathbf{y}) \cdot \widehat{N}_\varepsilon \xi (\mathbf{y})\,d \mathbf{y}= \int _{\mathbb {S}^2}\int _{1}^{1+\varepsilon }\widehat{{M}}_\varepsilon \psi (r\mathbf{x}) \cdot \widehat{N}_\varepsilon \xi (r\mathbf{x}) r^2\,dr\,d\sigma (\mathbf{x}). \end{aligned}$$

By the definition (34) of the map $\widehat{{M}}_\varepsilon$ and by Lemma 6, we infer that

$$\begin{aligned} \left( \widehat{{M}}_\varepsilon \psi , \widehat{N}_\varepsilon \xi \right) _{L^2(Q_\varepsilon )} = \int _{\mathbb {S}^2}M_\varepsilon \psi (\mathbf{x}) \underbrace{\left( \int _{1}^{1+\varepsilon }r \widehat{N}_\varepsilon \xi (r\mathbf{x})\,dr \right) }_{= 0\,\, \text{ a.e. } \text{ on } {\mathbb {S}^2} \text{ by } \text{ Lemma }~6} \,d\sigma (\mathbf{x}) = 0. \end{aligned}$$

$$\square $$

Next we define projection operators for $\mathbb {R}^3$-valued vector fields using the above maps (for scalar functions), as follows $:$

39 $$\begin{aligned} \widetilde{M}_\varepsilon :{\mathbb {L}^2(Q_\varepsilon )}\ni u = \left( u_r, u_\lambda , u_\varphi \right)&\mapsto \left( 0, \widehat{{M}}_\varepsilon u_\lambda , \widehat{{M}}_\varepsilon u_\varphi \right) \in {\mathbb {L}^2(Q_\varepsilon )},\end{aligned}$$

40 $$\begin{aligned} \widetilde{N}_\varepsilon&= \mathrm{{Id}} - {\widetilde{M}}_{\varepsilon } \in {\mathcal {L}}(\mathbb {L}^2(Q_{\varepsilon })). \end{aligned}$$

### Lemma 8

Let $$u \in {\mathbb {L}^2(Q_\varepsilon )}$$. Then

$$\begin{aligned} \widetilde{M}_\varepsilon u \cdot \mathbf {n} = 0 \qquad \text{ on } \, \partial Q_\varepsilon . \end{aligned}$$

Moreover, if *u* satisfies the boundary condition $u \cdot \mathbf {n} = 0$, then

$$\begin{aligned} \widetilde{N}_\varepsilon u \cdot \mathbf {n} = 0 \qquad \text{ on } \, \partial Q_\varepsilon . \end{aligned}$$

### Proof

The normal vector $\mathbf {n}$ to $\partial Q_\varepsilon$ is given by $\mathbf {n} = \left( 1, 0, 0\right)$. Thus by the definition of $\widetilde{M}_\varepsilon$ we have

$$\begin{aligned} \widetilde{M}_\varepsilon u \cdot \mathbf {n} = 0 \qquad \text{ on } \, \partial Q_\varepsilon . \end{aligned}$$

Now for the second part, from the definition of $\widetilde{N}_\varepsilon$ we have

$$\begin{aligned} \widetilde{N}_\varepsilon u \cdot \mathbf {n} = \underbrace{u \cdot \mathbf {n}}_{=\,0\,\, \text{ from } \text{ b.c. }} - \underbrace{\widetilde{M}_\varepsilon u\cdot \mathbf {n}}_{=\,0\,\, \text{ from } \text{ first } \text{ part }} = 0. \end{aligned}$$

$$\square $$

We also have the following generalisation of Lemma 6.

### Lemma 9

Let $$u \in \mathbb {L}^2(Q_\varepsilon )$$, then

41 $$\begin{aligned} \int _{1}^{1+\varepsilon }r \widetilde{N}_\varepsilon u\,dr = 0, \qquad \text{ a.e. } \text{ on } {\mathbb {S}^2}. \end{aligned}$$

The following Lemma makes sense only for vector fields.

### Lemma 10

Let $$u \in \mathrm { H}_\varepsilon $$, then

$$\begin{aligned} \mathrm{div}\;\widetilde{M}_\varepsilon u = 0 \quad \text{ and } \quad \mathrm{div}\;\widetilde{N}_\varepsilon u = 0 \qquad \text{ in } \, Q_\varepsilon . \end{aligned}$$

### Proof

Let $u \in \mathrm{{U}}_\varepsilon := \left\{ {\mathrm {v}}\in \mathcal {C}({Q_\varepsilon }) :\mathrm{div}\;{\mathrm {v}}= 0\, \text{ in } \, Q_\varepsilon \, \text{ and } \, {\mathrm {v}}\cdot \mathbf {n} = 0\, \text{ on } \partial Q_\varepsilon \right\}$, then using the definition of divergence for a vector field ${\mathrm {v}}= \left( {\mathrm {v}}_r, {\mathrm {v}}_\lambda , {\mathrm {v}}_\varphi \right)$ in spherical coordinates (see (174)), we get for $Q_\varepsilon \ni \mathbf{y}= r\mathbf{x}$, $\mathbf{x}\in {\mathbb {S}^2}$, $r \in (1, 1+\varepsilon )$,

42 $$\begin{aligned}&\mathrm{div}\;\left( (\widetilde{M}_\varepsilon u) (\mathbf{y})\right) = 0 + \frac{1}{r \sin \lambda } \frac{\partial }{\partial \lambda } \left( (\widehat{{M}}_\varepsilon u_\lambda )(\mathbf{y}) \sin \lambda \right) + \frac{1}{r \sin \lambda }\frac{\partial }{\partial \varphi } \left( (\widehat{{M}}_\varepsilon u_\varphi )(\mathbf{y})\right) \nonumber \\&\quad = \frac{1}{r \sin \lambda } \left[ \frac{\partial }{\partial \lambda }\left( \frac{1}{|\mathbf{y}|}(M_\varepsilon u_\lambda )\left( \frac{\mathbf{y}}{|\mathbf{y}|}\right) \sin \lambda \right) + \frac{\partial }{\partial \varphi }\left( \frac{1}{|\mathbf{y}|}(M_\varepsilon u_\varphi )\left( \frac{\mathbf{y}}{|\mathbf{y}|}\right) \right) \right] \nonumber \\&\quad = \frac{1}{r \sin \lambda } \left[ \frac{\partial }{\partial \lambda } \left( \frac{\sin \lambda }{r \varepsilon } \int _{1}^{1+\varepsilon }\rho u_\lambda (\rho , \lambda , \varphi )\,d\rho \right) + \frac{\partial }{\partial \varphi } \left( \frac{1}{r \varepsilon } \int _{1}^{1+\varepsilon }\rho u_\varphi (\rho , \lambda , \varphi )\,d\rho \right) \right] \nonumber \\&\quad =: \frac{1}{r^2 \sin \lambda }\left[ I + II \right] . \end{aligned}$$

Now considering each of the terms individually, we have

43 $$\begin{aligned} II= & {} \frac{1}{ \varepsilon } \frac{\partial }{\partial \varphi } \int _{1}^{1+\varepsilon }\rho u_\varphi \,d\rho = \frac{1}{\varepsilon } \int _{1}^{1+\varepsilon }\rho \frac{\partial u_\varphi }{\partial \varphi }\,d\rho . \end{aligned}$$

44 $$\begin{aligned} I= & {} \frac{1}{\varepsilon } \frac{\partial }{\partial \lambda } \left( \sin \lambda \int _{1}^{1+\varepsilon }\rho u_\lambda \,d\rho \right) = \frac{1}{\varepsilon } \frac{\partial }{\partial \lambda } \int _{1}^{1+\varepsilon }\rho u_\lambda \sin \lambda \,d\rho \nonumber \\= & {} \frac{1}{ \varepsilon } \int _{1}^{1+\varepsilon }\rho \frac{\partial }{\partial \lambda } \left( u_\lambda \sin \lambda \right) \,d\rho . \end{aligned}$$

Using (43) and (44) in the equality (42), we obtain

45 $$\begin{aligned} \mathrm{div}\;\widetilde{M}_\varepsilon u = \frac{1}{r^2 \sin \lambda } \left( \frac{1}{\varepsilon } \int _{1}^{1+\varepsilon }\rho \left[ \frac{\partial }{\partial \lambda } \left( u_\lambda \sin \lambda \right) + \frac{\partial u_\varphi }{\partial \varphi } \right] \,d\rho \right) . \end{aligned}$$

Since $u \in \mathrm { U}_\varepsilon$, $\mathrm{div}\;u = 0$ in $Q_\varepsilon$, which implies

$$\begin{aligned} \frac{1}{\rho ^2} \frac{\partial }{\partial \rho } \left( \rho ^2 u_\rho \right) + \frac{1}{\rho \sin \lambda } \frac{\partial }{\partial \lambda } \left( u_\lambda \sin \lambda \right) + \frac{1}{\rho \sin \lambda } \frac{\partial u_\varphi }{\partial \varphi } = 0. \end{aligned}$$

Using this in (45), we get

$$\begin{aligned} \mathrm{div}\;\widetilde{M}_\varepsilon u&= - \frac{1}{r^2 \sin \lambda } \frac{1}{\varepsilon } \int _{1}^{1+\varepsilon }\rho \frac{\sin \lambda }{\rho } \frac{\partial }{\partial \rho }\left( \rho ^2 u_\rho \right) \,d\rho \\&= - \frac{1}{\varepsilon r^2} \int _{1}^{1+\varepsilon }\frac{\partial }{\partial \rho } \left( \rho ^2 u_\rho \right) \,ds = - \frac{1}{\varepsilon r^2} \rho ^2 u_\rho \bigg |_1^{1+\varepsilon } \\&= - \frac{1}{\varepsilon r^2} \left[ (1+\varepsilon )^2 u_\rho (1+\varepsilon , \cdot , \cdot ) - u_\rho (1, \cdot , \cdot ) \right] = 0 \quad (\text{ since } u\cdot \mathbf {n} = 0). \end{aligned}$$

Thus, we have proved that $\mathrm{div}\;\widetilde{M}_\varepsilon u = 0$, for every $u \in \mathrm { U}_\varepsilon$. Since, $\mathrm { U}_\varepsilon$ is dense in $\mathrm { H}_\varepsilon$, it holds true for every $u \in \mathrm { H}_\varepsilon$ too. The second part follows from the definition of $\widetilde{N}_\varepsilon$ and $\mathrm { H}_\varepsilon$. $\square$

From Lemmas 8 and 10, we infer the following corollary$:$

### Corollary 2

If $u \in \mathrm { H}_\varepsilon$ then $\widetilde{M}_\varepsilon u$ and $\widetilde{N}_\varepsilon u$ belong to $\mathrm { H}_\varepsilon$.

Using the definition of maps $\widetilde{M}_\varepsilon$ and $\widetilde{N}_\varepsilon$ and Lemma 7, we conclude:

### Proposition 1

For all $u, {\mathrm {v}}\in {\mathbb {L}^2(Q_\varepsilon )}$, we have

46 $$\begin{aligned} \left( \widetilde{M}_\varepsilon u , \widetilde{N}_\varepsilon {\mathrm {v}}\right) _{{\mathbb {L}^2(Q_\varepsilon )}} = 0. \end{aligned}$$

Moreover,

47 $$\begin{aligned} \Vert {{u}}\Vert ^2_{{\mathbb {L}^2(Q_\varepsilon )}} = \Vert \widetilde{M}_\varepsilon u\Vert ^2_{{\mathbb {L}^2(Q_\varepsilon )}} + \Vert \widetilde{N}_\varepsilon {{u}}\Vert ^2_{{\mathbb {L}^2(Q_\varepsilon )}}, \qquad {{u}} \in \mathrm { H}_\varepsilon . \end{aligned}$$

Finally we define a projection operator that projects $\mathbb {R}^3$-valued vector fields defined on $Q_\varepsilon$ to the “tangent” vector fields on sphere ${\mathbb {S}^2}$.48

### Lemma 11

Let $$u \in {\mathbb {L}^2(Q_\varepsilon )}$$, then

### Proof

Let $$u \in {\mathbb {L}^2(Q_\varepsilon )}$$, then$$\square $$

### Remark 3

Similar to the scalar case, one can prove that the dual operator  is given by49Indeed, for $u \in {\mathbb {L}^2(Q_\varepsilon )}, {\mathrm {v}}\in {\mathbb {L}^2({\mathbb {S}^2})}$

Using the identities (168)–(170), we can show that for a divergence free smooth vector field ${\mathrm {u}}$

50 $$\begin{aligned} \left( -\varDelta {\mathrm {u}}, {\mathrm {u}}\right) _{{\mathbb {L}^2(Q_\varepsilon )}} = \left( \mathrm{curl}\;{\mathrm {u}}, \mathrm{curl}\;{\mathrm {u}}\right) _{{\mathbb {L}^2(Q_\varepsilon )}} = \Vert \mathrm{curl}\;{\mathrm {u}}\Vert ^2_{{\mathbb {L}^2(Q_\varepsilon )}}. \end{aligned}$$

We define a weighted $L^2$-product on $\mathrm { H}_\varepsilon$ by

51 $$\begin{aligned} \left( u, {\mathrm {v}}\right) _r = \int _{Q_\varepsilon } r^2\,u\cdot {\mathrm {v}}\,d\mathbf{y}, \qquad u, {\mathrm {v}}\in \mathrm { H}_\varepsilon , \end{aligned}$$

and the corresponding norm will be denoted by $\Vert \cdot \Vert _r$ which is equivalent to $\Vert \cdot \Vert _{{\mathbb {L}^2(Q_\varepsilon )}}$, uniformly for $\varepsilon \in (0, \frac{1}{2})$ $:$

52 $$\begin{aligned} \Vert u\Vert ^2_{{\mathbb {L}^2(Q_\varepsilon )}} \le \Vert u\Vert _{r}^2 \le \frac{9}{4}\Vert u\Vert ^2_{{\mathbb {L}^2(Q_\varepsilon )}}, \qquad u \in {\mathbb {L}^2(Q_\varepsilon )}. \end{aligned}$$

We end this section by recalling a lemma and some Poincaré type inequalities from [53].

### Lemma 12

[53, Lemma 1.2] For $u, {\mathrm {v}}\in \mathrm { V}_\varepsilon$, we have

$$\begin{aligned} \left( \mathrm{curl}\;\widetilde{M}_\varepsilon u, \mathrm{curl}\;\widetilde{N}_\varepsilon {\mathrm {v}}\right) _r = 0, \qquad u, {\mathrm {v}}\in \mathrm { V}_\varepsilon . \end{aligned}$$

Moreover,

53 $$\begin{aligned} \Vert \mathrm{curl}\;u\Vert ^2_r = \Vert \mathrm{curl}\;\widetilde{M}_\varepsilon u\Vert ^2_{r} + \Vert \mathrm{curl}\;\widetilde{N}_\varepsilon u\Vert ^2_r,\qquad u \in \mathrm { V}_\varepsilon . \end{aligned}$$

### Corollary 3

Let $\varepsilon \in (0, \frac{1}{2})$ and $u \in \mathrm { V}_\varepsilon$. Then

$$\begin{aligned} \Vert \mathrm{curl}\;\widetilde{M}_\varepsilon u \Vert _{{\mathbb {L}^2(Q_\varepsilon )}}&\le \frac{9}{4} \Vert \mathrm{curl}\;u \Vert ^2_{\mathbb {L}^2(Q_\varepsilon )},\\ \Vert \mathrm{curl}\;\widetilde{N}_\varepsilon u\Vert _{\mathbb {L}^2(Q_\varepsilon )}&\le \frac{9}{4} \Vert \mathrm{curl}\;u\Vert ^2_{\mathbb {L}^2(Q_\varepsilon )}. \end{aligned}$$

### Proof

Let $\varepsilon \in (0, \frac{1}{2})$ and $u \in \mathrm { V}_\varepsilon$. Then, by relation (50), equivalence of norms (52) and Eq. (53), we have

$$\begin{aligned} \Vert \mathrm{curl}\;u\Vert ^2_{\mathbb {L}^2(Q_\varepsilon )}\ge \frac{4}{9} \Vert \mathrm{curl}\;u\Vert ^2_r \ge \frac{4}{9} \Vert \mathrm{curl}\;\widetilde{M}_\varepsilon u\Vert ^2_r \ge \frac{4}{9} \Vert \mathrm{curl}\;\widetilde{M}_\varepsilon u\Vert ^2_{\mathbb {L}^2(Q_\varepsilon )}. \end{aligned}$$

The second inequality can be proved similarly. $\square$

The following two lemmas are taken from [53]. For the sake of completeness and convenience of the reader we have provided the proof in Appendix C.

### Lemma 13

(Poincaré inequality in thin spherical shells) [53, Lemma 2.1] For $0 < \varepsilon \le \tfrac{1}{2}$, we have

54 $$\begin{aligned} \Vert \widetilde{N}_\varepsilon u\Vert _{{\mathbb {L}^2(Q_\varepsilon )}} \le 2 \varepsilon \Vert \mathrm{curl}\;\widetilde{N}_\varepsilon u\Vert _{{\mathbb {L}^2(Q_\varepsilon )}},\qquad \forall \, u\, \in \, \mathrm { V}_\varepsilon . \end{aligned}$$

### Lemma 14

(Ladyzhenskaya’s inequality) [53, Lemma 2.3] There exists a constant $c_1$, independent of $\varepsilon$, such that

55 $$\begin{aligned} \Vert \widetilde{N}_\varepsilon u\Vert _{\mathbb {L}^6(Q_\varepsilon )} \le c_1 \Vert \widetilde{N}_\varepsilon u\Vert _{\mathrm { V}_\varepsilon } ,\qquad \forall \, u\, \in \, \mathrm { V}_\varepsilon . \end{aligned}$$

### Corollary 4

For $\varepsilon \in (0, \frac{1}{2})$, there exists a constant $c_2 > 0$ such that

56 $$\begin{aligned} \Vert \widetilde{N}_\varepsilon u\Vert ^2_{\mathbb {L}^3(Q_\varepsilon )} \le c_2 \varepsilon \Vert \widetilde{N}_\varepsilon u\Vert ^2_{\mathrm { V}_\varepsilon } ,\qquad \forall \, u\, \in \, \mathrm { V}_\varepsilon . \end{aligned}$$

### Proof

Let $u \in \mathrm { V}_\varepsilon$, then by the Hölder inequality, we have

$$\begin{aligned} \Vert \widetilde{N}_\varepsilon u\Vert ^3_{\mathbb {L}^3(Q_\varepsilon )}&= \int _{Q_\varepsilon }|\widetilde{N}_\varepsilon u(\mathbf{y})|^3\,d\mathbf{y}= \int _{Q_\varepsilon }|\widetilde{N}_\varepsilon u(\mathbf{y})|^{3/2}|\widetilde{N}_\varepsilon u(\mathbf{y})|^{3/2}\,d\mathbf{y}\\&\le \left( \int _{Q_\varepsilon }|\widetilde{N}_\varepsilon u(\mathbf{y})|^6\,d\mathbf{y}\right) ^{1/4}\left( \int _{Q_\varepsilon }|\widetilde{N}_\varepsilon u(\mathbf{y})|^2\,d\mathbf{y}\right) ^{3/4} \\&= \Vert \widetilde{N}_\varepsilon u\Vert ^{3/2}_{\mathbb {L}^6(Q_\varepsilon )}\Vert \widetilde{N}_\varepsilon u\Vert ^{3/2}_{\mathbb {L}^2(Q_\varepsilon )}. \end{aligned}$$

Thus, by Lemmas 13 and 14, we get

$$\begin{aligned} \Vert \widetilde{N}_\varepsilon u\Vert ^2_{\mathbb {L}^3(Q_\varepsilon )} \le c_1\Vert \widetilde{N}_\varepsilon u\Vert _{\mathrm { V}_\varepsilon } 2\varepsilon \Vert \widetilde{N}_\varepsilon u\Vert _{\mathrm { V}_\varepsilon } = c_2 \varepsilon \Vert \widetilde{N}_\varepsilon u\Vert _{\mathrm { V}_\varepsilon }^2. \end{aligned}$$

$$\square $$

In the following lemma we enlist some properties of operators $\widehat{{M}}_\varepsilon$, $\widehat{N}_\varepsilon$, $\widetilde{M}_\varepsilon$ and $\widetilde{N}_\varepsilon$.

### Lemma 15

Let $$\varepsilon > 0$$. Then

(i) for $$\psi \in L^2(Q_\varepsilon )$$
  57 $$\begin{aligned}&\widehat{{M}}_\varepsilon \left( \widehat{{M}}_\varepsilon \psi \right) = \widehat{{M}}_\varepsilon \psi , \end{aligned}$$
  58 $$\begin{aligned}&\widehat{N}_\varepsilon \left( \widehat{N}_\varepsilon \psi \right) = \widehat{N}_\varepsilon \psi , \end{aligned}$$
  59 $$\begin{aligned}&\widehat{{M}}_\varepsilon \left( \widehat{N}_\varepsilon \psi \right) = 0, \qquad \text{ and } \qquad \widehat{N}_\varepsilon \left( \widehat{{M}}_\varepsilon \psi \right) = 0, \end{aligned}$$
(ii) and for $u \in {\mathbb {L}^2(Q_\varepsilon )}$
  60 $$\begin{aligned}&\widetilde{M}_\varepsilon \left( \widetilde{M}_\varepsilon u\right) = \widetilde{M}_\varepsilon u, \end{aligned}$$
  61 $$\begin{aligned}&\widetilde{N}_\varepsilon \left( \widetilde{N}_\varepsilon u \right) = \widetilde{N}_\varepsilon u, \end{aligned}$$
  62 $$\begin{aligned}&\widetilde{M}_\varepsilon \left( \widetilde{N}_\varepsilon u\right) = 0, \qquad \text{ and } \qquad \widetilde{N}_\varepsilon \left( \widetilde{M}_\varepsilon u\right) = 0. \end{aligned}$$

### Proof

Let $$\psi \in \mathcal {C}({Q}_\varepsilon )$$. Put

$$\begin{aligned} \phi = \widehat{{M}}_\varepsilon \psi , \end{aligned}$$

i.e. for $$\mathbf{y}\in Q_\varepsilon $$

$$\begin{aligned} \phi (\mathbf{y})&= \frac{1}{|\mathbf{y}|} \left( M_\varepsilon \psi \right) \left( \frac{\mathbf{y}}{|\mathbf{y}|}\right) \\&= \frac{1}{|\mathbf{y}|} \frac{1}{\varepsilon } \int _{1}^{1+\varepsilon }r \psi \left( r \frac{\mathbf{y}}{|\mathbf{y}|}\right) \,dr. \end{aligned}$$

Next for $$\mathbf{z}\in Q_\varepsilon $$

$$\begin{aligned} \vert \mathbf{z}\vert \left( \widehat{{M}}_\varepsilon \phi \right) (\mathbf{z})&= \left( M_\varepsilon \phi \right) \left( \frac{\mathbf{z}}{|\mathbf{z}|}\right) = \frac{1}{\varepsilon } \int _{1}^{1+\varepsilon }\rho \phi \left( \rho \frac{\mathbf{z}}{|\mathbf{z}|}\right) \,d\rho \\&= \frac{1}{\varepsilon ^2} \int _{1}^{1+\varepsilon }\rho \frac{1}{\left| \rho \frac{\mathbf{z}}{|\mathbf{z}|}\right| } \left[ \int _{1}^{1+\varepsilon }r \psi \left( r \frac{\rho \frac{\mathbf{z}}{|\mathbf{z}|}}{\left| \rho \frac{\mathbf{z}}{|\mathbf{z}|}\right| } \right) \,dr\right] \,d\rho \\&= \frac{1}{\varepsilon ^2} \int _{1}^{1+\varepsilon }\left[ \int _{1}^{1+\varepsilon }r \psi \left( r \frac{\mathbf{z}}{|\mathbf{z}|}\right) \,dr \right] \,d\rho \\&= \frac{1}{\varepsilon } \int _{1}^{1+\varepsilon }r \psi \left( r \frac{\mathbf{z}}{|\mathbf{z}|} \right) \,dr = \left( M_\varepsilon \psi \right) \left( \frac{\mathbf{z}}{|\mathbf{z}|}\right) \\&= \vert \mathbf{z}\vert \left( \widehat{{M}}_\varepsilon \psi \right) (\mathbf{z}). \end{aligned}$$

Hence, we proved (57) for every $\psi \in \mathcal {C}({Q}_\varepsilon )$. Since $\mathcal {C}({Q}_\varepsilon )$ is dense in $L^2(Q_\varepsilon )$, it holds true for every $\psi \in L^2(Q_\varepsilon )$.

**Proof of first part of** (59). Let $\psi \in \mathcal {C}({Q}_\varepsilon )$. Put $\phi = \widehat{N}_\varepsilon \psi \in \mathcal {C}({Q}_\varepsilon )$. By Lemma 6

$$\begin{aligned} \int _{1}^{1+\varepsilon }r \phi (\mathbf{y})\,dr = 0, \qquad \mathbf{y}\in Q_\varepsilon . \end{aligned}$$

Therefore, for $$\mathbf{y}\in Q_\varepsilon $$,

$$\begin{aligned} \left( M_\varepsilon \phi \right) \left( \frac{\mathbf{y}}{|\mathbf{y}|}\right) = \frac{1}{\varepsilon }\int _{1}^{1+\varepsilon }r \phi (\mathbf{y})\,dr = 0. \end{aligned}$$

Therefore, we infer that

$$\begin{aligned} \left( \widehat{{M}}_\varepsilon \phi \right) (\mathbf{y}) = \frac{1}{|\mathbf{y}|}\left( M_\varepsilon \phi \right) \left( \frac{\mathbf{y}}{|\mathbf{y}|}\right) = 0, \end{aligned}$$

for all $\mathbf{y}\in Q_\varepsilon$. Thus, we have established first part of (59) for all $\psi \in \mathcal {C}({Q}_\varepsilon )$. Using the density argument, we can prove it for all $\psi \in L^2(Q_\varepsilon )$.

Now for (58), by the definition of $\widehat{N}_\varepsilon$ and (59), we obtain

$$\begin{aligned} \widehat{N}_\varepsilon \left( \widehat{N}_\varepsilon \psi \right) = \widehat{N}_\varepsilon \psi - \widehat{{M}}_\varepsilon \left( \widehat{N}_\varepsilon \psi \right) = \widehat{N}_\varepsilon \psi . \end{aligned}$$

Again using the definition of $\widehat{N}_\varepsilon$ and Eq.(57), we have

$$\begin{aligned} \widehat{N}_\varepsilon \left( \widehat{{M}}_\varepsilon \psi \right) = \widehat{{M}}_\varepsilon \psi - \widehat{{M}}_\varepsilon \left( \widehat{{M}}_\varepsilon \psi \right) = \widehat{{M}}_\varepsilon \psi - \widehat{{M}}_\varepsilon \psi =0, \end{aligned}$$

concluding the proof of second part of (59).

**Proof of** (60). Let $u \in \mathcal {C}({Q}_\varepsilon , \mathbb {R}^3)$. Write $u = \left( u_r, u_\lambda , u_\varphi \right)$. Put ${\mathrm {v}}= \widetilde{M}_\varepsilon u$, i.e.

$$\begin{aligned}{\mathrm {v}}= \left( 0, {\mathrm {v}}_\lambda , {\mathrm {v}}_\varphi \right) ,\end{aligned}$$

where

$$\begin{aligned}{\mathrm {v}}_\lambda = \widehat{{M}}_\varepsilon u_\lambda ,\quad \text{ and } \quad {\mathrm {v}}_\varphi = \widehat{{M}}_\varepsilon u_\varphi .\end{aligned}$$

Thus, by the definition of $\widetilde{M}_\varepsilon$ and identity (57)

$$\begin{aligned} \widetilde{M}_\varepsilon \left( \widetilde{M}_\varepsilon u \right)&= \widetilde{M}_\varepsilon {\mathrm {v}}= \left( 0, \widehat{{M}}_\varepsilon {\mathrm {v}}_\lambda , \widehat{{M}}_\varepsilon {\mathrm {v}}_\varphi \right) \\&= \left( 0, \widehat{{M}}_\varepsilon \left( \widehat{{M}}_\varepsilon u_\lambda \right) , \widehat{{M}}_\varepsilon \left( \widehat{{M}}_\varepsilon u_\varphi \right) \right) \\&= \left( 0, \widehat{{M}}_\varepsilon u_\lambda , \widehat{{M}}_\varepsilon u_\varphi \right) = {\mathrm {v}}= \widetilde{M}_\varepsilon u . \end{aligned}$$

We can extend this to $u \in {\mathbb {L}^2(Q_\varepsilon )}$ by the density argument. The remaining identities can be also established similarly as in the case of scalar functions. $\square$

Later in the proof of Theorem 3, in order to pass to the limit we will use an operator  defined by63where

$$\begin{aligned} {\mathbb {L}^2({\mathbb {S}^2})}\ni u = \left( 0, u_\lambda (\mathbf{x}), u_\varphi (\mathbf{x})\right) , \qquad \mathbf{x}\in {\mathbb {S}^2}. \end{aligned}$$

Using the definition of map ${R}_\varepsilon$ from Lemma 2, we can rewrite  as64Note that  is a bounded linear map from ${\mathbb {L}^2({\mathbb {S}^2})}$ to ${\mathbb {L}^2(Q_\varepsilon )}$.

This operator  is retract of , i.e. a map  such that65One can easily show that if $u \in \mathrm {D}(\mathrm { A})$ then . In particular, for $u \in \mathrm { H}$, . Next we establish certain scaling properties for the map .

### Lemma 16

Let $$\varepsilon > 0$$, then66

### Proof

Let $\varepsilon > 0$ and consider ${\mathbb {L}^2({\mathbb {S}^2})}\ni u = \left( 0, u_\lambda , u_\varphi \right)$. Then, by the definition of the retract operator  and ${\mathbb {L}^2(Q_\varepsilon )}$-norm we have$\square$

Using the definition of the map  and Lemmas 3, 4 we can deduce the following two lemmas (we provide the detailed proof of the latter in Appendix C):

### Lemma 17

Let $u \in \mathbb {W}^{1,p}({\mathbb {S}^2})$ for $p \ge 2$. Then for $\varepsilon \in (0,1)$ there exists a constant $C > 0$ independent of $\varepsilon$ such that67

### Lemma 18

Let $u \in \mathbb {H}^2({\mathbb {S}^2}) \cap \mathrm { H}$ and $\varepsilon \in (0,1)$. Then68where $\varvec{{\Delta }}^\prime \,$ is defined in (180).

## Stochastic NSE on Thin Spherical Domains

This section deals with the proof of our main result, Theorem 3. First we introduce our two systems; stochastic NSE in thin spherical domain and stochastic NSE on the sphere, then we present the definition of martingale solutions for both systems. We also state the assumptions under which we prove our result. In Sect. 4.1, we obtain a priori estimates (formally) which we further use to establish some tightness criterion (see Sect. 4.2) which along with Jakubowski’s generalisation of Skorokhod Theorem gives us a converging (in $\varepsilon$) subsequence. At the end of this section we show that the limiting object of the previously obtained converging subsequence is a martingale solution of stochastic NSE on the sphere (see Sect. 4.3).

In thin spherical domain $Q_\varepsilon$, which was introduced in (3), we consider the following stochastic Navier–Stokes equations (SNSE)

69 $$\begin{aligned} d \widetilde{u}_\varepsilon - [ \nu \varDelta \widetilde{u}_\varepsilon - (\widetilde{u}_\varepsilon \cdot \nabla ) \widetilde{u}_\varepsilon - \nabla \widetilde{p}_\varepsilon ]dt = \widetilde{f}_\varepsilon dt + \widetilde{G}_\varepsilon \, d\widetilde{W}_\varepsilon (t)&\quad \text { in } Q_\varepsilon \times (0,T), \end{aligned}$$

70 $$\begin{aligned} \mathrm{div}\;\widetilde{u}_\varepsilon = 0&\quad \text { in } Q_\varepsilon \times (0,T), \end{aligned}$$

71 $$\begin{aligned} \widetilde{u}_\varepsilon \cdot \mathbf {n} = 0, \quad \mathrm{curl}\;\widetilde{u}_\varepsilon \times \mathbf {n} = 0&\quad \text { on } \partial Q_\varepsilon \times (0,T), \end{aligned}$$

72 $$\begin{aligned} \widetilde{u}_\varepsilon (0, \cdot ) = \widetilde{u}_0^\varepsilon&\quad \text { in } Q_\varepsilon . \end{aligned}$$

Recall that, $\widetilde{u}_\varepsilon =(\widetilde{u}_\varepsilon ^r, \widetilde{u}_\varepsilon ^\lambda , \widetilde{u}_\varepsilon ^\varphi )$ is the fluid velocity field, *p* is the pressure, $\nu >0$ is a (fixed) kinematic viscosity, $\widetilde{u}_0^\varepsilon$ is a divergence free vector field on $Q_\varepsilon$ and $\mathbf {n}$ is the unit normal outer vector to the boundary $\partial Q_\varepsilon$. We assume that^1^$N \in \mathbb {N}$. We consider a family of maps

$$\begin{aligned} \widetilde{G}_\varepsilon : \mathbb {R}_+\rightarrow \mathcal {T}_2(\mathbb {R}^N; \mathrm { H}_\varepsilon ) \end{aligned}$$

such that

73 $$\begin{aligned} \widetilde{G}_\varepsilon (t) k := \sum _{j=1}^N \widetilde{g}^j_\varepsilon (t) k_j,\;\; k=(k_j)_{j=1}^N \in \mathbb {R}^N, \end{aligned}$$

for some $\widetilde{g}_\varepsilon ^j: \mathbb {R}_+\rightarrow \mathrm { H}_\varepsilon$, $j=1, \ldots ,N$. The Hilbert–Schmidt norm of $\widetilde{G}_\varepsilon$ is given by

74 $$\begin{aligned} \Vert \widetilde{G}_\varepsilon (s)\Vert ^2_{\mathcal {T}_2(\mathbb {R}^N; \mathrm { H}_\varepsilon )} = \sum _{j=1}^N \Vert \widetilde{g}^j_\varepsilon (s)\Vert ^2_{{\mathbb {L}^2(Q_\varepsilon )}}. \end{aligned}$$

Finally we assume that $\widetilde{W}_\varepsilon (t)$, $t \ge 0$ is an $\mathbb {R}^N$-valued Wiener process defined on the probability space $\left( \varOmega , \mathcal {F}, \mathbb {F}, \mathbb {P}\right)$. We assume that $\left( \beta _j\right) _{j=1}^N$ are i.i.d real valued Brownian motions such that $W(t)=\bigl (\beta _j(t)\bigr )_{j=1}^N$, $t\ge 0$.

In this section, we shall establish convergence of the radial averages of the martingale solution of the 3D stochastic equations (69)–(72), as the thickness of the shell $\varepsilon \rightarrow 0$, to a martingale solution *u* of the following stochastic Navier–Stokes equations on the sphere ${\mathbb {S}^2}$:

75 $$\begin{aligned} du - \left[ \nu \varvec{{\Delta }}^\prime \, u - (u \cdot \nabla ^\prime ) u - \nabla ^\prime p \right] dt = f dt + G\,dW&\quad \text { in } {\mathbb {S}^2}\times (0,T), \end{aligned}$$

76 $$\begin{aligned} \mathrm{{div}^\prime }u = 0&\quad \text { in } {\mathbb {S}^2}\times (0,T), \end{aligned}$$

77 $$\begin{aligned} u(0, \cdot ) = u_0&\quad \text { in } {\mathbb {S}^2}, \end{aligned}$$

where $u=(0, u_\lambda , u_\varphi )$ and $\varvec{{\Delta }}^\prime \,$, $\nabla ^\prime$ are as defined in (176)–(180). Assumptions on initial data and external forcing will be specified later (see Assumptions 1, 2). Here, $G : \mathbb {R}_+\rightarrow \mathcal {T}_2(\mathbb {R}^N; \mathrm { H})$ and *W*(*t*), $t \ge 0$ is an $\mathbb {R}^N$-valued Wiener process such that

78 $$\begin{aligned} G(t)dW(t) := \sum _{j=1}^N g^j(t) d\beta _j(t), \end{aligned}$$

where $N \in \mathbb {N}$, $\left( \beta _j\right) _{j=1}^N$ are i.i.d real valued Brownian motions as before and $\left\{ g^j\right\} _{j=1}^N$ are elements of $\mathrm { H}$, with certain relation to $\widetilde{g}_\varepsilon ^j$, which is specified later in Assumption 2.

### Remark 4

We are aware of other formulations of the Laplacian in (75) such as the one with an additional Ricci tensor term [47, 48]. However, as it was written in [47, p. 144], “Deriving appropriate equations of motion involves dynamical considerations which do not seem adapted to Riemannian space; in particular it is not evident how to formulate the principle of conservation of momentum.” Therefore, in this paper, we follow the approach presented in [53], that the Navier–Stokes equations on the sphere is the thin shell limit of the 3-dimensional Navier–Stokes equations defined on a thin spherical shell.

Now, we specify assumptions on the initial data $\widetilde{u}_0^\varepsilon$ and external forcing $\widetilde{f}_\varepsilon$, $\widetilde{g}_\varepsilon ^j$.

### Assumption 1

Let $\left( \varOmega , \mathcal {F}, \mathbb {F}, \mathbb {P}\right)$ be the given filtered probability space. Let us assume that $p \ge 2$ and that $\widetilde{u}_0^\varepsilon \in \mathrm { H}_\varepsilon$, for $\varepsilon \in (0,1]$, such that for some $C_1 > 0$

79 $$\begin{aligned} \Vert \widetilde{u}_0^\varepsilon \Vert _{{\mathbb {L}^2(Q_\varepsilon )}} = C_1\varepsilon ^{1/2}, \qquad \varepsilon \in (0,1]. \end{aligned}$$

We also assume that $\widetilde{f}_\varepsilon \in L^p([0,T]; \mathrm { V}_\varepsilon ^\prime )$, for $\varepsilon \in (0,1]$, such that for some $C_2 > 0$,

80 $$\begin{aligned} \int _0^T\Vert \widetilde{f}_\varepsilon (s)\Vert ^p_{\mathrm { V}_\varepsilon ^\prime }\,ds \le C_2 \varepsilon ^{p/2}, \qquad \varepsilon \in (0,1]. \end{aligned}$$

Let $\widetilde{W}^\varepsilon$ be an $\mathbb {R}^N$-valued Wiener process as before and assume that

$$\begin{aligned} \widetilde{G}_\varepsilon \in L^p(0,T; \mathcal {T}_2(\mathbb {R}^N; \mathrm { H}_\varepsilon )), \;\;\text{ for } \varepsilon \in (0,1], \end{aligned}$$

such that, using convention (73), for each $j=1,\ldots ,N$,

81 $$\begin{aligned} \int _0^T\Vert \widetilde{g}_\varepsilon ^j(t)\Vert ^p_{{\mathbb {L}^2(Q_\varepsilon )}}dt = O(\varepsilon ^{p/2}),\;\; \varepsilon \in (0,1]. \end{aligned}$$

Projecting the stochastic NSE (on thin spherical shell) (69)–(72) onto $\mathrm { H}_\varepsilon$ using the Leray–Helmholtz projection operator and using the definitions of operators from Sect. 2.1, we obtain the following abstract Itô equation in $\mathrm { H}_\varepsilon$, $t \in [0,T]$

82 $$\begin{aligned} d \widetilde{u}_\varepsilon (t) + \left[ \nu \mathrm { A}_\varepsilon \widetilde{u}_\varepsilon (t) + B_\varepsilon (\widetilde{u}_\varepsilon (t), \widetilde{u}_\varepsilon (t))\right] dt = \widetilde{f}_\varepsilon (t)\,dt + \widetilde{G}_\varepsilon (t)\,d \widetilde{W}_\varepsilon (t), \quad \widetilde{u}_\varepsilon (0) = \widetilde{u}_0^\varepsilon . \end{aligned}$$

### Definition 1

Let $\varepsilon \in (0,1]$. A martingale solution to (82) is a system

$$\begin{aligned} \left( \varOmega , \mathcal {F}, \mathbb {F}, \mathbb {P}, \widetilde{W}_\varepsilon , \widetilde{u}_\varepsilon \right) \end{aligned}$$

where $\left( \varOmega , \mathcal {F}, \mathbb {P}\right)$ is a probability space and $\mathbb {F}= \left( \mathcal {F}_t\right) _{t \ge 0}$ is a filtration on it, such that

- $\widetilde{W}_\varepsilon$ is a $\mathbb {R}^N$-valued Wiener process on $\left( \varOmega , \mathcal {F}, \mathbb {F}, \mathbb {P}\right)$,
- $\widetilde{u}_\varepsilon$ is $\mathrm { V}_\varepsilon$-valued progressively measurable process, $\mathrm { H}_\varepsilon$-valued weakly continuous $\mathbb {F}$-adapted process such that^2^$\mathbb {P}$-a.s.
  $$\begin{aligned}&\widetilde{u}_\varepsilon (\cdot , \omega ) \in \mathcal {C}([0,T],\mathrm{H}_{\varepsilon }^w) \cap L^2(0,T; \mathrm { V}_\varepsilon ),\\&\mathbb {E}\left[ \frac{1}{2} \sup _{0\le s \le T} \Vert \widetilde{u}_\varepsilon (s) \Vert ^2_{{\mathbb {L}^2(Q_\varepsilon )}} + \nu \int _0^T \Vert \mathrm{curl}\;\widetilde{u}_\varepsilon (s) \Vert ^2_{{\mathbb {L}^2(Q_\varepsilon )}}\,ds \right] < \infty \end{aligned}$$
  and, for all $t \in [0,T]$ and ${\mathrm {v}}\in \mathrm { V}_\varepsilon$, $\mathbb {P}$-a.s.,
  83 $$\begin{aligned}&(\widetilde{u}_\varepsilon (t),{\mathrm {v}})_{{\mathbb {L}^2(Q_\varepsilon )}} + \nu \int _0^t (\mathrm{curl}\;\widetilde{u}_\varepsilon (s), \mathrm{curl}\;{\mathrm {v}})_{{\mathbb {L}^2(Q_\varepsilon )}} \,ds + \int _0^t \left\langle B_\varepsilon ( \widetilde{u}_\varepsilon (s), \widetilde{u}_\varepsilon (s)), {\mathrm {v}}\right\rangle _\varepsilon \,ds \nonumber \\&\quad = (\widetilde{u}_0^\varepsilon , {\mathrm {v}})_{{\mathbb {L}^2(Q_\varepsilon )}} + \int _0^t \left\langle \widetilde{f}_\varepsilon (s),{\mathrm {v}}\right\rangle _{\varepsilon }\,ds + \left( \int _0^t \widetilde{G}_\varepsilon (s)\,d\widetilde{W}_\varepsilon (s),{\mathrm {v}}\right) _{\mathbb {L}^2(Q_\varepsilon )}. \end{aligned}$$

In the following remark we show that a martingale solution $\widetilde{u}_\varepsilon$ of (82), as defined above, satisfies an equivalent equation in the weak form.

### Remark 5

Let $\widetilde{u}_\varepsilon = \widetilde{u}_\varepsilon (t),$ $t \ge 0$ be a martingale solution of (82). We will use the following notations84and also from Lemma 15 we have

$$\begin{aligned} \widetilde{u}_\varepsilon (t) = \widetilde{\alpha }_\varepsilon (t) + \widetilde{\beta }_\varepsilon (t), \qquad t \in [0,T]. \end{aligned}$$

Then, for $\phi \in \mathrm {D}(\mathrm { A})$, we haveand using Lemma 6, Proposition 1 and Lemma 12, we can rewrite the weak formulation identity (83) as follows.85where $\langle \cdot , \cdot \rangle$ denotes the duality between $\mathrm { V}^\prime$ and $\mathrm { V}$.

Next, we present the definition of a martingale solution for stochastic NSE on ${\mathbb {S}^2}$.

### Definition 2

A martingale solution to equation (75)–(77) is a system

$$\begin{aligned} \left( \widehat{\varOmega }, \widehat{\mathcal {F}}, \widehat{\mathbb {F}}, \widehat{\mathbb {P}}, \widehat{W}, \widehat{u}\right) \end{aligned}$$

where $\left( \widehat{\varOmega }, \widehat{\mathcal {F}},\widehat{\mathbb {P}} \right)$ is a probability space and $\widehat{\mathbb {F}} = \left( \widehat{\mathcal {F}}_t\right) _{t \ge 0}$ is a filtration on it, such that

- $\widehat{W}$ is an $\mathbb {R}^N$-valued Wiener process on $\left( \widehat{\varOmega }, \widehat{\mathcal {F}}, \widehat{\mathbb {F}}, \widehat{\mathbb {P}}\right)$,
- $\widehat{u}$ is $\mathrm { V}$-valued progressively measurable process, $\mathrm { H}$-valued continuous $\widehat{\mathbb {F}}$-adapted process such that
  $$\begin{aligned}&\widehat{u}(\cdot , \omega ) \in \mathcal {C}([0,T],\mathrm{H}) \cap L^2(0,T; \mathrm { V}),\\&\quad \widehat{\mathbb {E}} \left[ \sup _{0\le s \le T} \Vert \widehat{u}(s) \Vert ^2_{{\mathbb {L}^2({\mathbb {S}^2})}} + \nu \int _0^T \Vert \mathrm{{curl}^\prime }\widehat{u}(s) \Vert ^2_{{\mathbb {L}^2({\mathbb {S}^2})}}\,ds \right] < \infty \end{aligned}$$
  and
  86 $$\begin{aligned}&\left( \widehat{u}(t), \phi \right) _{\mathbb {L}^2({\mathbb {S}^2})}+ \nu \int _0^t \left( \mathrm{{curl}^\prime }\widehat{u}(s), \mathrm{{curl}^\prime }\phi \right) _{\mathbb {L}^2({\mathbb {S}^2})}\,ds + \int _0^{t} \left( \left[ \widehat{u}(s) \cdot \nabla ^\prime \right] \widehat{u}(s), \phi \right) _{\mathbb {L}^2({\mathbb {S}^2})}\,ds \nonumber \\&\quad = \left( u_0, \phi \right) _{\mathbb {L}^2({\mathbb {S}^2})}+ \int _0^{t} \left( f(s), \phi \right) _{\mathbb {L}^2({\mathbb {S}^2})}\,ds + \left( \int _0^t G(s) d \widehat{W}(s), \phi \right) _{\mathbb {L}^2({\mathbb {S}^2})}, \end{aligned}$$
  for all $t \in [0,T]$ and $\phi \in \mathrm { V}$.

### Assumption 2

Let $p \ge 2$. Let $\left( \widehat{\varOmega }, \widehat{\mathcal {F}}, \widehat{\mathbb {F}}, \widehat{\mathbb {P}}\right)$ be the given probability space, ${\mathrm {u}}_0 \in \mathrm { H}$ such that87Let $f \in L^p([0,T]; \mathrm { V}^\prime )$, such that for every $s \in [0,T]$,88And finally, we assume that $G \in L^p(0,T; \mathcal {T}_2(\mathbb {R}^N; \mathrm { H}))$, such that for each $j=1,\ldots ,N$ and $s \in [0,T]$,  converges weakly to $g^j(s)$ in $\mathbb {L}^2({\mathbb {S}^2})$ as $\varepsilon \rightarrow 0$ and

89 $$\begin{aligned} \int _0^T\Vert g^j(t)\Vert ^2_{{\mathbb {L}^2({\mathbb {S}^2})}}dt \le M, \end{aligned}$$

for some $$M > 0$$.

### Remark 6

(*Existence of martingale solutions*) In a companion paper [7] we will address an easier question about the existence of a martingale solution for (1)–(5) in a more general setting with multiplicative noise. The key idea of the proof is taken from [11], where authors prove existence of a martingale solution for stochastic NSE in unbounded 3D domains.

The existence of a pathwise unique strong solution (hence a martingale solution) for the stochastic NSE on a sphere ${\mathbb {S}^2}$ is already established by two of the authors and Goldys in [9]. Through this article we give another proof of the existence of a martingale solution for such a system.

We end this subsection by stating the main theorem of this article.

### Theorem 3

Let the given data $\widetilde{u}_0^\varepsilon$, $u_0$, $\widetilde{f}_\varepsilon$, *f*, $\widetilde{g}_\varepsilon ^j$, $g^j$, $j \in \{1, \ldots , N\}$ satisfy Assumptions 1 and 2. Let $\left( \varOmega , \mathcal {F}, \mathbb {F}, \mathbb {P}, \widetilde{W}_\varepsilon , \widetilde{u}_\varepsilon \right)$ be a martingale solution of (69)–(72) as defined in Definition 1. Then, the averages in the radial direction of this martingale solution i.e.  converge to a martingale solution, $\left( \widehat{\varOmega }, \widehat{\mathcal {F}}, \widehat{\mathbb {F}}, \widehat{\mathbb {P}}, \widehat{W}, \widehat{u}\right)$, of (75)–(77) in $L^2(\widehat{\varOmega }\times [0,T]\times {\mathbb {S}^2})$.

### Remark 7

According to Remark 6, for every $\varepsilon \in [0,1]$ there exists a martingale solution of (69)–(72) as defined in Definition 1, i.e. we will obtain a tuple $\left( \varOmega _\varepsilon , \mathcal {F}_\varepsilon , \mathbb {F}_\varepsilon , \mathbb {P}_\varepsilon , \widetilde{W}_\varepsilon , \widetilde{u}_\varepsilon \right)$ as a martingale solution. It was shown in [31] that is enough to consider only one probability space, namely,

$$\begin{aligned} \left( \varOmega _\varepsilon , \mathcal {F}_\varepsilon , \mathbb {P}_\varepsilon \right) = \left( [0,1], \mathcal {B}([0,1]), \mathcal {L}\right) \qquad \forall \, \varepsilon \in (0,1], \end{aligned}$$

where $\mathcal {L}$ denotes the Lebesgue measure on [0, 1]. Thus, it is justified to consider the probability space $\left( \varOmega , \mathcal {F}, \mathbb {P}\right)$ independent of $\varepsilon$ in Theorem 3.

### Estimates

From this point onward we will assume that for every $\varepsilon \in (0,1]$ there exists a martingale solution $\left( \varOmega , \mathcal {F}, \mathbb {F}, \mathbb {P}, \widetilde{W}_\varepsilon , \widetilde{u}_\varepsilon \right)$ of (82). Please note that we do not claim neither we use the uniqueness of this solution.

The main aim of this subsection is to obtain estimates for $\alpha _\varepsilon$ and $\widetilde{\beta }_\varepsilon$ uniform in $\varepsilon$ using the estimates for the process $\widetilde{u}_\varepsilon$.

The energy inequality (90) and the higher-order estimates (105)–(106), satisfied by the process $\widetilde{u}_\varepsilon$, as obtained in Lemmas 19 and 22 is actually a consequence (essential by-product) of the existence proof. In principle, one obtains these estimates (uniform in the approximation parameter *N*) for the finite-dimensional process $\widetilde{u}_\varepsilon ^{(N)}$ (using Galerkin approximation) with the help of the Itô lemma. Then, using the lower semi-continuity of norms, convergence result ($\widetilde{u}_\varepsilon ^{(N)} \rightarrow \widetilde{u}_\varepsilon$ in some sense), one can establish the estimates for the limiting process. Such a methodology was employed in a proof of Theorem 4.8 in the recent paper [13] by the first named author, Motyl and Ondreját.

In Lemmas 19 and 22 we present a formal proof where we assume that one can apply (ignoring the existence of Lebesgue and stochastic integrals) the Itô lemma to the infinite dimensional process $\widetilde{u}_\varepsilon$. The idea is to showcase (though standard) the techniques involved in establishing such estimates.

#### Lemma 19

Let $\widetilde{u}_0^\varepsilon \in \mathrm { H}_\varepsilon$, $\widetilde{f}_\varepsilon \in \mathbb {L}^2(\varOmega \times [0,T]; \mathrm { V}^\prime _\varepsilon )$ and $\widetilde{G}_\varepsilon \in \mathbb {L}^2(\varOmega \times [0,T]; \mathcal {T}_2(\mathbb {R}^N; \mathrm { H}_\varepsilon ))$. Then, the martingale solution $\widetilde{u}_\varepsilon$ of (82) satisfies the following energy inequality

90 $$\begin{aligned} \begin{aligned}&\mathbb {E}\left[ \frac{1}{2} \sup _{0\le s \le T} \Vert \widetilde{u}_\varepsilon (s)\Vert ^2_{{\mathbb {L}^2(Q_\varepsilon )}} + \nu \int _0^T \Vert \mathrm{curl}\;\widetilde{u}_\varepsilon (s) \Vert ^2_{{\mathbb {L}^2(Q_\varepsilon )}}\,ds \right] \\&\quad \le \Vert \widetilde{u}^\varepsilon _0\Vert ^2_{{\mathbb {L}^2(Q_\varepsilon )}} + \frac{1}{\nu } \int _0^T \Vert \widetilde{f}_\varepsilon (s)\Vert ^2_{\mathrm { V}^\prime _\varepsilon }\,ds + K \int _0^T \Vert \widetilde{G}_\varepsilon (s)\Vert _{\mathcal {T}_2(\mathbb {R}^N; \mathrm { H}_\varepsilon )}^2\,ds, \end{aligned} \end{aligned}$$

where *K* is some positive constant independent of $\varepsilon$.

#### Proof

Using the Itô formula for the function $\Vert \xi \Vert ^2_{{\mathbb {L}^2(Q_\varepsilon )}}$ with the process $\widetilde{u}_\varepsilon$, for a fixed $t \in [0,T]$ we have

91 $$\begin{aligned} \begin{aligned}&\Vert \widetilde{u}_\varepsilon (t)\Vert ^2_{{\mathbb {L}^2(Q_\varepsilon )}} + 2\nu \int _0^t \Vert \mathrm{curl}\;\widetilde{u}_\varepsilon (s)\Vert ^2_{{\mathbb {L}^2(Q_\varepsilon )}}\,ds \le \Vert \widetilde{u}_0^\varepsilon \Vert ^2_{{\mathbb {L}^2(Q_\varepsilon )}} + 2\int _0^t \left\langle \widetilde{f}_\varepsilon (s), \widetilde{u}_\varepsilon (s)\right\rangle _\varepsilon \,ds \\&\qquad \qquad \qquad + 2 \int _0^t \left( \widetilde{G}_\varepsilon (s)\,d\widetilde{W}_\varepsilon (s), \widetilde{u}_\varepsilon (s)\right) _{{\mathbb {L}^2(Q_\varepsilon )}} + \int _0^t \Vert \widetilde{G}_\varepsilon (s)\Vert ^2_{\mathcal {T}_2(\mathbb {R}^N; \mathrm { H}_\varepsilon )}\, ds. \end{aligned} \end{aligned}$$

Using the Cauchy–Schwarz inequality and the Young inequality, we get the following estimate

$$\begin{aligned} \left| \left\langle \widetilde{f}_\varepsilon , \widetilde{u}_\varepsilon \right\rangle _\varepsilon \right| \le \Vert \widetilde{u}_\varepsilon \Vert _{\mathrm { V}_\varepsilon } \Vert \widetilde{f}_\varepsilon \Vert _{\mathrm { V}^\prime _\varepsilon } \le \frac{\nu }{2} \Vert \widetilde{u}_\varepsilon \Vert ^2_{\mathrm { V}_\varepsilon } + \frac{1}{2\nu } \Vert \widetilde{f}_\varepsilon \Vert ^2_{\mathrm { V}^\prime _\varepsilon }, \end{aligned}$$

which we use in (91), to obtain

92 $$\begin{aligned} \begin{aligned} \Vert \widetilde{u}_\varepsilon (t)\Vert ^2_{{\mathbb {L}^2(Q_\varepsilon )}}&+ \nu \int _0^t \Vert \mathrm{curl}\;\widetilde{u}_\varepsilon (s)\Vert ^2_{{\mathbb {L}^2(Q_\varepsilon )}}\,ds \le \Vert \widetilde{u}_0^\varepsilon \Vert ^2_{{\mathbb {L}^2(Q_\varepsilon )}} + \frac{1}{\nu }\int _0^t \Vert \widetilde{f}_\varepsilon (s)\Vert ^2_{\mathrm { V}^\prime _\varepsilon }\,ds \\&\, + 2 \int _0^t \left( \widetilde{G}_\varepsilon (s)\,d\widetilde{W}_\varepsilon , \widetilde{u}_\varepsilon (s)\right) _{{\mathbb {L}^2(Q_\varepsilon )}} + \int _0^t \Vert \widetilde{G}_\varepsilon (s)\Vert ^2_{\mathcal {T}_2(\mathbb {R}^N; \mathrm { H}_\varepsilon )}\,ds. \end{aligned} \end{aligned}$$

Using the Burkholder–Davis–Gundy inequality (see [32, Prop. 2.12]), we have

93 $$\begin{aligned} \mathbb {E}\sup _{0\le t \le T}&\left| \int _0^t (\widetilde{G}_\varepsilon (s)\,d\widetilde{W}_\varepsilon (s) , \widetilde{u}_\varepsilon (s) )_{{\mathbb {L}^2(Q_\varepsilon )}} \right| \nonumber \\&\le C \mathbb {E}\left( \int _0^T \Vert \widetilde{u}_\varepsilon (s)\Vert ^2_{{\mathbb {L}^2(Q_\varepsilon )}} \Vert \widetilde{G}_\varepsilon (s)\Vert _{\mathcal {T}_2(\mathbb {R}^N; \mathrm { H}_\varepsilon )}^2\,ds\right) ^{1/2} \nonumber \\&\le C \mathbb {E}\left[ \left( \sup _{0\le t \le T} \Vert \widetilde{u}_\varepsilon (t)\Vert ^2_{{\mathbb {L}^2(Q_\varepsilon )}} \right) ^{1/2} \left( \int _0^T \Vert \widetilde{G}_\varepsilon (s)\Vert ^2_{\mathcal {T}_2(\mathbb {R}^N; \mathrm { H}_\varepsilon )}\,ds\right) ^{1/2} \right] \nonumber \\&\le \frac{1}{4} \mathbb {E}\left( \sup _{0 \le t \le T} \Vert \widetilde{u}_\varepsilon (t)\Vert ^2_{{\mathbb {L}^2(Q_\varepsilon )}} \right) + C\int _0^T \Vert \widetilde{G}_\varepsilon (s)\Vert ^2_{\mathcal {T}_2(\mathbb {R}^N; \mathrm { H}_\varepsilon )}\,ds. \end{aligned}$$

Taking the supremum of (92) over the interval [0, *T*], then taking expectation and using inequality (93) we infer the energy inequality (90). $\square$

Let us recall the following notations, which we introduced earlier, for $t \in [0,T]$94

#### Lemma 20

Let $\widetilde{u}_\varepsilon$ be a martingale solution of (82) and Assumption 1 hold, in particular, for $p =2$. Then

95 $$\begin{aligned} \begin{aligned}&\mathbb {E}\left[ \frac{1}{2} \sup _{t \in [0,T]} \Vert \alpha _\varepsilon (t)\Vert ^2_{{\mathbb {L}^2({\mathbb {S}^2})}} + \nu \int _0^T \Vert \mathrm{{curl}^\prime }\alpha _\varepsilon (s)\Vert ^2_{{\mathbb {L}^2({\mathbb {S}^2})}}\,ds \right] \le C_1^2 + \frac{C_2}{\nu } + C_3, \end{aligned} \end{aligned}$$

where $C_1,C_2$ are positive constants from (79) and (80) and $C_3 > 0$ (determined within the proof) is another constant independent of $\varepsilon$.

#### Proof

Let $\widetilde{u}_\varepsilon$ be a martingale solution of (82), then it satisfies the energy inequality (90). From Eq. (47), we have

96 $$\begin{aligned} \Vert \widetilde{\alpha }_\varepsilon (t)\Vert ^2_{{\mathbb {L}^2(Q_\varepsilon )}} \le \Vert \widetilde{u}_\varepsilon (t)\Vert ^2_{{\mathbb {L}^2(Q_\varepsilon )}}, \qquad t \in [0,T]. \end{aligned}$$

Moreover, by Corollary 3

97 $$\begin{aligned} \frac{4}{9} \Vert \mathrm{curl}\;\widetilde{\alpha }_\varepsilon (t)\Vert ^2_{\mathbb {L}^2(Q_\varepsilon )}\le \Vert \mathrm{curl}\;\widetilde{u}_\varepsilon (t)\Vert ^2_{\mathbb {L}^2(Q_\varepsilon )}, \qquad t \in [0,T]. \end{aligned}$$

Therefore, using (96) and (97) in the energy inequality (90), we get

$$\begin{aligned} \mathbb {E}&\left[ \frac{1}{2} \sup _{t \in [0,T]} \Vert \widetilde{\alpha }_\varepsilon (t)\Vert ^2_{{\mathbb {L}^2(Q_\varepsilon )}} + \frac{4 \nu }{9} \int _0^T \Vert \mathrm{curl}\;\widetilde{\alpha }_\varepsilon (s)\Vert ^2_{\mathbb {L}^2(Q_\varepsilon )}\,ds\right] \\&\le \Vert \widetilde{u}_0^\varepsilon \Vert ^2_{{\mathbb {L}^2(Q_\varepsilon )}} + \frac{1}{\nu } \int _0^T \Vert f_\varepsilon (s)\Vert ^2_{\mathrm { V}_\varepsilon ^\prime }\,ds + K \int _0^T\Vert \widetilde{G}_\varepsilon (s)\Vert ^2_{\mathcal {T}_2}\,ds, \end{aligned}$$

and hence from the scaling property, Lemma 11, we have

98 $$\begin{aligned} \mathbb {E}&\left[ \frac{1}{2} \varepsilon \sup _{t \in [0,T]} \Vert {\alpha }_\varepsilon (t)\Vert ^2_{{\mathbb {L}^2({\mathbb {S}^2})}} + \frac{4 \nu }{9} \varepsilon \int _0^T \Vert \mathrm{{curl}^\prime }{\alpha }_\varepsilon (s)\Vert ^2_{{\mathbb {L}^2({\mathbb {S}^2})}}\,ds\right] \nonumber \\&\le \Vert \widetilde{u}_0^\varepsilon \Vert ^2_{{\mathbb {L}^2(Q_\varepsilon )}} + \frac{1}{\nu } \int _0^T \Vert f_\varepsilon (s)\Vert ^2_{\mathrm { V}_\varepsilon ^\prime }\,ds + K \int _0^T\Vert \widetilde{G}_\varepsilon (s)\Vert ^2_{\mathcal {T}_2}\,ds. \end{aligned}$$

By the assumptions on $\widetilde{g}_\varepsilon ^j$ (81), there exists a positive constant *c* such that for every $j \in \{1, \ldots , N\}$

99 $$\begin{aligned} \int _0^T\Vert \widetilde{g}_\varepsilon ^j(t)\Vert _{\mathbb {L}^2(Q_\varepsilon )}^2\,dt \le c \varepsilon . \end{aligned}$$

Therefore, using Assumption 1 and (99) in (98), cancelling $\varepsilon$ on both sides and defining $C_3 = NKc$, we infer inequality (95). $\square$

From the results of Lemma 20, we deduce that

100 $$\begin{aligned} \left\{ {\alpha }_\varepsilon \right\} _{\varepsilon > 0}\, \text{ is } \text{ bounded } \text{ in } \, L^2(\varOmega ;L^\infty (0,T; \mathrm { H}) \cap L^2(0,T; \mathrm { V})). \end{aligned}$$

Since $\mathrm { V}$ can be embedded into $\mathbb {L}^6({\mathbb {S}^2})$, by using interpolation between $L^\infty (0,T; \mathrm { H})$ and $L^2(0,T; \mathbb {L}^6({\mathbb {S}^2}))$ we obtain

101 $$\begin{aligned} \mathbb {E}\int _0^T \Vert {\alpha }_\varepsilon (s)\Vert ^2_{\mathbb {L}^3({\mathbb {S}^2})}\,ds \le C. \end{aligned}$$

#### Lemma 21

Let $\widetilde{u}_\varepsilon$ be a martingale solution of (82) and Assumption 1 hold, in particular, for $p =2$. Then

102 $$\begin{aligned} \mathbb {E}\left[ \frac{1}{2}\sup _{t \in [0,T]}\Vert \widetilde{\beta }_\varepsilon (t)\Vert ^2_{{\mathbb {L}^2(Q_\varepsilon )}} + \nu \int _0^T \Vert \mathrm{curl}\;\widetilde{\beta }_\varepsilon (s)\Vert ^2_{{\mathbb {L}^2(Q_\varepsilon )}}\,ds\right] \le C_1^2 \varepsilon + \frac{C_2 \varepsilon }{\nu } + C_3\varepsilon . \end{aligned}$$

#### Proof

Let $\widetilde{u}_\varepsilon$ be a martingale solution of (82), then it satisfies the energy inequality (90). From (47), we have

103 $$\begin{aligned} \Vert \widetilde{\beta }_\varepsilon (t)\Vert ^2_{{\mathbb {L}^2(Q_\varepsilon )}} \le \Vert \widetilde{u}_\varepsilon (t)\Vert ^2_{{\mathbb {L}^2(Q_\varepsilon )}}, \qquad t \in [0,T]. \end{aligned}$$

Thus, by Corollary 3

104 $$\begin{aligned} \frac{4}{9} \Vert \mathrm{curl}\;\widetilde{\beta }_\varepsilon (t)\Vert ^2_{\mathbb {L}^2(Q_\varepsilon )}\le \Vert \mathrm{curl}\;\widetilde{u}_\varepsilon (t)\Vert ^2_{\mathbb {L}^2(Q_\varepsilon )}, \quad t \in [0,T]. \end{aligned}$$

Therefore, using Assumption 1, (99), inequalities (103)–(104), in the energy inequality (90), we infer (102). $\square$

In the following lemma we obtain some higher order estimates (on a formal level) for the martingale solution $\widetilde{u}_\varepsilon$, which will be used to obtain the higher order estimates for the processes ${\alpha }_\varepsilon$ and $\widetilde{\beta }_\varepsilon$.

#### Lemma 22

Let Assumption 1 hold true and $\widetilde{u}_\varepsilon$ be a martingale solution of (82). Then, for $p > 2$ we have following estimates

105 $$\begin{aligned} \mathbb {E}\sup _{0\le s\le T} \Vert \widetilde{u}_\varepsilon (s)\Vert ^p_{{\mathbb {L}^2(Q_\varepsilon )}} \le C_2\left( p, \widetilde{u}_0^\varepsilon , \widetilde{f}_\varepsilon ,\widetilde{G}_\varepsilon \right) \exp \left( K_p\, T\right) \end{aligned}$$

and

106 $$\begin{aligned} \mathbb {E}\int _0^T \Vert \widetilde{u}_\varepsilon (t)\Vert _{{\mathbb {L}^2(Q_\varepsilon )}}^{p-2}\Vert \widetilde{u}_\varepsilon (t)\Vert _{\mathrm { V}_\varepsilon }^2dt \le C_2\left( p, \widetilde{u}_0^\varepsilon , \widetilde{f}_\varepsilon ,\widetilde{G}_\varepsilon \right) \left[ 1 + K_{p}\,T\exp \left( K_{p}\,T\right) \right] , \end{aligned}$$

where

$$\begin{aligned}&C_2\left( p, \widetilde{u}_0^\varepsilon , \widetilde{f}_\varepsilon , \widetilde{G}_\varepsilon \right) := \Vert \widetilde{u}_0^\varepsilon \Vert ^p_{{\mathbb {L}^2(Q_\varepsilon )}} + \nu ^{-p/2}\Vert \widetilde{f}_\varepsilon \Vert ^p_{L^p(0,T; \mathrm { V}'_\varepsilon )}\\&\quad + \left( \frac{1}{4}p^2 (p-1) + \frac{K_1^2}{p}\right) \Vert \widetilde{G}_\varepsilon \Vert ^p_{L^p(0,T;\mathcal {T}_2)},\\&K_{p} := \left( \frac{K_1^2}{p}+p\right) \frac{(p-2)}{2}, \end{aligned}$$

and $K_1$ is a constant from the Burkholder–Davis–Gundy inequality.

#### Proof

Let $$F(x) = \Vert x\Vert ^p_{{\mathbb {L}^2(Q_\varepsilon )}}$$ then

$$\begin{aligned} \frac{\partial F}{\partial x} = \nabla F = p \Vert x\Vert ^{p-2}_{{\mathbb {L}^2(Q_\varepsilon )}} x, \end{aligned}$$

and

107 $$\begin{aligned} \left| \frac{\partial ^2 F}{\partial x^2} \right| \le p(p-1) \Vert x\Vert ^{p-2}_{{\mathbb {L}^2(Q_\varepsilon )}}. \end{aligned}$$

Applying the Itô lemma with *F*(*x*) and process $\widetilde{u}_\varepsilon$ for $t \in [0,T]$, we have

$$\begin{aligned}&\Vert \widetilde{u}_\varepsilon (t)\Vert ^p_{{\mathbb {L}^2(Q_\varepsilon )}} = \Vert \widetilde{u}_\varepsilon (0)\Vert ^p_{{\mathbb {L}^2(Q_\varepsilon )}} + p \int _0^t \Vert \widetilde{u}_\varepsilon (s)\Vert _{{\mathbb {L}^2(Q_\varepsilon )}}^{p-2} \\&\quad \langle -\nu \mathrm { A}_\varepsilon \widetilde{u}_\varepsilon (s) - B_\varepsilon (\widetilde{u}_\varepsilon (s),\widetilde{u}_\varepsilon (s)) + \widetilde{f}_\varepsilon (s), \widetilde{u}_\varepsilon (s) \rangle _\varepsilon \, ds \\&\quad + p \int _0^t \Vert \widetilde{u}_\varepsilon (s)\Vert ^{p-2}_{{\mathbb {L}^2(Q_\varepsilon )}} \left( \widetilde{u}_\varepsilon (s), \widetilde{G}_\varepsilon (s) d\widetilde{W}_\varepsilon (s) \right) _{{\mathbb {L}^2(Q_\varepsilon )}}\\&\quad + \frac{1}{2}\int _0^t \mathrm {Tr} \left( \frac{\partial ^2 F}{\partial x^2} (\widetilde{G}_\varepsilon (s),\widetilde{G}_\varepsilon (s))\right) \,ds . \end{aligned}$$

Using the fact that $\langle B_\varepsilon (\widetilde{u}_\varepsilon ,\widetilde{u}_\varepsilon ), \widetilde{u}_\varepsilon \rangle _\varepsilon = 0$ and $\langle A_\varepsilon \widetilde{u}_\varepsilon ,\widetilde{u}_\varepsilon \rangle _\varepsilon = \Vert \widetilde{u}_\varepsilon \Vert _{\mathrm { V}_\varepsilon }^2$ we arrive at

$$\begin{aligned}&\Vert \widetilde{u}_\varepsilon (t)\Vert ^p_{{\mathbb {L}^2(Q_\varepsilon )}} = \Vert \widetilde{u}_\varepsilon (0)\Vert ^p_{{\mathbb {L}^2(Q_\varepsilon )}} - p\nu \int _0^t \Vert \widetilde{u}_\varepsilon (s)\Vert _{{\mathbb {L}^2(Q_\varepsilon )}}^{p-2} \Vert \widetilde{u}_\varepsilon (s) \Vert _{\mathrm { V}_\varepsilon }^2\\&\quad + p \int _0^t \Vert \widetilde{u}_\varepsilon (s)\Vert _{{\mathbb {L}^2(Q_\varepsilon )}}^{p-2} \langle \widetilde{f}_\varepsilon (s), \widetilde{u}_\varepsilon (s) \rangle _\varepsilon \, ds \\&\quad + p \int _0^t \Vert \widetilde{u}_\varepsilon (s)\Vert ^{p-2}_{H_\varepsilon } \left( \widetilde{u}_\varepsilon (s), \widetilde{G}_\varepsilon (s) d\widetilde{W}_\varepsilon (s) \right) _{{\mathbb {L}^2(Q_\varepsilon )}}\\&\quad + \frac{1}{2} \int _0^t \mathrm {Tr} \left( \frac{\partial ^2 F}{\partial x^2} (\widetilde{G}_\varepsilon (s),\widetilde{G}_\varepsilon (s))\right) ds. \end{aligned}$$

Using (107) and the Cauchy–Schwarz inequality, we get

$$\begin{aligned}&\Vert \widetilde{u}_\varepsilon (t)\Vert ^p_{{\mathbb {L}^2(Q_\varepsilon )}} + p \nu \int _0^t \Vert \widetilde{u}_\varepsilon (s)\Vert _{{\mathbb {L}^2(Q_\varepsilon )}}^{p-2} \Vert \widetilde{u}_\varepsilon (s)\Vert _{\mathrm { V}_\varepsilon }^2 ds\\&\quad \le \Vert \widetilde{u}_\varepsilon (0)\Vert ^p_{{\mathbb {L}^2(Q_\varepsilon )}} + p \int _0^t \Vert \widetilde{u}_\varepsilon (s)\Vert _{{\mathbb {L}^2(Q_\varepsilon )}}^{p-2} \Vert \widetilde{f}_\varepsilon (s)\Vert _{\mathrm { V}'_\varepsilon } \Vert \widetilde{u}_\varepsilon (s)\Vert _{\mathrm { V}_\varepsilon }\,ds \\&\qquad + p \int _0^t \Vert \widetilde{u}_\varepsilon (s)\Vert _{{\mathbb {L}^2(Q_\varepsilon )}}^{p-2} \left( \widetilde{u}_\varepsilon (s), \widetilde{G}_\varepsilon (s) d\widetilde{W}_\varepsilon (s) \right) _{\mathbb {L}^2(Q_\varepsilon )}\\&\qquad + \frac{p(p-1)}{2} \int _0^t \Vert \widetilde{u}_\varepsilon (s)\Vert _{{\mathbb {L}^2(Q_\varepsilon )}}^{p-2} \Vert \widetilde{G}_\varepsilon (s)\Vert ^2_{\mathcal {T}_2(\mathbb {R}^N; \mathrm { H}_\varepsilon )}\, ds, \end{aligned}$$

where we recall

$$\begin{aligned}\Vert \widetilde{G}_\varepsilon (s)\Vert ^2_{\mathcal {T}_2(\mathbb {R}^N; \mathrm { H}_\varepsilon )} = \sum _{j=1}^N \Vert \widetilde{g}_\varepsilon ^j(s)\Vert ^2_{{\mathbb {L}^2(Q_\varepsilon )}}. \end{aligned}$$

Using the generalised Young inequality $abc \le a^q/q + b^r/r + c^s/s$ (where $1/q+1/r+1/s=1$) with $a = \sqrt{\nu } \Vert \widetilde{u}_\varepsilon \Vert _{{\mathbb {L}^2(Q_\varepsilon )}}^{p/2-1} \Vert \widetilde{u}_\varepsilon \Vert _{\mathrm { V}_\varepsilon }$, $b=\Vert \widetilde{u}_\varepsilon \Vert _{{\mathbb {L}^2(Q_\varepsilon )}}^{p/2-1}$, $c = \frac{1}{\sqrt{\nu }}\Vert f_\varepsilon \Vert _{\mathrm { V}'_\varepsilon }$ and exponents $q=2, r=p, s=2p/(p-2)$ we get

108 $$\begin{aligned} \nu \Vert \widetilde{u}_\varepsilon \Vert _{{\mathbb {L}^2(Q_\varepsilon )}}^{p-2} \Vert f_\varepsilon \Vert _{\mathrm { V}'_\varepsilon } \Vert \widetilde{u}_\varepsilon \Vert _{\mathrm { V}_\varepsilon } \le \frac{\nu }{2} \Vert \widetilde{u}_\varepsilon \Vert _{{\mathbb {L}^2(Q_\varepsilon )}}^{p-2} \Vert \widetilde{u}_\varepsilon \Vert _{\mathrm { V}_\varepsilon }^2 + \frac{1}{p\nu ^{p/2}} \Vert \widetilde{f}_\varepsilon \Vert ^p_{\mathrm { V}'_\varepsilon } + \frac{p-2}{2p} \Vert \widetilde{u}_\varepsilon \Vert _{{\mathbb {L}^2(Q_\varepsilon )}}^p. \end{aligned}$$

Again using the Young inequality with exponents $p/(p-2)$, *p*/2 we get

109 $$\begin{aligned} \Vert \widetilde{u}_\varepsilon \Vert _{{\mathbb {L}^2(Q_\varepsilon )}}^{p-2}\Vert \widetilde{G} _\varepsilon \Vert ^2_{\mathcal {T}_2(\mathbb {R}^N; \mathrm { H}_\varepsilon )} \le \frac{p-2}{p} \Vert \widetilde{u}_\varepsilon \Vert _{{\mathbb {L}^2(Q_\varepsilon )}}^p + \frac{p}{2} \Vert \widetilde{G}_\varepsilon \Vert ^p_{\mathcal {T}_2(\mathbb {R}^N; \mathrm { H}_\varepsilon )}. \end{aligned}$$

Using (108) and (109) we obtain

110 $$\begin{aligned} \begin{aligned}&\Vert \widetilde{u}_\varepsilon (t)\Vert ^p_{{\mathbb {L}^2(Q_\varepsilon )}} + \frac{p \nu }{2} \int _0^t \Vert \widetilde{u}_\varepsilon (s)\Vert _{{\mathbb {L}^2(Q_\varepsilon )}}^{p-2} \Vert \widetilde{u}_\varepsilon (s)\Vert _{\mathrm { V}_\varepsilon }^2 ds \\&\quad \le \Vert \widetilde{u}_\varepsilon (0)\Vert ^p_{{\mathbb {L}^2(Q_\varepsilon )}} + \frac{p(p-2)}{2} \int _0^t \Vert \widetilde{u}_\varepsilon (s)\Vert _{{\mathbb {L}^2(Q_\varepsilon )}}^p\,ds + \nu ^{-p/2} \int _0^t \Vert \widetilde{f}_\varepsilon (s)\Vert ^p_{\mathrm { V}'_\varepsilon }\, ds \\&\qquad + \frac{1}{4}p^2(p-1) \int _0^t \Vert \widetilde{G}_\varepsilon (s)\Vert ^p_{\mathcal {T}_2(\mathbb {R}^N; \mathrm { H}_\varepsilon )}\, ds \\&\qquad + p \int _0^t \Vert \widetilde{u}_\varepsilon (s)\Vert _{{\mathbb {L}^2(Q_\varepsilon )}}^{p-2} \left( \widetilde{u}_\varepsilon (s),\widetilde{G}_\varepsilon (s) d\widetilde{W}_\varepsilon (s) \right) _{\mathbb {L}^2(Q_\varepsilon )}. \end{aligned} \end{aligned}$$

Since $\widetilde{u}_\varepsilon$ is a martingale solution of (82) it satisfies the energy inequality (90), hence the real-valued random variable

$$\begin{aligned} \mu _\varepsilon (t) = \int _0^t \Vert \widetilde{u}_\varepsilon (s)\Vert _{{\mathbb {L}^2(Q_\varepsilon )}}^{p-2} \left( \widetilde{u}_\varepsilon (s), \widetilde{G}_\varepsilon (s) d\widetilde{W}_\varepsilon (s) \right) _{\mathbb {L}^2(Q_\varepsilon )}\end{aligned}$$

is a $\mathcal {F}_t$-martingale. Taking expectation both sides of (110) we obtain

111 $$\begin{aligned} \begin{aligned}&\mathbb {E}\Vert \widetilde{u}_\varepsilon (t)\Vert ^p_{{\mathbb {L}^2(Q_\varepsilon )}} + \frac{p \nu }{2} \mathbb {E}\int _0^t \Vert \widetilde{u}_\varepsilon (s)\Vert _{{\mathbb {L}^2(Q_\varepsilon )}}^{p-2} \Vert \widetilde{u}_\varepsilon (s)\Vert _{\mathrm { V}_\varepsilon }^2 ds \\&\qquad \le \Vert \widetilde{u}_\varepsilon (0)\Vert ^p_{{\mathbb {L}^2(Q_\varepsilon )}} + \frac{p(p-2)}{2} \mathbb {E}\int _0^t \Vert \widetilde{u}_\varepsilon (s)\Vert _{{\mathbb {L}^2(Q_\varepsilon )}}^p ds + \nu ^{-p/2} \int _0^t \Vert \widetilde{f}_\varepsilon (s)\Vert ^p_{\mathrm { V}'_\varepsilon }\,ds \\&\qquad \quad + \frac{1}{4}p^2 (p-1) \int _0^t \Vert \widetilde{G}_\varepsilon (s)\Vert ^p_{\mathcal {T}_2(\mathbb {R}^N; \mathrm { H}_\varepsilon )}\,ds. \end{aligned} \end{aligned}$$

Therefore, by the Gronwall lemma we obtain

$$\begin{aligned} \mathbb {E}\Vert \widetilde{u}_\varepsilon (t)\Vert ^p_{{\mathbb {L}^2(Q_\varepsilon )}} \le C\left( \widetilde{u}_0^\varepsilon , \widetilde{f}_\varepsilon ,\widetilde{G}_\varepsilon \right) \exp \left( p\frac{(p-2)t}{2}\right) , \end{aligned}$$

where

$$\begin{aligned} C\left( \widetilde{u}_0^\varepsilon , \widetilde{f}_\varepsilon ,\widetilde{G}_\varepsilon \right) := \Vert \widetilde{u}_0^\varepsilon \Vert _{{\mathbb {L}^2(Q_\varepsilon )}}^p + \nu ^{-p/2} \Vert f\Vert ^p_{L^p(0,T; \mathrm { V}'_\varepsilon )} + \frac{1}{4} p^2(p-1) \Vert \widetilde{G}_\varepsilon \Vert ^p_{L^p(0,T;\mathcal {T}_2(\mathbb {R}^N; \mathrm { H}_\varepsilon ))} . \end{aligned}$$

By Burkholder–Davis–Gundy inequality, we have

112 $$\begin{aligned} \mathbb {E}&\left( \sup _{0\le s \le t}\left| \int _0^s \Vert \widetilde{u}_\varepsilon (\sigma )\Vert ^{p-2}_{{\mathbb {L}^2(Q_\varepsilon )}} \left( \widetilde{u}_\varepsilon (\sigma ), \widetilde{G}_\varepsilon (\sigma ) d\widetilde{W}_\varepsilon (\sigma ) \right) _{\mathbb {L}^2(Q_\varepsilon )}\right| \right) \nonumber \\&\le K_1 \mathbb {E}\left( \int _0^t \Vert \widetilde{u}_\varepsilon (s)\Vert _{{\mathbb {L}^2(Q_\varepsilon )}}^{2p-4} |\widetilde{u}_\varepsilon (s)|^2_{{\mathbb {L}^2(Q_\varepsilon )}} \Vert \widetilde{G}_\varepsilon (s)\Vert ^2_{\mathcal {T}_2(\mathbb {R}^N; \mathrm { H}_\varepsilon )}\,ds \right) ^{1/2} \nonumber \\&\le K_1 \mathbb {E}\left[ \sup _{0\le s\le t} \Vert \widetilde{u}_\varepsilon (s)\Vert _{{\mathbb {L}^2(Q_\varepsilon )}}^{p/2} \left( \int _0^t \Vert \widetilde{u}_\varepsilon (s)\Vert _{{\mathbb {L}^2(Q_\varepsilon )}}^{p-2} \Vert \widetilde{G}_\varepsilon (s)\Vert ^2_{\mathcal {T}_2(\mathbb {R}^N; \mathrm { H}_\varepsilon )} ds \right) ^{1/2} \right] \nonumber \\&\le \frac{1}{2} \mathbb {E}\sup _{0\le s \le t} \Vert \widetilde{u}_\varepsilon (s)\Vert _{{\mathbb {L}^2(Q_\varepsilon )}}^p + \frac{K^2_1}{2} \mathbb {E}\int _0^t \Vert \widetilde{u}_\varepsilon (s)\Vert _{{\mathbb {L}^2(Q_\varepsilon )}}^{p-2} \Vert \widetilde{G}_\varepsilon (s)\Vert ^2_{\mathcal {T}_2(\mathbb {R}^N; \mathrm { H}_\varepsilon )}\, ds \nonumber \\&\le \frac{1}{2} \mathbb {E}\sup _{0\le s \le t} \Vert \widetilde{u}_\varepsilon (s)\Vert ^p_{{\mathbb {L}^2(Q_\varepsilon )}} \nonumber \\&\quad + \frac{K_1^2}{2} \frac{(p-2)}{p} \mathbb {E}\int _0^t \Vert \widetilde{u}_\varepsilon (s)\Vert _{{\mathbb {L}^2(Q_\varepsilon )}}^p ds + \frac{K_1^2}{p} \int _0^t \Vert \widetilde{G}_\varepsilon (s)\Vert ^p_{\mathcal {T}_2(\mathbb {R}^N; \mathrm { H}_\varepsilon )}\,ds \end{aligned}$$

where in the last step we have used the Young inequality with exponents $p/(p-2)$ and *p*/2.

Taking supremum over $0\le s\le t$ in (110) and using (112) we get

113 $$\begin{aligned} \frac{1}{2} \mathbb {E}&\sup _{0 \le s\le t} \Vert \widetilde{u}_\varepsilon (s)\Vert ^p_{{\mathbb {L}^2(Q_\varepsilon )}} + \frac{\nu }{p} \mathbb {E}\int _0^t \Vert \widetilde{u}_\varepsilon (s)\Vert _{{\mathbb {L}^2(Q_\varepsilon )}}^{p-2} \Vert \widetilde{u}_\varepsilon (s)\Vert ^2_{\mathrm { V}_\varepsilon } ds \nonumber \\&\le \Vert \widetilde{u}_\varepsilon (0)\Vert ^p_{{\mathbb {L}^2(Q_\varepsilon )}} + \left( \frac{K_1^2}{p} + p \right) \frac{(p-2)}{2} \int _0^t \mathbb {E}\sup _{0 \le s \le \sigma } \Vert \widetilde{u}_\varepsilon (s)\Vert _{{\mathbb {L}^2(Q_\varepsilon )}}^p d\sigma \nonumber \\&\qquad + \nu ^{-p/2} \int _0^t \Vert \widetilde{f}_\varepsilon (s)\Vert ^p_{\mathrm { V}'_\varepsilon } ds + \left( \frac{1}{4}p^2(p-1) + \frac{K_1^2}{p}\right) \int _0^t \Vert \widetilde{G}_\varepsilon (s)\Vert ^p_{\mathcal {T}_2(\mathbb {R}^N; \mathrm { H}_\varepsilon )}\,ds. \end{aligned}$$

Thus using the Gronwall lemma, we obtain

$$\begin{aligned} \mathbb {E}\sup _{0\le s\le t} \Vert \widetilde{u}_\varepsilon (s)\Vert ^p_{{\mathbb {L}^2(Q_\varepsilon )}} \le C_2\left( p, \widetilde{u}_0^\varepsilon , \widetilde{f}_\varepsilon ,\widetilde{G}_\varepsilon \right) \exp \left( K_p\,t\right) , \end{aligned}$$

where $C_2\left( p, \widetilde{u}_0^\varepsilon , \widetilde{f}_\varepsilon ,\widetilde{G}_\varepsilon \right)$ and $K_p$ are the constants as defined in the statement of lemma. We deduce (106) from (113) and (105). $\square$

In the following lemma we will use the estimates from previous lemma to obtain higher order estimates for $\alpha _\varepsilon$ and $\widetilde{\beta }_\varepsilon$.

#### Lemma 23

Let $p > 2$. Let $\widetilde{u}_\varepsilon$ be a martingale solution of (82) and Assumption 1 hold with the chosen *p*. Then, the processes ${\alpha }_\varepsilon$ and $\widetilde{\beta }_\varepsilon$ (as defined in (94)) satisfy the following estimates

114 $$\begin{aligned} \mathbb {E}\sup _{t\in [0,T]}\Vert {\alpha }_\varepsilon (t)\Vert ^p_{{\mathbb {L}^2({\mathbb {S}^2})}} \le K(\nu , p)\exp \left( K_p\,T\right) , \end{aligned}$$

and

115 $$\begin{aligned} \mathbb {E}\sup _{t\in [0,T]}\Vert \widetilde{\beta }_\varepsilon (t)\Vert ^p_{{\mathbb {L}^2(Q_\varepsilon )}} \le \varepsilon ^{p/2}K(\nu , p)\exp \left( K_p\,T\right) , \end{aligned}$$

where $K(\nu ,p)$ is a positive constant independent of $\varepsilon$ and $K_p$ is defined in Lemma 22.

#### Proof

The lemma can be proved following the steps of Lemmas 20 and 21 with the use of Proposition 1, scaling property from Lemma 11, the Cauchy–Schwarz inequality, Assumptions 1, 2 and the estimates obtained in Lemma 22. $\square$

### Tightness

In this subsection we will prove that the family of laws induced by the processes $\alpha _\varepsilon$ is tight on an appropriately chosen topological space $\mathcal {Z}_T$. In order to do so we will consider the following functional spaces for fixed $T > 0$:

$\mathcal {C}([0,T]; {\mathrm {D}(\mathrm { A}^{-1})}) :=$ the space of continuous functions $u: [0,T] \rightarrow {\mathrm {D}(\mathrm { A}^{-1})}$ with the topology $\mathbf {T}_1$ induced by the norm $\Vert u\Vert _{\mathcal {C}([0,T]; {\mathrm {D}(\mathrm { A}^{-1})})} := \sup _{t \in [0,T]}\Vert u(t)\Vert _{{\mathrm {D}(\mathrm { A}^{-1})}}$,

$L^2_{\mathrm {w}}(0,T; \mathrm { V}) :=$ the space $L^2(0,T; \mathrm { V})$ with the weak topology $\mathbf {T}_2$,

$L^2(0,T; \mathrm { H}) :=$ the space of measurable functions $u : [0,T] \rightarrow \mathrm {H}$ such that

$$\begin{aligned} \Vert u\Vert _{L^2(0,T; \mathrm {H})} = \left( \int _0^T \Vert u(t)\Vert _{{\mathbb {L}^2({\mathbb {S}^2})}}^2\,dt \right) ^{\frac{1}{2}} < \infty , \end{aligned}$$

with the topology $\mathbf {T}_3$ induced by the norm $\Vert u\Vert _{L^2(0,T; \mathrm {H})}$.

Let $\mathrm { H}_\mathrm {w}$ denote the Hilbert space $\mathrm { H}$ endowed with the weak topology.

$\mathcal {C}([0,T]; \mathrm {H_w}) :=$ the space of weakly continuous functions $u: [0,T] \rightarrow \mathrm {H}$ endowed with the weakest topology $\mathbf {T}_4$ such that for all $h \in \mathrm {H}$ the mappings

$$\begin{aligned} \mathcal {C}([0,T]; \mathrm {H_w}) \ni u \rightarrow \left( u(\cdot ), h \right) _{{\mathbb {L}^2({\mathbb {S}^2})}} \in \mathcal {C}([0,T]; \mathbb {R}) \end{aligned}$$

are continuous. In particular $u_n \rightarrow u$ in $\mathcal {C}([0,T]; \mathrm { H}_\mathrm {w})$ iff for all $h \in \mathrm { H}:$

$$\begin{aligned} \lim _{n \rightarrow \infty } \sup _{t \in [0,T]} \left| \left( u_n(t) - u(t), h \right) _{{\mathbb {L}^2({\mathbb {S}^2})}} \right| = 0. \end{aligned}$$

Let

116 $$\begin{aligned} \mathcal {Z}_T = \mathcal {C}([0,T]; \mathrm {D}(\mathrm { A}^{-1})) \cap L^2_{\mathrm {w}}(0,T; \mathrm { V}) \cap L^2(0,T; \mathrm { H}) \cap \mathcal {C}([0,T]; \mathrm {H_w}), \end{aligned}$$

and let $\mathcal {T}$ be the supremum^3^ of the corresponding topologies.

#### Lemma 24

The set of measures $\left\{ \mathcal {L}(\alpha _\varepsilon ),\, \varepsilon \in (0,1]\right\}$ is tight on $\left( \mathcal {Z}_T, \mathcal {T}\right)$.

#### Proof

Let $\widetilde{u}_\varepsilon$, for some fixed $\varepsilon >0$, be a martingale solution of problem (82). Let us choose and fix $\phi \in \mathrm {D}(\mathrm { A})$. Then, recalling the definition (40) of the operator $\widetilde{N}_\varepsilon$, by Lemma 9, we infer that for $t\in [0,T]$ we have117Similarly we have, also for $t\in [0,T]$,118Thus, by Proposition 1, identity (83), equalities (117), (118), and the notations from (94), we infer that martingale solution $\widetilde{u}_\varepsilon$ satisfies the following equality, for $t \in [0,T]$, $\mathbb {P}$-a.s.119The proof of lemma turns out to be a direct application of Corollary 6. Indeed, by Lemma 20, assumptions (*a*) and (*b*) of Corollary 6 are satisfied and therefore, it is sufficient to show that the sequence $\left( \alpha _\varepsilon \right) _{\varepsilon > 0}$ satisfies the Aldous condition $[\mathbf{A} ]$, see Definition 6, in space $\mathrm {D}(\mathrm { A}^{-1})$.

Let $\theta \in (0,T)$ and $\left( \tau _\varepsilon \right) _{\varepsilon > 0}$ be a sequence of stopping times such that $0 \le \tau _\varepsilon \le \tau _\varepsilon +\theta \le T$. We start by estimating each term in the R.H.S. of (119). We will use the Hölder inequality, the scaling property from Lemma 11, the Poincaré type inequality (54), the Ladyzhenskaya inequality (55), inequality (56), the a priori estimates from Lemmas 20, 21, results from Lemmas 17 and 18.

In what follows, we will prove that each of the eight process from equality (119) satisfies the Aldous condition $[\mathbf{A} ]$. In order to help the reader, we will divide the following part of the proof into eight parts.

- For the first term, we obtain 120
- Similarly for the second term we have 121
- Now we consider the first non-linear term. 122
- Similarly for the second non-linear term, we have 123
- Now as in the previous case, for the next mixed non-linear term, we obtain 124
- Finally, for the last non-linear term, we get 125
- Now for the term corresponding to the external forcing $\widetilde{f}_\varepsilon$, we have using the radial invariance of $M_\varepsilon \widetilde{f}_\varepsilon$ and assumption (80) 126
- At the very end we are left to deal with the last term corresponding to the stochastic forcing. Using the radial invariance of $M_\varepsilon \widetilde{g}_\varepsilon ^j$, Itô isometry, scaling (see Lemma 11) and assumption (81), we get 127
  128 $$\begin{aligned}&\le \mathbb {E}\left( \int _{\tau _\varepsilon }^{{\tau _\varepsilon }+\theta } \sum _{j=1}^N \Vert \widetilde{g}_\varepsilon ^j(s)\Vert ^2_{\mathbb {L}^2(Q_\varepsilon )}\,ds\right) \Vert \phi \Vert _{\mathbb {L}^2({\mathbb {S}^2})}^2 \nonumber \\&\le \varepsilon Nc \Vert \phi \Vert _{\mathrm {D}(\mathrm { A})}^2 \theta := c_8 \varepsilon \cdot \theta \Vert \phi \Vert _{\mathrm {D}(\mathrm { A})}^2 . \end{aligned}$$

After having proved what we had promised, we are ready to conclude the proof of Lemma 24. Since for every $t > 0$one has for $\phi \in \mathrm {D}(\mathrm { A})$,129Let us fix $\kappa > 0$ and $\gamma > 0$. By equality (119), the sigma additivity property of probability measure and (129), we have

$$\begin{aligned}&\mathbb {P} \left( \left\{ \Vert {\alpha }_\varepsilon (\tau _\varepsilon + \theta ) - {\alpha }_\varepsilon (\tau _\varepsilon )\Vert _{{\mathrm {D}(\mathrm { A}^{-1})}} \ge \kappa \right\} \right) \\&\quad \le \frac{1}{\varepsilon } \sum _{i=1}^8 \mathbb {P} \left( \left\{ \sup _{\Vert \phi \Vert _{\mathrm {D}(\mathrm { A})} = 1} \left| J_i^\varepsilon (\tau _\varepsilon + \theta ) - J_i^\varepsilon (\tau _\varepsilon )\right| \ge \kappa \right\} \right) . \end{aligned}$$

Using the Chebyshev’s inequality, we get

130 $$\begin{aligned} \mathbb {P} \left( \left\{ \Vert {\alpha }_\varepsilon (\tau _\varepsilon + \theta ) - {\alpha }_\varepsilon (\tau _\varepsilon )\Vert _{{\mathrm {D}(\mathrm { A}^{-1})}} \ge \kappa \right\} \right)&\le \frac{1}{\kappa \varepsilon } \sum _{i=1}^7 \mathbb {E}\left( \sup _{\Vert \phi \Vert _{\mathrm {D}(\mathrm { A})} = 1} \left| J_i^\varepsilon (\tau _\varepsilon + \theta ) - J_i^\varepsilon (\tau _\varepsilon )\right| \right) \nonumber \\&\quad + \frac{1}{\kappa ^2 \varepsilon } \mathbb {E}\left( \sup _{\Vert \phi \Vert _{\mathrm {D}(\mathrm { A})} = 1} \left| J_8^\varepsilon (\tau _\varepsilon + \theta ) - J_8^\varepsilon (\tau _\varepsilon )\right| ^2\right) . \end{aligned}$$

Thus, using estimates (120)–(128) in (130), we get

131 $$\begin{aligned}&\mathbb {P} \left( \left\{ \Vert {\alpha }_\varepsilon (\tau _\varepsilon + \theta ) - {\alpha }_\varepsilon (\tau _\varepsilon )\Vert _{{\mathrm {D}(\mathrm { A}^{-1})}} \ge \kappa \right\} \right) \nonumber \\&\quad \le \frac{1}{\kappa \varepsilon } \varepsilon \theta ^{1/2} \left[ c_1 \theta ^{1/2} + c_2 \varepsilon + c_3 \theta ^{1/4} + c_4 \varepsilon ^{2/3} + c_5 \varepsilon ^{2/3} + c_6 \varepsilon ^{2/3} + c_7 \right] + \frac{1}{\kappa ^2 \varepsilon } c_8 \varepsilon \theta . \end{aligned}$$

Let $\delta _i = \left( \dfrac{\kappa }{8 c_i} \gamma \right) ^2$, for $i = 1, \ldots , 7$ and $\delta _8 = \dfrac{\kappa ^2}{8 c_8} \gamma$. Choose $\delta = \max _{i \in \{1, \ldots , 8\}}\delta _i$. Hence,

$$\begin{aligned} \sup _{\varepsilon > 0} \sup _{0 \le \theta \le \delta } \mathbb {P} \left( \left\{ \Vert {\alpha }_\varepsilon (\tau _\varepsilon + \theta ) - {\alpha }_\varepsilon (\tau _\varepsilon )\Vert _{{\mathrm {D}(\mathrm { A}^{-1})}} \ge \kappa \right\} \right) \le \gamma . \end{aligned}$$

Since ${\alpha }_\varepsilon$ satisfies the Aldous condition $[\mathbf{A} ]$ in ${\mathrm {D}(\mathrm { A}^{-1})}$, we conclude the proof of Lemma 24 by invoking Corollary 6. $\square$

### Proof of Theorem 3

For every $\varepsilon > 0$, let us define the following intersection of spaces

132 $$\begin{aligned} \mathcal {Y}_T^\varepsilon = L^2_w(0,T; \mathrm { V}_\varepsilon ) \cap \mathcal {C}([0,T]; \mathrm { H}_\varepsilon ^w). \end{aligned}$$

Now, choose a countable subsequence $\left\{ \varepsilon _k\right\} _{k \in \mathbb {N}}$ converging to 0. For this subsequence define a product space $\mathcal {Y}_T$ given by

$$\begin{aligned} \mathcal {Y}_T = \varPi _{k \in \mathbb {N}} \mathcal {Y}_T^{\varepsilon _k}, \end{aligned}$$

and $$\eta :\varOmega \rightarrow \mathcal {Y}_T$$ by

$$\begin{aligned} \eta (\omega ) = \left( \widetilde{\beta }_{\varepsilon _1}(\omega ), \widetilde{\beta }_{\varepsilon _2}(\omega ), \ldots , \right) \in \mathcal {Y}_T. \end{aligned}$$

Now with this $\mathcal {Y}_T$-valued function we define a constant $\mathcal {Y}_T$-sequence

$$\begin{aligned} \eta _k \equiv \eta , \quad k \in \mathbb {N}. \end{aligned}$$

Then by Lemma 24 and the definition of sequence $\eta _k$, the set of measures $\left\{ \mathcal {L}\left( \alpha _{\varepsilon _k}, \eta _k\right) , k \in \mathbb {N}\right\}$ is tight on $\mathcal {Z}_T \times \mathcal {Y}_T$.

Thus, by the Jakubowski–Skorohod theorem^4^ there exists a subsequence $\left( k_n\right) _{n \in \mathbb {N}}$, a probability space $(\widehat{\varOmega }, \widehat{\mathcal {F}}, \widehat{\mathbb {P}})$ and, on this probability space, $\mathcal {Z}_T \times \mathcal {Y}_T \times \mathcal {C}([0,T]; \mathbb {R}^N)$-valued random variables $(\widehat{u}, \widehat{\eta }, \widehat{W})$, $\left( \widehat{\alpha }_{\varepsilon _{k_n}}, \widehat{\eta }_{k_n}, \widehat{W}_{\varepsilon _{k_n}}\right) , n \in \mathbb {N}$ such that

133 $$\begin{aligned} \begin{aligned}&\left( \widehat{\alpha }_{\varepsilon _{k_n}}, \widehat{\eta }_{k_n}, \widehat{W}_{\varepsilon _{k_n}}\right) \, \text{ has } \text{ the } \text{ same } \text{ law } \text{ as } \, \left( {\alpha }_{\varepsilon _{k_n}}, \eta _{k_n}, \widetilde{W}\right) \\&\text{ on } \mathcal {B}\left( \mathcal {Z}_T \times \mathcal {Y}_T \times \mathcal {C}([0,T]; \mathbb {R}^N)\right) \end{aligned} \end{aligned}$$

and

134 $$\begin{aligned} \left( \widehat{\alpha }_{\varepsilon _{k_n}}, \widehat{\eta }_{k_n}, \widehat{W}_{\varepsilon _{k_n}}\right) \rightarrow \left( \widehat{u}, \widehat{\eta }, \widehat{W} \right) \, \text{ in } \,\mathcal {Z}_T \times \mathcal {Y}_T \times \mathcal {C}([0,T]; \mathbb {R}^N),\quad \widehat{\mathbb {P}}\text{-a.s. } \end{aligned}$$

In particular, using marginal laws, and definition of the process $\eta _k$, we have

135 $$\begin{aligned} \mathcal {L}\left( \widehat{\alpha }_{\varepsilon _{k_n}}, \widehat{\beta }_{\varepsilon _{k_n}}\right) = \mathcal {L}\left( \alpha _{\varepsilon _{k_n}}, \widetilde{\beta }_{\varepsilon _{k_n}}\right) \; \text{ on } \mathcal {B}\left( \mathcal {Z}_T \times \mathcal {Y}_T^{{\varepsilon _{k_n}}}\right) \end{aligned}$$

where $\widehat{\beta }_{\varepsilon _{k_n}}$ is the $k_n$th component of $\mathcal {Y}_T$-valued random variable $\widehat{\eta }_{k_n}$. We are not interested in the limiting process $\widehat{\eta }$ and hence will not discuss it further.

Using the equivalence of law of $\widehat{W}_{\varepsilon _{k_n}}$ and $\widetilde{W}$ on $\mathcal {C}([0,T];\mathbb {R}^N)$ for $n \in \mathbb {N}$ one can show that $\widehat{W}$ and $\widehat{W}_{\varepsilon _{k_n}}$ are $\mathbb {R}^N$-valued Wiener processes (see [8, Lemma 5.2 and Proof] for details).

$\widehat{\alpha }_{\varepsilon _{k_n}} \rightarrow \widehat{u}$ in $\mathcal {Z}_T$, $\widehat{\mathbb {P}}\text{-a.s. }$ precisely means that

$$\begin{aligned} \widehat{\alpha }_{\varepsilon _{k_n}} \rightarrow \widehat{u}\; \text{ in } \,\mathcal {C}([0,T]; \mathrm {D}(\mathrm { A}^{-1})), \qquad \qquad&\widehat{\alpha }_{\varepsilon _{k_n}} \rightharpoonup \widehat{u}\; \text{ in } \,L^2(0,T; \mathrm { V}),\\ \widehat{\alpha }_{\varepsilon _{k_n}} \rightarrow \widehat{u}\; \text{ in } \,L^2(0,T; \mathrm { H}), \qquad \qquad&\widehat{\alpha }_{\varepsilon _{k_n}} \rightarrow \widehat{u}\; \text{ in } \,\mathcal {C}([0,T]; \mathrm { H}_w), \end{aligned}$$

and

$$\begin{aligned}\widehat{W}_{\varepsilon _{k_n}} \rightarrow \widehat{W}\, \text{ in } \,\mathcal {C}([0,T]; \mathbb {R}^N).\end{aligned}$$

Let us denote the subsequence $(\widehat{\alpha }_{\varepsilon _{k_n}}, \widehat{\beta }_{\varepsilon _{k_n}}, \widehat{W}_{\varepsilon _{k_n}})$ again by $(\widehat{\alpha }_\varepsilon , \widehat{\beta }_\varepsilon , \widehat{W}_\varepsilon )_{\varepsilon \in (0,1]}$.

Note that since $\mathcal {B}\left( \mathcal {Z}_T \times \mathcal {Y}_T \times \mathcal {C}([0,T]; \mathbb {R}^N) \right) \subset \mathcal {B}(\mathcal {Z}_T) \times \mathcal {B}(\mathcal {Y}_T)\times \mathcal {B}\left( \mathcal {C}([0,T]; \mathbb {R}^N)\right)$, the functions $\widehat{u}$, $\widehat{\eta }$ are $\mathcal {Z}_T$, $\mathcal {Y}_T$ Borel random variables respectively.

Using the retract operator  as defined in (63)–(65), we define new processes $\underline{\widehat{\alpha }_\varepsilon }$ corresponding to old processes $\widetilde{\alpha }_\varepsilon$ on the new probability space as follows136Moreover, by Lemma 16 we have the following scaling property for these new processes, i.e.

137 $$\begin{aligned} \Vert \underline{\widehat{\alpha }_\varepsilon }\Vert _{{\mathbb {L}^2(Q_\varepsilon )}} = \sqrt{\varepsilon }\Vert \widehat{\alpha }_\varepsilon \Vert _{{\mathbb {L}^2({\mathbb {S}^2})}}. \end{aligned}$$

The following auxiliary result which is needed in the proof of Theorem 3, cannot be deduced directly from the Kuratowski Theorem (see Theorem 7).

#### Lemma 25

Let $T > 0$ and $\mathcal {Z}_T$ be as defined in (116). Then the following sets $\mathcal {C}([0,T];\mathrm { H}) \cap \mathcal {Z}_T$, $L^2(0,T; \mathrm { V}) \cap \mathcal {Z}_T$ are Borel subsets of $\mathcal {Z}_T$.

#### Proof

See Appendix E.2. $$\square $$

By Lemma 25, $\mathcal {C}([0,T]; \mathrm { H})$ is a Borel subset of $\mathcal {Z}_T$. Since ${\alpha }_\varepsilon \in \mathcal {C}([0,T]; \mathrm { H})$, $\widehat{\mathbb {P}}$-a.s. and $\widehat{\alpha }_\varepsilon$, ${\alpha }_\varepsilon$ have the same laws on $\mathcal {Z}_T$, thus

138 $$\begin{aligned} \mathcal {L}(\widehat{\alpha }_\varepsilon )\left( \mathcal {C}([0,T]; \mathrm { H})\right) = 1, \quad \varepsilon > 0, \end{aligned}$$

and from estimates (95) and (114), for $p \in [2, \infty )$

139 $$\begin{aligned} \sup _{\varepsilon > 0} \widehat{\mathbb {E}} \left( \sup _{0 \le s \le T} \Vert \widehat{\alpha }_\varepsilon (s)\Vert ^{p}_{{\mathbb {L}^2({\mathbb {S}^2})}} \right) \le K_1(p). \end{aligned}$$

Since $L^2(0,T; \mathrm {V}) \cap \mathcal {Z}_T$ is a Borel subset of $\mathcal {Z}_T$ (Lemma 25), ${\alpha }_\varepsilon$ and $\widehat{\alpha }_\varepsilon$ have same laws on $\mathcal {Z}_T$; from (95), we have

140 $$\begin{aligned} \sup _{\varepsilon > 0} \widehat{\mathbb {E}} \left[ \int _0^T \Vert \mathrm{{curl}^\prime }\widehat{\alpha }_\varepsilon (s)\Vert ^2_{{\mathbb {L}^2({\mathbb {S}^2})}}\,ds \right] \le K_2. \end{aligned}$$

Since the laws of $\eta _{k_n}$ and $\widehat{\eta }_{k_n}$ are equal on $\mathcal {Y}_T$, we infer that the corresponding marginal laws are also equal. In other words, the laws on $\mathcal {B}\left( \mathcal {Y}_T^{\varepsilon _{k_n}}\right)$ of $\mathcal {L}(\widehat{\beta }_{\varepsilon _{k_n}})$ and $\mathcal {L}(\widetilde{\beta }_{\varepsilon _{k_n}})$ are equal for every ${k_n}$.

Therefore, from the estimates (102) and (115) we infer for $p \in [2, \infty )$

141 $$\begin{aligned} \widehat{\mathbb {E}} \left( \sup _{0 \le s \le T} \Vert \widehat{\beta }_\varepsilon (s)\Vert ^{p}_{{\mathbb {L}^2(Q_\varepsilon )}} \right) \le K_3(p) \varepsilon ^{p/2}, \qquad \varepsilon \in (0,1] \end{aligned}$$

and

142 $$\begin{aligned} \widehat{\mathbb {E}} \left[ \int _0^T \Vert \mathrm{curl}\;\widehat{\beta }_\varepsilon (s)\Vert ^2_{{\mathbb {L}^2(Q_\varepsilon )}}\,ds \right] \le K_4 \varepsilon , \qquad \varepsilon \in (0,1]. \end{aligned}$$

By inequality (140) we infer that the sequence $(\widehat{\alpha }_\varepsilon )_{\varepsilon > 0}$ contains a subsequence, still denoted by $(\widehat{\alpha }_\varepsilon )_{\varepsilon > 0}$ convergent weakly (along the sequence $\varepsilon _{k_n}$) in the space $L^2([0,T] \times \widehat{\varOmega }; \mathrm { V})$. Since $\widehat{\alpha }_\varepsilon \rightarrow \widehat{u}$ in $\mathcal {Z}_T$ $\widehat{\mathbb {P}}$-a.s., we conclude that $\widehat{u}\in L^2([0,T] \times \widehat{\varOmega }; \mathrm { V})$, i.e.

143 $$\begin{aligned} \widehat{\mathbb {E}} \left[ \int _0^T \Vert \mathrm{{curl}^\prime }\widehat{u}(s)\Vert ^2_{{\mathbb {L}^2({\mathbb {S}^2})}}\,ds \right] < \infty . \end{aligned}$$

Similarly by inequality (139), for every $p \in [2, \infty )$ we can choose a subsequence of $(\widehat{\alpha }_\varepsilon )_{\varepsilon >0}$ convergent weak star (along the sequence $\varepsilon _{k_n}$) in the space $L^p(\widehat{\varOmega }; L^\infty (0,T; \mathrm { H}))$ and, using (134), we infer that

144 $$\begin{aligned} \widehat{\mathbb {E}} \left( \sup _{0 \le s \le T} \Vert \widehat{u}(s)\Vert ^{p}_{{\mathbb {L}^2({\mathbb {S}^2})}} \right) < \infty . \end{aligned}$$

Using the convergence from (134) and estimates (139)–(144) we will prove certain term-by-term convergences which will be used later to prove Theorem 3. In order to simplify the notation, in the result below we write $\lim _{\varepsilon \rightarrow 0}$ but we mean $\lim _{k_n \rightarrow \infty }$.

Before stating the next lemma, we introduce a new functional space $\mathbb {U}$ as the space of compactly supported, smooth divergence free vector fields on ${\mathbb {S}^2}$:

145 $$\begin{aligned} \mathbb {U}:= \{ {\mathrm {v}}:= (0,{\mathrm {v}}_\lambda , {\mathrm {v}}_\varphi ) \in C^\infty _c({\mathbb {S}^2};\mathbb {R}^3) : \mathrm{{div}^\prime }{\mathrm {v}}= 0 \text{ in } {\mathbb {S}^2}\}. \end{aligned}$$

#### Lemma 26

For all $t \in [0,T]$, and $\phi \in \mathbb {U},$ we have (along the sequence $\varepsilon _{k_n}$)

- $$\lim _{\varepsilon \rightarrow 0} \widehat{\mathbb {E}}\left[ \int _0^T \left| \left( \widehat{\alpha }_\varepsilon (t) - \widehat{u}(t),\phi \right) _{\mathbb {L}^2({\mathbb {S}^2})}\right| \,dt\right] = 0$$,
- $$\lim _{\varepsilon \rightarrow 0} \;\left( \widehat{\alpha }_\varepsilon (0) - u_0,\phi \right) _{\mathbb {L}^2({\mathbb {S}^2})}= 0$$,
- $$\lim _{\varepsilon \rightarrow 0} \widehat{\mathbb {E}}\left[ \int _0^T \left| \int _0^t \left( \dfrac{\nu }{1+\varepsilon } \mathrm{{curl}^\prime }\widehat{\alpha }_\varepsilon (s) - \mathrm{{curl}^\prime }\widehat{u}(s), \mathrm{curl}\;\phi \right) _{\mathbb {L}^2({\mathbb {S}^2})}ds\right| \,dt\right] = 0$$,
- $$\lim _{\varepsilon \rightarrow 0} \widehat{\mathbb {E}}\left[ \int _0^T \left| \int _0^t \left( \dfrac{1}{1{+}\varepsilon } \left[ \widehat{\alpha }_\varepsilon (s) \cdot \nabla ^\prime \right] \widehat{\alpha }_\varepsilon (s) {-} \left[ \widehat{u}(s) \cdot \nabla ^\prime \right] \widehat{u}(s), \phi \right) _{\mathbb {L}^2({\mathbb {S}^2})}ds\right| \,dt\right] = 0$$,

#### Proof

Let us fix $\phi \in \mathbb {U}$.

**(a)** We know that $\widehat{\alpha }_\varepsilon \rightarrow \widehat{u}$ in $\mathcal {Z}_T$. In particular,

$$\begin{aligned} \widehat{\alpha }_\varepsilon \rightarrow \widehat{u} \quad \text { in }\, \mathcal {C}([0,T]; \mathrm { H}_w)\quad \widehat{\mathbb {P}}\text {-a.s.} \end{aligned}$$

Hence, for $$t \in [0,T]$$

146 $$\begin{aligned} \lim _{\varepsilon \rightarrow 0} \left( \widehat{\alpha }_\varepsilon (t),\phi \right) _{\mathbb {L}^2({\mathbb {S}^2})}= \left( \widehat{u}(t), \phi \right) _{\mathbb {L}^2({\mathbb {S}^2})}, \quad \widehat{\mathbb {P}}\text {-a.s.} \end{aligned}$$

Since by (139), for every $\varepsilon > 0$, $\sup _{t\in [0,T]}\Vert \widehat{\alpha }_\varepsilon (s)\Vert ^2_{\mathbb {L}^2({\mathbb {S}^2})}\le K_1(2)$, $\widehat{\mathbb {P}}$-a.s., using the dominated convergence theorem we infer that

147 $$\begin{aligned} \lim _{\varepsilon \rightarrow 0} \int _0^T \left| \left( \widehat{\alpha }_\varepsilon (t) - \widehat{u}(t), \phi \right) _{\mathbb {L}^2({\mathbb {S}^2})}\right| dt = 0,\quad \widehat{\mathbb {P}}\text {-a.s.} \end{aligned}$$

By the Hölder inequality, (139) and (144) for every $\varepsilon > 0$ and every $r > 1$

148 $$\begin{aligned} \begin{aligned}&\widehat{\mathbb {E}}\left[ \left| \int _0^T \Vert \widehat{\alpha }_\varepsilon (t) - \widehat{u}(t)\Vert _{\mathbb {L}^2({\mathbb {S}^2})}dt\right| ^r\right] \\&\qquad \le c \widehat{\mathbb {E}}\left[ \int _0^T\left( \Vert \widehat{\alpha }_\varepsilon (t)\Vert _{\mathbb {L}^2({\mathbb {S}^2})}^{r} + \Vert \widehat{u}(t)\Vert ^{r}_{\mathbb {L}^2({\mathbb {S}^2})}\right) dt \right] \le cTK_1(r), \end{aligned} \end{aligned}$$

where *c* is some positive constant. To conclude the proof of assertion (*a*) it is sufficient to use (147), (148) and the Vitali’s convergence theorem.

**(b)** Since $\widehat{\alpha }_\varepsilon \rightarrow \widehat{u}$ in $\mathcal {C}([0,T]; \mathrm { H}_w)$ $\widehat{\mathbb {P}}$-a.s. we infer that

149 $$\begin{aligned} \left( \widehat{\alpha }_\varepsilon (0),\phi \right) _{\mathbb {L}^2({\mathbb {S}^2})}\rightarrow \left( \widehat{u}(0),\phi \right) _{\mathbb {L}^2({\mathbb {S}^2})}, \quad \widehat{\mathbb {P}}\text {-a.s.} \end{aligned}$$

Also, note that by condition (87) in Assumption 2,  converges weakly to $u_0$ in $\mathbb {L}^2({\mathbb {S}^2})$.

On the other hand, by (133) we infer that the laws of $\widehat{\alpha }_\varepsilon (0)$ and $\alpha _\varepsilon (0)$ on $\mathrm { H}$ are equal. Since $\alpha _\varepsilon (0)$ is a constant random variable on the old probability space, we infer that $\widehat{\alpha }_\varepsilon (0)$ is also a constant random variable (on the new probability space) and hence, by (72) and (94), we infer that  almost surely (on the new probability space). Therefore we infer that

$$\begin{aligned} \left( \widehat{u}(0), \phi \right) _{{\mathbb {L}^2({\mathbb {S}^2})}} = \left( u_0, \phi \right) _{{\mathbb {L}^2({\mathbb {S}^2})}}, \end{aligned}$$

concluding the proof of assertion (*b*).

**(c)** Since $$\widehat{\alpha }_\varepsilon \rightarrow \widehat{u}$$ in $$\mathcal {C}([0,T]; \mathrm { H}_w)$$ $$\widehat{\mathbb {P}}$$-a.s.,

150 $$\begin{aligned} \lim _{\varepsilon \rightarrow 0}\frac{\nu }{1+\varepsilon } \int _0^t \left( \mathrm{{curl}^\prime }\widehat{\alpha }_\varepsilon (s), \mathrm{{curl}^\prime }\phi \right) _{\mathbb {L}^2({\mathbb {S}^2})}\,ds&= - \lim _{\varepsilon \rightarrow 0} \frac{\nu }{1+\varepsilon } \int _0^t \left( \widehat{\alpha }_\varepsilon (s), \varvec{{\Delta }}^\prime \, \phi \right) _{\mathbb {L}^2({\mathbb {S}^2})}\,ds \nonumber \\ = - \nu \int _0^t \left( \widehat{u}, \varvec{{\Delta }}^\prime \, \phi \right) _{\mathbb {L}^2({\mathbb {S}^2})}\,ds&= \nu \int _0^t \left( \mathrm{{curl}^\prime }\widehat{u}, \mathrm{{curl}^\prime }\phi \right) _{\mathbb {L}^2({\mathbb {S}^2})}\,ds. \end{aligned}$$

The Cauchy–Schwarz inequality and estimate (140) infer that for all $t \in [0,T]$ and $\varepsilon \in (0,1]$

151 $$\begin{aligned} \widehat{\mathbb {E}}&\left[ \left| \int _0^t \left( \dfrac{\nu }{1+\varepsilon }\mathrm{{curl}^\prime }\widehat{\alpha }_\varepsilon (s), \mathrm{{curl}^\prime }\phi \right) _{\mathbb {L}^2({\mathbb {S}^2})}\,ds\right| ^2\right] \nonumber \\&\quad \le \nu ^2 \Vert \mathrm{{curl}^\prime }\phi \Vert _{\mathbb {L}^2({\mathbb {S}^2})}^2 \widehat{\mathbb {E}}\left[ \int _0^t \Vert \mathrm{{curl}^\prime }\widehat{\alpha }_\varepsilon (s)\Vert ^2_{\mathbb {L}^2({\mathbb {S}^2})}\,ds\right] \le c K_2\, \end{aligned}$$

for some constant $c > 0$. By (150), (151) and the Vitali’s convergence theorem we conclude that for all $t \in [0,T]$

$$\begin{aligned} \lim _{\varepsilon \rightarrow 0} \widehat{\mathbb {E}}\left[ \left| \int _0^t \nu \left( \dfrac{1}{1+\varepsilon } \mathrm{{curl}^\prime }\widehat{\alpha }_\varepsilon (s) - \mathrm{{curl}^\prime }\widehat{u}(s), \mathrm{{curl}^\prime }\phi \right) _{\mathbb {L}^2({\mathbb {S}^2})}\,ds\right| \right] = 0 . \end{aligned}$$

Assertion (*c*) follows now from (140), (143) and the dominated convergence theorem.

**(d)** For the non-linear term, using the Sobolev embedding $\mathbb {H}^2({\mathbb {S}^2}) \hookrightarrow \mathbb {L}^\infty ({\mathbb {S}^2})$, we have

152 $$\begin{aligned}&\left| \int _0^{t} \left( \left[ \widehat{\alpha }_\varepsilon (s) \cdot \nabla ^\prime \right] \widehat{\alpha }_\varepsilon (s), \phi \right) _{\mathbb {L}^2({\mathbb {S}^2})}\,ds - \int _0^t \left( \left[ \widehat{u}(s) \cdot \nabla ^\prime \right] \widehat{u}(s), \phi \right) _{\mathbb {L}^2({\mathbb {S}^2})}\,ds\right| \nonumber \\&\le \left| \int _0^t \int _{\mathbb {S}^2}\left[ \left( \widehat{\alpha }_\varepsilon (s,x) - \widehat{u}(s,x) \right) \cdot \nabla ^\prime \widehat{u}(s,x) \right] \cdot \phi (x) \,dx\,ds\right| \nonumber \\&\quad + \left| \int _0^t \int _{\mathbb {S}^2}\left[ \widehat{\alpha }_\varepsilon (s,x) \cdot \nabla ^\prime \left( \widehat{\alpha }_\varepsilon (s,x) - \widehat{u}(s,x)\right) \right] \cdot \phi (x) \,dx\,ds\right| \nonumber \\&\le \Vert \widehat{\alpha }_\varepsilon - \widehat{u}\Vert _{L^2(0,T; \mathrm { H})}\Vert \widehat{u}\Vert _{L^2(0,T; \mathrm { V})}\Vert \phi \Vert _{\mathbb {H}^2({\mathbb {S}^2})} \nonumber \\&\quad + \left| \int _0^t \left( \nabla ^\prime \left( \widehat{\alpha }_\varepsilon (s,x) - \widehat{u}(s,x)\right) , \widehat{\alpha }_\varepsilon (s)\phi \right) _{\mathbb {L}^2({\mathbb {S}^2})}ds\right| . \end{aligned}$$

The first term converges to zero as $\varepsilon \rightarrow 0$, since $\widehat{\alpha }_\varepsilon \rightarrow \widehat{u}$ strongly in $L^2(0,T; \mathrm { H})$ $\widehat{\mathbb {P}}$-a.s., $\widehat{u} \in L^2(0,T; \mathrm { V})$ and the second term converges to zero too as $\varepsilon \rightarrow 0$ because $\widehat{\alpha }_\varepsilon \rightarrow \widehat{u}$ weakly in $L^2(0,T; \mathrm { V})$. Using the Hölder inequality, estimates (139) and the embedding $\mathbb {H}^2({\mathbb {S}^2}) \hookrightarrow \mathbb {L}^\infty ({\mathbb {S}^2})$ we infer that for all $t \in [0,T]$, $\varepsilon \in (0,1]$, the following inequalities hold

153 $$\begin{aligned} \widehat{\mathbb {E}}&\left[ \left| \int _0^t \tfrac{1}{1+ \varepsilon }\left( \left[ \widehat{\alpha }_\varepsilon (s)\cdot \nabla ^\prime \right] \widehat{\alpha }_\varepsilon (s),\phi \right) _{\mathbb {L}^2({\mathbb {S}^2})}ds\right| ^2\right] \nonumber \\&\le \widehat{\mathbb {E}}\left[ \left( \int _0^t\Vert \widehat{\alpha }_\varepsilon (s)\Vert _{\mathbb {L}^2({\mathbb {S}^2})}^2 \Vert \nabla ^\prime \phi \Vert _{\mathbb {L}^\infty ({\mathbb {S}^2})}\,ds\right) ^2\right] \nonumber \\&\le c \Vert \nabla ^\prime \phi \Vert _{\mathbb {H}^2({\mathbb {S}^2})}\,t\, \widehat{\mathbb {E}}\left[ \int _0^t \Vert \widehat{\alpha }_\varepsilon (s)\Vert ^4_{\mathbb {L}^2({\mathbb {S}^2})}\,ds\right] \nonumber \\&\le c \Vert \phi \Vert _{\mathbb {H}^3({\mathbb {S}^2})}\, t\, \widehat{\mathbb {E}}\left[ \sup _{s \in [0,t]}\Vert \widehat{\alpha }_\varepsilon (s)\Vert ^4_{\mathbb {L}^2({\mathbb {S}^2})}\right] \le \widetilde{c} K_1(4). \end{aligned}$$

By (152), (153) and the Vitali’s convergence theorem we obtain for all $t \in [0,T]$,

154 $$\begin{aligned} \lim _{\varepsilon \rightarrow 0} \widehat{\mathbb {E}}\left[ \left| \int _0^t \left( \frac{1}{1+\varepsilon } \left[ \widehat{\alpha }_\varepsilon (s)\cdot \nabla ^\prime \right] \widehat{\alpha }_\varepsilon (s) - \left[ \widehat{u}(s)\cdot \nabla ^\prime \right] \widehat{u}(s), \phi \right) _{\mathbb {L}^2({\mathbb {S}^2})}\,ds\right| \right] = 0. \end{aligned}$$

Using the Hölder inequality and estimates (139), (144), we obtain for all $t \in [0,T]$, $\varepsilon \in (0,1]$

$$\begin{aligned}&\widehat{\mathbb {E}}\left[ \left| \int _0^t \frac{1}{1+\varepsilon }\left( \left[ \widehat{\alpha }_\varepsilon (s)\cdot \nabla ^\prime \right] \widehat{\alpha }_\varepsilon (s) - \left[ \widehat{u}(s) \cdot \nabla ^\prime \right] \widehat{u}(s), \phi \right) _{\mathbb {L}^2({\mathbb {S}^2})}\,ds\right| \right] \\&\quad \le c \Vert \phi \Vert _{\mathbb {H}^3({\mathbb {S}^2})}\widehat{\mathbb {E}}\left[ \sup _{t \in [0,T]}\Vert \widehat{\alpha }_\varepsilon (s)\Vert ^2_{\mathbb {L}^2({\mathbb {S}^2})}+ \sup _{t \in [0,T]}\Vert \widehat{u}(s)\Vert ^2_{\mathbb {L}^2({\mathbb {S}^2})}\right] \le 2\widetilde{c} K_1(2) \end{aligned}$$

where $c, \widetilde{c} > 0$ are constants. Hence by (154) and the dominated convergence theorem, we infer assertion (*d*).

**(e)** Assertion (*e*) follows because by Assumption 2 the sequence  converges weakly in $L^2(0,T; {\mathbb {L}^2({\mathbb {S}^2})})$ to *f*.

**(f)** By the definition of maps $\widetilde{G}_\varepsilon$ and *G*, we haveSince, by Assumption 2, for every $j \in \{1, \ldots , N\}$, and $s \in [0,t]$,  converges weakly to $g^j(s)$ in ${\mathbb {L}^2({\mathbb {S}^2})}$ as $\varepsilon \rightarrow 0$, we get155By assumptions on $\widetilde{g}_\varepsilon ^j$, we obtain the following inequalities for every $t \in [0,T]$ and $\varepsilon \in (0,1]$156where $c, \widetilde{c} > 0$ are some constants. Using the Vitali’s convergence theorem, by (155) and (156) we infer157Hence, by the properties of the Itô integral we deduce that for all $t \in [0,T]$,158By the Itô isometry and assumptions on $\widetilde{g}_\varepsilon ^j$ and $g^j$ we have for all $t \in [0,T]$ and $\varepsilon \in (0,1]$159where $c > 0$ is a constant. Thus, by (158), (159) and the dominated convergence theorem assertion (*f*) holds. $\square$

#### Lemma 27

For all $t \in [0,T]$ and $\phi \in \mathbb {U}$, we have (along the sequence $\varepsilon _{k_n}$)

where the process $\underline{\widehat{\alpha }_\varepsilon }$ is defined in (136).

#### Proof

Let us fix $\phi \in \mathbb {U}$.

**(a)** Let $t \in [0,T]$, then by the Hölder inequality, Lemma 18, Poincaré inequality and estimate (142), we have the following inequalitiesThus160We infer assertion (*a*) by dominated convergence theorem, estimate (142) and convergence (160).

**(b)** Using the Hölder inequality, scaling property (Lemma 11), Corollary 4, relations (67), (68), and estimates (139), (142), we get for $t \in [0,T]$Thus161We infer assertion (*b*) by dominated convergence theorem, estimates (139), (142) and convergence (161). Assertion (*c*) can be proved similarly.

**(d)** Now for the last one, using the Hölder inequality, Corollary 4, relations (67), (68), and estimates (141), (142), we get for $t \in [0,T]$Thus162We infer assertion (*d*) by dominated convergence theorem, estimates (141), (142) and convergence (162). $\square$

Finally, to finish the proof of Theorem 3, we will follow the methodology as in [42] and introduce some auxiliary notations (along sequence $\varepsilon _{k_n}$)163

164 $$\begin{aligned}&\varLambda (\widehat{u},\widehat{W}, \phi ) := \left( u_0, \phi \right) _{\mathbb {L}^2({\mathbb {S}^2})}-\nu \int _0^t \left( \mathrm{{curl}^\prime }\widehat{u}(s), \mathrm{{curl}^\prime }\phi \right) _{\mathbb {L}^2({\mathbb {S}^2})}\,ds \nonumber \\&\quad - \int _0^{t} \left( \left[ \widehat{u}(s) \cdot \nabla ^\prime \right] \widehat{u}(s), \phi \right) _{\mathbb {L}^2({\mathbb {S}^2})}\,ds + \int _0^{t} \left( f(s), \phi \right) _{\mathbb {L}^2({\mathbb {S}^2})}\,ds \nonumber \\&\quad + \int _0^t \left( G(s) d \widehat{W}(s), \phi \right) _{\mathbb {L}^2({\mathbb {S}^2})}. \end{aligned}$$

#### Corollary 5

Let $\phi \in \mathbb {U}$. Then (along the sequence $\varepsilon _{k_n}$)

165 $$\begin{aligned} \lim _{\varepsilon \rightarrow 0} \left\| \left( \widehat{\alpha }_\varepsilon (\cdot ), \phi \right) _{\mathbb {L}^2({\mathbb {S}^2})}- \left( \widehat{u}(\cdot ), \phi \right) _{\mathbb {L}^2({\mathbb {S}^2})}\right\| _{L^1(\widehat{\varOmega } \times [0,T])} = 0 \end{aligned}$$

and

166 $$\begin{aligned} \lim _{\varepsilon \rightarrow 0} \left\| \varLambda _\varepsilon (\widehat{\alpha }_\varepsilon , \widehat{\beta }_\varepsilon , \widehat{W}_\varepsilon , \phi ) - \varLambda (\widehat{u}, \widehat{W}, \phi )\right\| _{L^1(\widehat{\varOmega } \times [0,T])} = 0. \end{aligned}$$

#### Proof

Assertion (165) follows from the equality

$$\begin{aligned} \left\| \left( \widehat{\alpha }_\varepsilon (\cdot ), \phi \right) _{\mathbb {L}^2({\mathbb {S}^2})}- \left( \widehat{u}(\cdot ), \phi \right) _{\mathbb {L}^2({\mathbb {S}^2})}\right\| _{L^1(\widehat{\varOmega } \times [0,T])} {=} \widehat{\mathbb {E}}\left[ \int _0^T \left| \left( \widehat{\alpha }_\varepsilon (t) {-} \widehat{u}(t), \phi \right) _{\mathbb {L}^2({\mathbb {S}^2})}\right| \,dt\right] \end{aligned}$$

and Lemma 26 (*a*). To prove assertion (166), note that by the Fubini Theorem, we have

$$\begin{aligned}&\left\| \varLambda _\varepsilon (\widehat{\alpha }_\varepsilon , \widehat{\beta }_\varepsilon , \widehat{W}_\varepsilon , \phi ) - \varLambda (\widehat{u}, \widehat{W}, \phi )\right\| _{L^1(\widehat{\varOmega } \times [0,T])} \\&\quad = \int _0^T \widehat{\mathbb {E}}\left| \varLambda _\varepsilon (\widehat{\alpha }_\varepsilon , \widehat{\beta }_\varepsilon , \widehat{W}_\varepsilon , \phi )(t) - \varLambda (\widehat{u}, \widehat{W}, \phi )(t)\right| dt. \end{aligned}$$

To conclude the proof of the corollary, it is sufficient to note that by Lemma 26$(b)-(f)$ and Lemma 27, each term on the right hand side of (163) tends at least in $L^1(\widehat{\varOmega } \times [0,T])$ to the corresponding term (to zero in certain cases) in (164). $\square$

#### Conclusion of proof of Theorem 3

Let us fix $\phi \in \mathbb {U}$. Since ${\alpha }_\varepsilon$ is a solution of (85), for all $t \in [0,T]$,

$$\begin{aligned} \left( {\alpha }_\varepsilon (t), \phi \right) _{\mathbb {L}^2({\mathbb {S}^2})}= \varLambda _\varepsilon ({\alpha }_\varepsilon , \widetilde{\beta }_\varepsilon , \widetilde{W}_\varepsilon , \phi )(t), \qquad \mathbb {P}\text {-a.s.} \end{aligned}$$

In particular,

$$\begin{aligned} \int _0^T \mathbb {E}\left[ \left| \left( {\alpha }_\varepsilon (t),\phi \right) _{\mathbb {L}^2({\mathbb {S}^2})}- \varLambda _\varepsilon ({\alpha }_\varepsilon , \widetilde{\beta }_\varepsilon , \widetilde{W}_\varepsilon , \phi )(t)\right| \right] \,dt = 0. \end{aligned}$$

Since $\mathcal {L}({\alpha }_\varepsilon , \eta _{k_n}, \widetilde{W}_\varepsilon ) = \mathcal {L}(\widehat{\alpha }_\varepsilon , \widehat{\eta }_{k_n}, \widehat{W}_\varepsilon )$, on $\mathcal {B}\left( \mathcal {Z}_T \times \mathcal {Y}_T \times \mathcal {C}([0,T]; \mathbb {R}^N)\right)$ (along the sequence $\varepsilon _{k_n}$),

$$\begin{aligned} \int _0^T \widehat{\mathbb {E}}\left[ \left| \left( \widehat{\alpha }_\varepsilon (t),\phi \right) _{\mathbb {L}^2({\mathbb {S}^2})}- \varLambda _\varepsilon (\widehat{\alpha }_\varepsilon , \widehat{\beta }_\varepsilon , \widehat{W}_\varepsilon , \phi )(t)\right| \right] \,dt = 0. \end{aligned}$$

Therefore by Corollary 5 and the definition of $\varLambda$, for almost all $t \in [0,T]$ and $\widehat{\mathbb {P}}$-almost all $\omega \in \widehat{\varOmega }$

$$\begin{aligned} \left( \widehat{u}(t), \phi \right) _{\mathbb {L}^2({\mathbb {S}^2})}- \varLambda (\widehat{u}, \widehat{W}, \phi )(t) = 0, \end{aligned}$$

i.e. for almost all $t \in [0,T]$ and $\widehat{\mathbb {P}}$-almost all $\omega \in \widehat{\varOmega }$

167 $$\begin{aligned} \left( \widehat{u}(t), \phi \right) _{\mathbb {L}^2({\mathbb {S}^2})}+ \nu \int _0^t \left( \mathrm{{curl}^\prime }\widehat{u}(s), \mathrm{{curl}^\prime }\phi \right) _{\mathbb {L}^2({\mathbb {S}^2})}\,ds + \int _0^{t} \left( \left[ \widehat{u}(s) \cdot \nabla ^\prime \right] \widehat{u}(s), \phi \right) _{\mathbb {L}^2({\mathbb {S}^2})}\,ds \nonumber \\ = \left( u_0, \phi \right) _{\mathbb {L}^2({\mathbb {S}^2})}+ \int _0^{t} \left( f(s), \phi \right) _{\mathbb {L}^2({\mathbb {S}^2})}\,ds + \left( \int _0^t G(s) d \widehat{W}(s), \phi \right) _{\mathbb {L}^2({\mathbb {S}^2})}. \end{aligned}$$

Hence (167) holds for every $\phi \in \mathbb {U}$. Since $\widehat{u}$ is a.s. $\mathrm { H}$-valued continuous process, by a standard density argument, we infer that (167) holds for every $\phi \in \mathrm { V}$ ($\mathbb {U}$ is dense in $\mathrm { V}$).

Putting $\widehat{\mathcal {U}} := \left( \widehat{\varOmega }, \widehat{\mathcal {F}}, \widehat{\mathbb {F}}, \widehat{\mathbb {P}}\right)$, we infer that the system $\left( \widehat{\mathcal {U}}, \widehat{W}, \widehat{u}\right)$ is a martingale solution to (75)–(77). $\square$

## Vector Analysis in Spherical Coordinates

In this appendix we collect some basic results from vector algebra and formulas for Laplace and gradient of scalar function and vector fields in spherical coordinates.

The following identities are very well known [53, Appendix] in vector algebra$:$ Let ${\mathrm {u}}, {\mathrm {v}}$ and ${\mathrm {w}}$ be $\mathbb {R}^3$-valued smooth vector fields then

168 $$\begin{aligned} \mathrm{curl}\;\mathrm{curl}\;{\mathrm {u}}&= - \varDelta {\mathrm {u}}+ \nabla \mathrm{div}\;{\mathrm {u}}, \end{aligned}$$

169 $$\begin{aligned} {\mathrm {u}}\cdot \left( {\mathrm {v}}\times {\mathrm {w}}\right)&= \left( {\mathrm {u}}\times {\mathrm {v}}\right) \cdot {\mathrm {w}}, \end{aligned}$$

170 $$\begin{aligned} \int _{Q_\varepsilon } {\mathrm {v}}\cdot \mathrm{curl}\;{\mathrm {u}}\, d\mathbf{y}&= \int _{Q_\varepsilon } {\mathrm {u}}\cdot \mathrm{curl}\;{\mathrm {v}}\, d\mathbf{y}+ \int _{\partial Q_\varepsilon } \left( {\mathrm {u}}\times {\mathrm {v}}\right) \cdot \mathbf {n}\,d\sigma . \end{aligned}$$

The Laplace–Beltrami operator of a scalar function $\psi$, in spherical coordinates $(r, \lambda , \varphi )$ is given by

171 $$\begin{aligned} \nabla ^2 \psi = \frac{\partial ^2 \psi }{\partial r^2} + \frac{2}{r} \frac{\partial \psi }{\partial r} + \frac{1}{r^2 \sin \lambda }\frac{\partial }{\partial \lambda }\left( \sin \lambda \frac{\partial \psi }{\partial \lambda }\right) + \frac{1}{r^2 \sin ^2 \lambda }\frac{\partial ^2\psi }{\partial \varphi ^2}, \end{aligned}$$

and its gradient is given by

172 $$\begin{aligned} \nabla \psi = \frac{\partial \psi }{\partial r}\widehat{e_r} + \frac{1}{r}\frac{\partial \psi }{\partial \lambda }\widehat{e_\lambda } + \frac{1}{r \sin \lambda }\frac{\partial \psi }{\partial \varphi }\widehat{e_\varphi }. \end{aligned}$$

For a vector field *u* written in the spherical coordinates, $u = u_r \widehat{e_r} + u_\lambda \widehat{e_\lambda } + u_\varphi \widehat{e_\varphi }$, the curl and the divergence are given as follows

173 $$\begin{aligned} \mathrm{curl}\;u&= \frac{1}{r \sin \lambda } \left[ \frac{\partial }{\partial \lambda }(\sin \lambda u_\varphi ) - \frac{\partial u_\lambda }{\partial \varphi } \right] \widehat{e_r} + \left[ \frac{1}{r \sin \lambda }\frac{\partial u_r}{\partial \varphi } - \frac{1}{r}\frac{\partial }{\partial r}(r u_\varphi )\right] \widehat{e_\lambda } \nonumber \\&\qquad + \left[ \frac{1}{r}\frac{\partial }{\partial r}(r u_\lambda ) - \frac{1}{r}\frac{\partial u_r}{\partial \lambda }\right] \widehat{e_\varphi }, \end{aligned}$$

174 $$\begin{aligned} \mathrm{div}\;u&= \frac{1}{r^2}\frac{\partial }{\partial r}(r^2 u_r) + \frac{1}{r \sin \lambda } \frac{\partial }{\partial \lambda }(u_\lambda \sin \lambda ) + \frac{1}{r \sin \lambda }\frac{\partial u_\varphi }{\partial \varphi }. \end{aligned}$$

The Laplacian of a vector field in spherical coordinates is

175 $$\begin{aligned} \begin{aligned} \left( \varDelta u\right) _r&= \nabla ^2 u_r -\frac{2u_r}{r^2} - \frac{2}{r^2}\frac{\partial u_\lambda }{\partial \lambda } - 2 \frac{\cot \lambda }{r^2} u_\lambda - \frac{2}{r^2 \sin \lambda } \frac{\partial u_\varphi }{\partial \varphi },\\ \left( \varDelta u\right) _\lambda&= \nabla ^2 u_\lambda + \frac{2}{r^2}\frac{\partial u_r}{\partial \lambda } - \frac{u_\lambda }{r^2 \sin ^2 \lambda } - \frac{2 \cos \lambda }{r^2 \sin ^2 \lambda } \frac{\partial u_\varphi }{\partial \varphi }, \\ \left( \varDelta u\right) _\varphi&= \nabla ^2 u_\varphi - \frac{u_\varphi }{r^2 \sin ^2 \lambda } + \frac{1}{r^2 \sin \lambda }\frac{\partial u_r}{\partial \varphi } + \frac{2 \cos \lambda }{r^2 \sin ^2 \lambda } \frac{\partial u_\lambda }{\partial \varphi }, \end{aligned} \end{aligned}$$

where $\nabla ^2 u_r$, $\nabla ^2 u_\lambda$ and $\nabla ^2 u_\varphi$ are as in (171).

We recall some standard differential operators on the unit sphere ${\mathbb {S}^2}$. For $\psi$ a scalar function defined on ${\mathbb {S}^2}$, the tangential gradient is given by

176 $$\begin{aligned} \nabla ^\prime \psi = \frac{\partial \psi }{\partial \lambda } \widehat{e_\lambda } + \frac{1}{\sin \lambda } \frac{\partial \psi }{\partial \varphi }\widehat{e_\varphi }. \end{aligned}$$

The Laplace–Beltrami of a scalar function $\psi$ is

177 $$\begin{aligned} \varDelta ^\prime \psi = \frac{1}{\sin {\lambda }} \left[ \frac{\partial }{\partial \lambda }\left( \sin {\lambda } \frac{\partial \psi }{\partial \lambda }\right) + \frac{1}{\sin \lambda } \frac{\partial ^2\psi }{\partial \varphi ^2}\right] . \end{aligned}$$

For a tangential vector field ${\mathrm {v}}$ defined on ${\mathbb {S}^2}$, ${\mathrm {v}}= {\mathrm {v}}_\lambda \hat{e}_\lambda + {\mathrm {v}}_\varphi \hat{e}_\varphi$, the tangential divergence is expressed by

178 $$\begin{aligned} \mathrm{{div}}^\prime {\mathrm {v}}= \frac{1}{\sin {\lambda }}\frac{\partial }{\partial \lambda }\left( {\mathrm {v}}_\lambda \sin {\lambda }\right) + \frac{1}{\sin \lambda } \frac{\partial {\mathrm {v}}_\varphi }{\partial \varphi }, \end{aligned}$$

and the $\mathrm{{curl}}^\prime {\mathrm {v}}$ is the scalar function defined by

179 $$\begin{aligned} \mathrm{{curl}}^\prime {\mathrm {v}}= \frac{1}{\sin {\lambda }}\frac{\partial }{\partial \lambda }\left( {\mathrm {v}}_\varphi \sin {\lambda }\right) - \frac{1}{\sin \lambda } \frac{\partial {\mathrm {v}}_\lambda }{\partial \varphi }. \end{aligned}$$

The Laplace–de Rham operator applied to a vector field ${\mathrm {v}}$ is given by

180 $$\begin{aligned} \varvec{{\Delta }}^\prime \, {\mathrm {v}}= \left[ {\varDelta ^\prime }{\mathrm {v}}_\lambda - \frac{2 \cos {\lambda }}{\sin ^2{\lambda }} \frac{\partial {\mathrm {v}}_\varphi }{\partial \varphi } - \frac{{\mathrm {v}}_\lambda }{\sin ^2{\lambda }}\right] \widehat{e_\lambda } + \left[ {\varDelta ^\prime }{\mathrm {v}}_\varphi + \frac{2 \cos {\lambda }}{\sin ^2{\lambda }} \frac{\partial {\mathrm {v}}_\lambda }{\partial \varphi } - \frac{{\mathrm {v}}_\varphi }{\sin ^2{\lambda }}\right] \widehat{e_\varphi }, \end{aligned}$$

where ${\varDelta ^\prime }{\mathrm {v}}_\varphi$ and ${\varDelta ^\prime }{\mathrm {v}}_\lambda$ are as in (177).

## The curl and the Stokes operator

In this section we present a integration by parts formula corresponding to $\mathrm {curl}$ operator and later we use it to give a relation between the Stokes operator $\mathrm { A}_\varepsilon$ and $\mathrm{curl}\;$.

Let $\mathcal {O} \subset \mathbb {R}^3$ be a bounded domain with a regular boundary $\partial \mathcal {O}$. Define

181 $$\begin{aligned} \mathrm { H}(\mathrm {curl}) := & {} \{ u \in \mathbb {L}^2(\mathcal {O}) : \mathrm{curl}\;u \in \mathbb {L}^2(\mathcal {O}) \}. \end{aligned}$$

$\mathrm { H}(\mathrm {curl})$ with the graph norm

$$\begin{aligned} \Vert {\mathrm {v}}\Vert _{\mathrm { H}(\mathrm {curl})}^2 := \Vert {\mathrm {v}}\Vert ^2_{\mathbb {L}^2(\mathcal {O})} + \Vert \mathrm{curl}\;{\mathrm {v}}\Vert ^2_{\mathbb {L}^2(\mathcal {O})}, \quad {\mathrm {v}}\in \mathrm { H}(\mathrm {curl}), \end{aligned}$$

is a Hilbert space.

The following theorem is a reformulation of Lemma 4.2 from [17, p. 341].

### Theorem 4

Assume that $\mathcal {O} \subset \mathbb {R}^3$ be a bounded domain with a regular boundary and $\mathbf {n}$ be unit normal vector field on $\varGamma = \partial \mathcal {O}$ (directed towards exterior of $\mathcal {O}$). Then there exists a unique bounded linear map

182 $$\begin{aligned} n \times \cdot \big |_\varGamma :\mathrm { H}(\mathrm {curl}) \rightarrow \mathbb {H}^{-1/2}(\varGamma ), \end{aligned}$$

such that

183 $$\begin{aligned} \left( n \times \cdot \right) (u) = n \times u \big |_\varGamma , \end{aligned}$$

if $$u \in C^1_0(\overline{\mathcal {O}})$$ and

184 $$\begin{aligned} \int _\mathcal {O} \mathrm{curl}\;u \cdot {\mathrm {v}}\,dy - \int _\mathcal {O} u \cdot \mathrm{curl}\;{\mathrm {v}}\,dy = {}_{\mathbb {H}^{-1/2}(\varGamma )}\langle (n \times \cdot )(u) \big |_\varGamma , {\mathrm {v}}\big |_\varGamma \rangle _{\mathbb {H}^{1/2}(\varGamma )} \end{aligned}$$

for every $u \in \mathrm { H}(\mathrm {curl})$ and ${\mathrm {v}}\in \mathbb {H}^1(\mathcal {O})$.

### Remark 8

We will call formula (184) the generalised Stokes formula. From now on we will write $n \times u \big |_\varGamma$ instead of $(n \times \cdot )(u)\big |_\varGamma$ for $u \in \mathrm { H}(\mathrm {curl})$.

Recall that

185 $$\begin{aligned} \mathrm { H}= \{ u \in \mathbb {L}^2(\mathcal {O}) :\mathrm{div}\;u = 0\; \text{ in } \mathcal {O} \text{ and } u \cdot \mathbf {n} = 0 \text{ on } \varGamma \}, \end{aligned}$$

and the Stokes operator is given by

186 $$\begin{aligned} D(A)= & {} \{ u \in \mathbb {H}^2(\mathcal {O}) :\mathrm{div}\;u = 0 \text{ in } \mathcal {O},\; u \cdot \mathbf {n} = 0 \text{ and } \mathbf {n} \times \mathrm{curl}\;u = 0 \text{ on } \varGamma \},\nonumber \\ \end{aligned}$$

187 $$\begin{aligned} A u= & {} \pi (- \varDelta u), \quad u \in D(A). \end{aligned}$$

### Remark 9

To define $n \times \mathrm{curl}\;u$ as an element of $\mathbb {H}^{-1/2}(\varGamma )$, we need to know that $\mathrm{curl}\;(\mathrm{curl}\;u) \in \mathbb {L}^2(\mathcal {O})$. But if $u \in \mathbb {H}^2(\mathcal {O})$, then obviously this condition is satisfied.

### Theorem 5

*A* is self-adjoint and non-negative on $\mathrm { H}$.

### Proof

Here we will only show that *A* is symmetric and non-negative. Let $u, {\mathrm {v}}\in D(A)$, then

188 $$\begin{aligned} \left( Au, {\mathrm {v}}\right) _{\mathbb {L}^2(\mathcal {O})} = \left( \pi (-\varDelta u), {\mathrm {v}}\right) _{\mathbb {L}^2(\mathcal {O})} = - \left( \varDelta u, \pi {\mathrm {v}}\right) _{\mathbb {L}^2(\mathcal {O})} = \left( - \varDelta u, {\mathrm {v}}\right) _{\mathbb {L}^2(\mathcal {O})}. \end{aligned}$$

Recall that (from (168)) for smooth $\mathbb {R}^3$-valued vector fields,

$$\begin{aligned} \mathrm{curl}\;(\mathrm{curl}\;u) = - \varDelta u + \nabla (\mathrm{div}\;u). \end{aligned}$$

Using the above identity in (188) along with the fact that $u \in D(A)$, in particular, $\mathrm{div}\;u = 0$ and generalised Stokes formula (184), we have

$$\begin{aligned} \left( Au, {\mathrm {v}}\right) _{\mathbb {L}^2(\mathcal {O})}&= \int _\mathcal {O}\mathrm{curl}\;(\mathrm{curl}\;u) \cdot {\mathrm {v}}\,dy \\&= \int _\mathcal {O}\mathrm{curl}\;u \cdot \mathrm{curl}\;{\mathrm {v}}\,dy + {}_{\mathbb {H}^{-1/2}(\varGamma )}\langle \underbrace{\mathbf {n} \times (\mathrm{curl}\;u) \big |_\varGamma }_{=\,0, \text{ since } u \in D(A)} , {\mathrm {v}}\big |_\varGamma \rangle _{\mathbb {H}^{1/2}(\varGamma )} \\&= \int _\mathcal {O}\mathrm{curl}\;u \cdot \mathrm{curl}\;{\mathrm {v}}\,dy. \end{aligned}$$

Similarly

$$\begin{aligned} \left( u, A {\mathrm {v}}\right) _{\mathbb {L}^2(\mathcal {O})} = \left( A{\mathrm {v}}, u \right) _{\mathbb {L}^2(\mathcal {O})} = \int _\mathcal {O}\mathrm{curl}\;{\mathrm {v}}\cdot \mathrm{curl}\;u\,dy = \int _\mathcal {O}\mathrm{curl}\;u \cdot \mathrm{curl}\;{\mathrm {v}}\,dy. \end{aligned}$$

This establishes that *A* is symmetric on $\mathrm { H}$. The non-negativity follows from the above identity by taking ${\mathrm {v}}= u \in D(A)$. $\square$

Using the definition of *D*(*A*), we can characterise $D(A^{1/2})$ as

$$\begin{aligned} D(A^{1/2}) = \{u \in \mathbb {L}^2(\mathcal {O}) :\mathrm{div}\;u = 0,\, \mathrm{curl}\;u \in \mathbb {L}^2(\mathcal {O}) \text{ and } u \cdot \mathbf {n} = 0 \text{ on } \varGamma \}. \end{aligned}$$

By Theorem 6.1 [17, Pg 358] we have

$$\begin{aligned} D(A^{1/2}) \subset \{ u \in \mathbb {H}^1(\mathcal {O}) : u \cdot \mathbf {n} = 0 \text{ on } \varGamma \}. \end{aligned}$$

We use the following relation repeatedly in our calculations.

### Lemma 28

Let $u \in D(A^{1/2})$ and ${\mathrm {v}}\in D(A)$. Then

$$\begin{aligned} \left( \mathrm{curl}\;u, \mathrm{curl}\;{\mathrm {v}}\right) _{\mathbb {L}^2(\mathcal {O})} = \left( u, A {\mathrm {v}}\right) _{\mathbb {L}^2(\mathcal {O})}. \end{aligned}$$

### Proof

Note that for $u \in D(A^{1/2})$ and ${\mathrm {v}}\in D(A)$, the LHS makes sense. Using the generalised Stokes formula (184), we get

$$\begin{aligned} \int _\mathcal {O}\mathrm{curl}\;u \cdot \mathrm{curl}\;{\mathrm {v}}\,dy = \int _\mathcal {O}u \cdot \mathrm{curl}\;(\mathrm{curl}\;{\mathrm {v}})\,dy + {}_{\mathbb {H}^{-1/2}(\varGamma )}\langle \mathbf {n} \times u \big |_\varGamma , \mathrm{curl}\;{\mathrm {v}}\big |_\varGamma \rangle _{\mathbb {H}^{1/2}(\varGamma )}. \end{aligned}$$

To finish the proof we need the following lemma: $\square$

### Lemma 29

Let $u \in \mathbb {H}^1(\mathcal {O})$ such that $u \cdot \mathbf {n} = 0$ on $\varGamma$ and $\varPhi \in \mathbb {H}^1(\mathcal {O})$ with $\mathbf {n}\times \varPhi = 0$ on $\varGamma$. Then

189 $$\begin{aligned} {}_{\mathbb {H}^{-1/2}(\varGamma )}\langle \mathbf {n} \times u \big |_\varGamma , \varPhi \big |_\varGamma \rangle _{\mathbb {H}^{1/2}(\varGamma )} = 0. \end{aligned}$$

### Proof

It is sufficient to prove (189) for $u \in C^1_0(\overline{\mathcal {O}})$ with $u \cdot \mathbf {n} =0$ on $\varGamma$. In this case for all $x \in \varGamma$

$$\begin{aligned} \left( \mathbf {n} \times u\right) \cdot \varPhi = -\left( u \times \mathbf {n}\right) \cdot \varPhi = - u \cdot \left( n \times \varPhi \right) = 0. \end{aligned}$$

$$\square $$

The proof of Lemma 28 is finished by observing that

$$\begin{aligned} \left( u, A {\mathrm {v}}\right) _{\mathbb {L}^2(\mathcal {O})} = \int _\mathcal {O}u \cdot \mathrm{curl}\;(\mathrm{curl}\;{\mathrm {v}})\,dy,\end{aligned}$$

from the proof of Theorem 5. $\square$

Let us consider an abstract framework. Let *H* be a Hilbert space and *A* be a non-negative self-adjoint operator on *H*.

### Lemma 30

Let $u \in D(A^{1/2})$ and ${\mathrm {v}}\in D(A)$. Then

$$\begin{aligned}\left( A^{1/2} u, A^{1/2} {\mathrm {v}}\right) _H = (u, A {\mathrm {v}})_H.\end{aligned}$$

### Proof

Take $u \in D(A^{1/2})$, ${\mathrm {v}}\in D(A)$. Then $A^{1/2} {\mathrm {v}}\in D(A^{1/2})$. So by self-adjointness of $A^{1/2}$ and $A^{1/2}A^{1/2} = A$,

$$\begin{aligned}\left( A^{1/2} u , A^{1/2}{\mathrm {v}}\right) _H = \left( u, A^{1/2}(A^{1/2} {\mathrm {v}})\right) _H = \left( u, A{\mathrm {v}}\right) _H.\end{aligned}$$

$$\square $$

### Lemma 31

Let $u, {\mathrm {v}}\in D(A^{1/2})$, then by Lemmas 30 and 28,

$$\begin{aligned}\left( \mathrm{curl}\;u , \mathrm{curl}\;{\mathrm {v}}\right) _{\mathbb {L}^2(\mathcal {O})} = \left( A^{1/2}u, A^{1/2} {\mathrm {v}}\right) _{\mathbb {L}^2(\mathcal {O})}.\end{aligned}$$

### Proof

Let $$u, {\mathrm {v}}\in D(A)$$, then

$$\begin{aligned} \left( A^{1/2}u, A^{1/2}{\mathrm {v}}\right) _{\mathbb {L}^2(\mathcal {O})} = \left( u, A {\mathrm {v}}\right) _{\mathbb {L}^2(\mathcal {O})} = \left( \mathrm{curl}\;u, \mathrm{curl}\;{\mathrm {v}}\right) _{\mathbb {L}^2(\mathcal {O})}. \end{aligned}$$

By density argument, this is true for all $u, {\mathrm {v}}\in D(A^{1/2})$. $\square$

As a consequence of the above lemma we have

$$\begin{aligned} \Vert \nabla u \Vert ^2_{\mathbb {L}^2(\mathcal {O})} = \Vert \mathrm{curl}\;u\Vert ^2_{\mathbb {L}^2(\mathcal {O})}, \quad u \in D(A^{1/2}). \end{aligned}$$

## Proof of the Poincaré and the Ladyzhenskaya Inequalities

### Proof of Lemma 13

We will establish the Poincaré inequality (54) following the footsteps of Lemma 2.1 [53] with all the details. By density argument, it is enough to prove (54) for smooth functions. Let $\psi \in \mathcal {C}(\overline{Q_\varepsilon })$ be a real continuous function. We write for any $\xi , \eta \in [1, 1 + \varepsilon ]$:

190 $$\begin{aligned} \begin{aligned} \xi ^2 \psi ^2(\xi , \mathbf{x}) + \eta ^2 \psi ^2(\eta , \mathbf{x})&= 2 \xi \eta \psi (\xi , \mathbf{x})\psi (\eta , \mathbf{x}) + \left[ \xi \psi (\xi , \mathbf{x}) - \eta \psi (\eta , \mathbf{x})\right] ^2 \\&= 2 \xi \eta \psi (\xi , \mathbf{x})\psi (\eta , \mathbf{x}) + \left[ \int _\eta ^\xi \frac{\partial \left( r \psi \right) }{\partial r}(r, \mathbf{x}) dr \right] ^2 \end{aligned} \end{aligned}$$

with $\mathbf{x}= \frac{\mathbf{y}}{|\mathbf{y}|} \in {\mathbb {S}^2}$. We fix $\xi$ and integrate w.r.t. $\eta \in [1, 1+\varepsilon ]$ to obtain

191 $$\begin{aligned} \begin{aligned} \varepsilon \xi ^2 \psi ^2(\xi , \mathbf{x}) + \int _1^{1+\varepsilon } \eta ^2 \psi ^2(\eta , \mathbf{x})\,d\eta&= 2 \xi \psi (\xi , \mathbf{x}) \int _1^{1+\varepsilon } \eta \psi (\eta , \mathbf{x})\,d\eta \\&\quad + \int _1^{1+\varepsilon } \left[ \int _\eta ^\xi \frac{\partial \left( r \psi \right) }{\partial r}(r, \mathbf{x}) dr \right] ^2 d\eta . \end{aligned} \end{aligned}$$

With $\psi = u_r$ and $\xi = 1$, observing that $u_r(1, \mathbf{x}) = 0$ (because of the boundary condition $u \cdot \mathbf {n} = 0$ on $\partial Q_\varepsilon$) from (191) we obtain

192 $$\begin{aligned} \int _1^{1+\varepsilon } \eta ^2 u_r^2(\eta , \mathbf{x})\,d\eta = \int _1^{1+\varepsilon } \left[ \int _\eta ^\xi \frac{\partial \left( r u_r \right) }{\partial r}(r, \mathbf{x}) dr \right] ^2 d\eta . \end{aligned}$$

Applying (191) with $\psi = \widehat{N}_\varepsilon u_\lambda$, we get

193 $$\begin{aligned} \begin{aligned}&\varepsilon \xi ^2 \left[ \widehat{N}_\varepsilon u_\lambda (\xi , \mathbf{x})\right] ^2 + \int _1^{1+\varepsilon } \eta ^2 \left[ \widehat{N}_\varepsilon u_\lambda (\eta , \mathbf{x})\right] ^2 d\eta \\&\qquad = 2 \xi \widehat{N}_\varepsilon u_\lambda (\xi , \mathbf{x}) \int _1^{1+\varepsilon } \eta \widehat{N}_\varepsilon u_\lambda (\eta , \mathbf{x})\,d\eta + \int _1^{1+\varepsilon } \left[ \int _\eta ^\xi \frac{\partial \left( r \widehat{N}_\varepsilon u_\lambda \right) }{\partial r}(r, \mathbf{x}) dr \right] ^2 d\eta . \end{aligned} \end{aligned}$$

Observing from Lemma 6 for every $\psi \in L^2(Q_\varepsilon )$

$$\begin{aligned} \int _1^{1+\varepsilon } r \widehat{N}_\varepsilon \psi (r, \mathbf{x})\,dr = 0 \qquad \forall \mathbf{x}\in {\mathbb {S}^2}, \end{aligned}$$

and since the first term on the LHS of (193) is positive we can simplify (193) as follows

194 $$\begin{aligned} \int _1^{1+\varepsilon } \eta ^2 \left[ \widehat{N}_\varepsilon u_\lambda (\eta , \mathbf{x})\right] ^2 d\eta \le \int _1^{1+\varepsilon } \left[ \int _\eta ^\xi \frac{\partial \left( r \widehat{N}_\varepsilon u_\lambda \right) }{\partial r}(r, \mathbf{x}) dr \right] ^2 d\eta . \end{aligned}$$

Similarly for $\psi = \widehat{N}_\varepsilon u_\varphi$, we have

195 $$\begin{aligned} \int _1^{1+\varepsilon } \eta ^2 \left[ \widehat{N}_\varepsilon u_\varphi (\eta , \mathbf{x})\right] ^2 d\eta \le \int _1^{1+\varepsilon } \left[ \int _\eta ^\xi \frac{\partial \left( r \widehat{N}_\varepsilon u_\varphi \right) }{\partial r}(r, \mathbf{x}) dr \right] ^2 d\eta . \end{aligned}$$

Thus, using (192), (194) and (195) for each of the cases $\psi = u_r$, $\psi = \widehat{N}_\varepsilon u_\lambda$ and $\psi = u_\varphi$, we obtain

196 $$\begin{aligned} \int _1^{1+\varepsilon } \eta ^2 \psi ^2(\eta , \mathbf{x})\, d\eta \le \int _1^{1+\varepsilon } \left[ \int _\eta ^\xi \frac{\partial \left( r \psi \right) }{\partial r}(r, \mathbf{x}) dr \right] ^2 d\eta . \end{aligned}$$

Using the Cauchy–Schwarz inequality, we find

197 $$\begin{aligned} \begin{aligned} \int _1^{1+\varepsilon } \eta ^2 \psi ^2(\eta , \mathbf{x})\, d\eta&\le \int _1^{1+\varepsilon }|\xi - \eta |\,d\eta \int _1^{1+\varepsilon } \left| \frac{\partial \left( r \psi \right) }{\partial r}\right| ^2 dr \le \varepsilon ^2 \int _1^{1+\varepsilon } \left| \frac{\partial \left( r \psi \right) }{\partial r}\right| ^2 dr \\&\le 2\varepsilon ^2 \int _1^{1+\varepsilon } \left| \frac{\partial \psi }{\partial r}\right| ^2 r^2 dr + 2 \varepsilon ^2 \int _1^{1+\varepsilon } |\psi |^2 dr \\&\le 2\varepsilon ^2 \int _1^{1+\varepsilon } \left| \frac{\partial \psi }{\partial r}\right| ^2 r^2 dr + 2 \varepsilon ^2 \int _1^{1+\varepsilon } |r \psi |^2 dr, \end{aligned} \end{aligned}$$

the last inequality follows since $r \ge 1$. On rearranging, we obtain

198 $$\begin{aligned} \left( 1 - 2 \varepsilon ^2\right) \int _1^{1+\varepsilon } r^2 \psi ^2(r, \lambda , \varphi )\, dr \le 2\varepsilon ^2 \int _1^{1+\varepsilon } \left| \frac{\partial \psi }{\partial r}\right| ^2 r^2 dr, \end{aligned}$$

which implies for $0 \le \varepsilon < \frac{1}{2}$

199 $$\begin{aligned} \int _1^{1+\varepsilon } r^2 \psi ^2(r, \lambda , \varphi )\, dr \le 4\varepsilon ^2 \int _1^{1+\varepsilon } \left| \frac{\partial \psi }{\partial r}\right| ^2 r^2 dr. \end{aligned}$$

We then integrate w.r.t. $\lambda$ and $\varphi$ to obtain

200 $$\begin{aligned} \int _{Q_\varepsilon } \psi ^2(\mathbf{y})\, d\mathbf{y}\le 4\varepsilon ^2 \int _{Q_\varepsilon } \left| \frac{\partial \psi }{\partial r}\right| ^2 d\mathbf{y}. \end{aligned}$$

Adding (200) for $\psi = u_r$, $\psi = \widehat{N}_\varepsilon u_\lambda$ and $\psi = \widehat{N}_\varepsilon u_\varphi$; using finally

$$\begin{aligned} \int _{Q_\varepsilon }\left| \frac{\partial }{\partial r} \widetilde{N}_\varepsilon u\right| ^2 d\mathbf{y}\le \Vert \nabla \widetilde{N}_\varepsilon u\Vert ^2_{\mathbb {L}^2(Q_\varepsilon )} = \Vert \mathrm{curl}\;\widetilde{N}_\varepsilon u\Vert ^2_{\mathbb {L}^2(Q_\varepsilon )} \qquad \forall \, u \in \mathrm { V}_\varepsilon , \end{aligned}$$

we conclude the proof of the inequality (54). $\square$

### Proof of Lemma 14

We will prove the lemma for smooth vector fields $u \in \mathcal {C}^\infty (Q_\varepsilon )$. By Lemma 2.3 [53] there exists a constant $c_0 > 0$ s.t.

201 $$\begin{aligned} \Vert \widetilde{N}_\varepsilon u\Vert _{\mathbb {L}^6(Q_\varepsilon )} \le c_0 \Vert \nabla \left( \widetilde{N}_\varepsilon u\right) \Vert _{\mathbb {L}^2(Q_\varepsilon )}. \end{aligned}$$

By Lemma 6.1 [17, Eq. 6.11, Pg 359] for vector fields ${\mathrm {v}}\in \mathcal {C}^1(Q_\varepsilon )$ with ${\mathrm {v}}\cdot \mathbf {n} = 0$ on $\partial Q_\varepsilon$, we have a constant $c_2 > 0$ s.t.

202 $$\begin{aligned} \Vert \nabla {\mathrm {v}}\Vert ^2_{\mathbb {L}^2(Q_\varepsilon )} \le 2 \left[ \Vert \mathrm{div}\;{\mathrm {v}}\Vert ^2_{\mathbb {L}^2(Q_\varepsilon )} + \Vert \mathrm{curl}\;{\mathrm {v}}\Vert ^2_{\mathbb {L}^2(Q_\varepsilon )} + c_2\Vert {\mathrm {v}}\Vert ^2_{\mathbb {L}^2(Q_\varepsilon )}\right] . \end{aligned}$$

Also by Poincaré inequality (54) for all $u \in \mathrm { V}_\varepsilon$, we have

203 $$\begin{aligned} \Vert \widetilde{N}_\varepsilon u\Vert ^2_{\mathbb {L}^2(Q_\varepsilon )} \le 4 \varepsilon ^2 \Vert \mathrm{curl}\;\widetilde{N}_\varepsilon u\Vert ^2_{\mathbb {L}^2(Q_\varepsilon )}. \end{aligned}$$

Using (202) with ${\mathrm {v}}= \widetilde{N}_\varepsilon u$ along with the fact that $\mathrm{div}\;{\mathrm {v}}= 0$ if $\mathrm{div}\;u = 0$ and ${\mathrm {v}}\cdot \mathbf {n} = 0$ on $\partial Q_\varepsilon$ if $u \cdot \mathbf {n} = 0$ on $\partial Q_\varepsilon$ and combining it with the Poincaré inequality (203), we obtain

$$\begin{aligned} \Vert \widetilde{N}_\varepsilon u\Vert ^2_{\mathbb {L}^6(Q_\varepsilon )} \le 2c_0^2 \left[ \Vert \widetilde{N}_\varepsilon u\Vert ^2_{\mathrm { V}_\varepsilon } + 4c_2 \varepsilon ^2 \Vert \widetilde{N}_\varepsilon u \Vert ^2_{\mathrm { V}_\varepsilon } \right] . \end{aligned}$$

Therefore, choosing $c_1 = \sqrt{2} c_0 \left( 1 + 4 c_2 \right) ^{1/2}$ we establish (55) for every $u \in \mathcal {C}^1(Q_\varepsilon )$ with $\mathrm{div}\;u = 0$ on $Q_\varepsilon$ and $u \cdot \mathbf {n} = 0$ on $\partial Q_\varepsilon$. We finish the proof using density argument. $\square$

### Proof of Lemma 18

Let *u* be a tangential vector field defined on ${\mathbb {S}^2}$, $u = (0, u_\lambda , u_\varphi )$. Then using the definition of the map  (see (63)), for $Q_\varepsilon \ni \mathbf{y}= r \mathbf{x}$, $r \in (1,1+\varepsilon )$ and $\mathbf{x}\in {\mathbb {S}^2}$, we have

Using the definitions of Laplace ($\varDelta$) for vector fields in spherical coordinates, Laplace–Beltrami ($\nabla ^2$) for scalars, tangential Laplace ($\varvec{{\Delta }}^\prime \,$) for tangential vector fields and Laplace–Beltrami ($\varDelta ^\prime$) for the scalar defined on ${\mathbb {S}^2}$, we have following relations:

where

If $\mathrm{{div}^\prime }u = 0$, then by the definition of $\mathrm{{div}^\prime }$,

$$\begin{aligned} \frac{1}{\sin \lambda } \frac{\partial }{\partial \lambda }(u_\lambda \sin \lambda ) + \frac{1}{\sin \lambda } \frac{\partial u_\varphi }{\partial \varphi } = 0 \end{aligned}$$

which is equivalent to

$$\begin{aligned} \frac{\partial u_\lambda }{\partial \lambda } + u_\lambda \cot {\lambda } + \frac{1}{\sin \lambda } \frac{\partial u_\varphi }{\partial \varphi } = 0. \end{aligned}$$

Hence using all the above relations, we obtain

Since $\varepsilon \in (0,1)$, the inequality (68) holds. $\square$

## Compactness

### Skorohod Theorem and Aldous Condition

Let *E* be a separable Banach space with the norm $\Vert \cdot \Vert _E$ and let $\mathcal {B}(E)$ be its Borel $\sigma$-field. The family of probability measures on $(E, \mathcal {B}(E))$ will be denoted by $\mathcal {P}$. The set of all bounded and continuous *E*-valued functions is denoted by $\mathcal {C}_b(E)$.

#### Definition 3

The family $\mathcal {P}$ of probability measures on $\left( E, \mathcal {B}(E)\right)$ is said to be tight if for arbitrary $\varepsilon > 0$ there exists a compact set $K_\varepsilon \subset E$ such that

$$\begin{aligned} \mu (K_\varepsilon ) \ge 1 - \varepsilon , \quad \text{ for } \text{ all }\,\, \mu \in \mathcal {P}.\end{aligned}$$

We used the following Jakubowski’s generalisation of the Skorokhod Theorem, in the form given by Brzeźniak and Ondreját [14, Theorem C.1], see also [31], as our topological space $\mathcal {Z}_T$ is not a metric space.

#### Theorem 6

Let $\mathcal {X}$ be a topological space such that there exists a sequence $\{f_m\}_{m \in \mathbb {N}}$ of continuous functions $f_m : \mathcal {X} \rightarrow \mathbb {R}$ that separates points of $\mathcal {X}$. Let us denote by $\mathcal {S}$ the $\sigma$-algebra generated by the maps $\{f_m\}$. Then

- every compact subset of $\mathcal {X}$ is metrizable,
- if $(\mu _m)_{m \in \mathbb {N}}$ is a tight sequence of probability measures on $(\mathcal {X}, \mathcal {S})$, then there exists a subsequence $(m_k)_{k \in \mathbb {N}}$, a probability space $(\varOmega , \mathcal {F}, \mathbb {P})$ with $\mathcal {X}$-valued Borel measurable variables $\xi _k, \xi$ such that $\mu _{m_k}$ is the law of $\xi _k$ and $\xi _k$ converges to $\xi$ almost surely on $\varOmega$.

Let $(\mathbb {S}, \varrho )$ be a separable and complete metric space.

#### Definition 4

Let $u \in \mathcal {C}([0,T]; \mathbb {S})$. The modulus of continuity of *u* on [0, *T*] is defined by

$$\begin{aligned} m(u, \delta ) := \sup _{s,t \in [0,T],\,|t - s|\le \delta } \varrho (u(t), u(s)), \quad \delta > 0. \end{aligned}$$

Let $(\varOmega , \mathcal {F}, \mathbb {P})$ be a probability space with filtration $\mathbb {F}:= (\mathcal {F}_t)_{t \in [0,T]}$ satisfying the usual conditions, see [38], and let $(X_n)_{n \in \mathbb {N}}$ be a sequence of continuous $\mathbb {F}$-adapted $\mathbb {S}$-valued processes.

#### Definition 5

We say that the sequence $(X_n)_{n \in \mathbb {N}}$ of $\mathbb {S}$-valued random variables satisfies condition $[\mathbf {T}]$ iff $\forall \, \varepsilon>0, \forall \, \eta> 0,\, \exists \, \delta > 0$:

204 $$\begin{aligned} \sup _{n \in \mathbb {N}} \mathbb {P}\left\{ m(X_n, \delta ) > \eta \right\} \le \varepsilon . \end{aligned}$$

#### Lemma 32

[12, Lemma 2.4] Assume that $(X_n)_{n \in \mathbb {N}}$ satisfies condition $[\mathbf {T}]$. Let $\mathbb {P}_n$ be the law of $X_n$ on $\mathcal {C}([0,T]; \mathbb {S})$, $n \in \mathbb {N}$. Then for every $\varepsilon > 0$ there exists a subset $A_\varepsilon \subset \mathcal {C}([0,T]; \mathbb {S})$ such that

$$\begin{aligned} \sup _{n \in \mathbb {N}} \mathbb {P}_n(A_\varepsilon ) \ge 1 - \varepsilon \end{aligned}$$

and

205 $$\begin{aligned} \lim _{\delta \rightarrow 0} \sup _{u \in A_\varepsilon } m(u, \delta ) = 0. \end{aligned}$$

Now we recall the Aldous condition $[\mathbf {A}]$, which is connected with condition $[\mathbf {T}]$ (see [39] and [1]). This condition allows to investigate the modulus of continuity for the sequence of stochastic processes by means of stopped processes.

#### Definition 6

(*Aldous condition*) A sequence $(X_n)_{n \in \mathbb {N}}$ satisfies condition $[\mathbf {A}]$ iff $\,\forall \, \varepsilon > 0$, $\forall \, \eta > 0$, $\exists \, \delta > 0$ such that for every sequence $(\tau _n)_{n \in \mathbb {N}}$ of $\mathbb {F}$-stopping times with $\tau _n \le T$ one has

$$\begin{aligned} \sup _{n \in \mathbb {N}} \sup _{0 \le \theta \le \delta } \mathbb {P}\left\{ \varrho (X_n(\tau _n + \theta ), X_n(\tau _n)) \ge \eta \right\} \le \varepsilon . \end{aligned}$$

#### Lemma 33

[39, Theorem 3.2] Conditions $[\mathbf {A}]$ and $[\mathbf {T}]$ are equivalent.

### Tightness Criterion

Now we formulate the compactness criterion analogous to the result due to Mikulevicus and Rozowskii [40], Brzeźniak and Motyl [12] for the space $\mathcal {Z}_T$, see also [5, Lemma 4.2].

#### Lemma 34

Let $\mathcal {Z}_T$, $\mathcal {T}$ be as defined in (116). Then a set $\mathcal {K}\subset \mathcal {Z}_T$ is $\mathcal {T}$-relatively compact if the following three conditions hold

- $$\sup _{u \in \mathcal {K}} \sup _{s \in [0,T]} \Vert u(s)\Vert _{{\mathbb {L}^2({\mathbb {S}^2})}} < \infty ,$$
- $\sup _{u \in \mathcal {K}} \int _0^T \Vert u(s)\Vert ^2_\mathrm { V}\,ds < \infty \,$, i.e. $\mathcal {K}$ is bounded in $L^2(0,T; \mathrm { V})$,
- $$\lim _{\delta \rightarrow 0} \sup _{u \in \mathcal {K}} \sup _{\underset{|t-s| \le \delta }{s,t \in [0,T]}}\Vert u(t) - u(s)\Vert _{\mathrm {D}(\mathrm { A}^{-1})} = 0.$$

Using Sect. D.1 and the compactness criterion from Lemma 34 we obtain the following corollary.

#### Corollary 6

(Tightness criterion) Let $(\alpha _\varepsilon )_{\varepsilon > 0}$ be a sequence of continuous $\mathbb {F}$-adapted $\mathrm {H}$-valued processes such that

- there exists a constant $C_1 > 0$ such that
  $$\begin{aligned}\sup _{\varepsilon > 0}\,\mathbb {E}\left[ \sup _{s \in [0,T]} \Vert {\alpha }_\varepsilon (s)\Vert ^2_{\mathrm {H}} \right] \le C_1,\end{aligned}$$
- there exists a constant $C_2 > 0$ such that
  $$\begin{aligned}\sup _{\varepsilon > 0}\,\mathbb {E}\left[ \int _0^T \Vert \mathrm{{curl}^\prime }{\alpha }_\varepsilon (s)\Vert ^2_{{\mathbb {L}^2({\mathbb {S}^2})}}\,ds \right] \le C_2,\end{aligned}$$
- $({\alpha }_\varepsilon )_{\varepsilon > 0}$ satisfies the Aldous condition $[\mathbf {A}]$ in $\mathrm {D}(\mathrm { A}^{-1})$.

Let $\widetilde{\mathbb {P}}_\varepsilon$ be the law of ${\alpha }_\varepsilon$ on $\mathcal {Z}_T$. Then for every $\delta > 0$ there exists a compact subset $K_\delta$ of $\mathcal {Z}_T$ such that

$$\begin{aligned} \sup _{\varepsilon > 0} \widetilde{\mathbb {P}}_\varepsilon (K_\delta ) \ge 1 - \delta . \end{aligned}$$

## Kuratowski Theorem and Proof of Lemma 25

This appendix is dedicated to the proof of Lemma 25. We will first recall the Kuratowski Theorem [33] in the next subsection and prove some related results which will be used later to prove Lemma 25 in Sect. E.2.

### Kuratowski Theorem and Related Results

#### Theorem 7

Assume that $X_1, X_2$ are the Polish spaces with their Borel $\sigma$-fields denoted respectively by $\mathcal {B}(X_1), \mathcal {B}(X_2)$. If $\varphi :X_1 \rightarrow X_2$ is an injective Borel measurable map then for any $E_1 \in \mathcal {B}(X_1)$, $E_2 := \varphi (E_1) \in \mathcal {B}(X_2)$.

Next two lemmas are the main results of this appendix. For the proof of Lemma 35 please see [6, Appendix B].

#### Lemma 35

Let $X_1, X_2$ and *Z* be topological spaces such that $X_1$ is a Borel subset of $X_2$. Then $X_1 \cap Z$ is a Borel subset of $X_2 \cap Z$, where $X_2 \cap Z$ is a topological space too, with the topology given by

206 $$\begin{aligned} \tau (X_2 \cap Z) = \left\{ A \cap B : A \in \tau (X_2), B \in \tau (Z)\right\} . \end{aligned}$$

### Proof of Lemma 25

In this subsection we recall Lemma 25 and prove it using the results from previous subsection.

#### Lemma 36

Let $T > 0$ and $\mathcal {Z}_T$ be as defined in (116). Then, the following sets $\mathcal {C}([0,T];\mathrm { H}) \cap \mathcal {Z}_T$, $L^2(0,T; \mathrm { V}) \cap \mathcal {Z}_T$ are Borel subsets of $\mathcal {Z}_T$.

#### Proof

First of all $\mathcal {C}([0,T]; \mathrm { H}) \subset \mathcal {C}([0,T]; \mathrm {D}(\mathrm { A}^{-1})) \cap L^2(0,T; \mathrm { H})$. Secondly, $\mathcal {C}([0,T]; \mathrm { H})$ and $\mathcal {C}([0,T]; \mathrm {D}(\mathrm { A}^{-1})) \cap L^2(0,T; \mathrm { H})$ are Polish spaces. And finally, since $\mathrm { H}$ is continuously embedded in $\mathrm {D}(\mathrm { A}^{-1})$, the map

$$\begin{aligned} i :\mathcal {C}([0,T]; \mathrm { H}) \rightarrow \mathcal {C}([0,T]; \mathrm {D}(\mathrm { A}^{-1})) \cap L^2(0,T; \mathrm { H}),\end{aligned}$$

is continuous and hence Borel. Thus, by application of the Kuratowski Theorem (see Theorem 7), $\mathcal {C}([0,T]; \mathrm { H})$ is a Borel subset of $\mathcal {C}([0,T]; \mathrm {D}(\mathrm { A}^{-1})) \cap L^2(0,T; \mathrm { H})$. Therefore, by Lemma 35, $\mathcal {C}([0,T]; \mathrm { H}) \cap {\mathcal {Z}}_T$ is a Borel subset of $\mathcal {C}([0,T]; \mathrm {D}(\mathrm { A}^{-1})) \cap L^2(0,T; \mathrm { H}) \cap {\mathcal {Z}}_T$ which is equal to ${\mathcal {Z}}_T$.

Similarly we can show that $L^2(0,T; \mathrm { V}) \cap {\mathcal {Z}}_T$ is a Borel subset of ${\mathcal {Z}}_T$. $L^2(0,T; \mathrm { V}) \hookrightarrow L^2(0,T; \mathrm { H})$ and both are Polish spaces thus by application of the Kuratowski Theorem, $L^2(0,T; \mathrm { V})$ is a Borel subset of $L^2(0,T; \mathrm { H})$. Finally, we can conclude the proof of lemma by Lemma 35. $\square$

## Footnotes

1 We could have considered the case $N = \infty$. This case will be considered in the companion paper [7].

2 The space $X^w$ denotes a topological space *X* with weak topology. In particular, $\mathcal {C}([0,T]; X^w)$ is the space of weakly continuous functions ${\mathrm {v}}:[0,T] \rightarrow X$.

3 $\mathcal {T}$ is the supremum of topologies $\mathbf {T}_1$, $\mathbf {T}_2$, $\mathbf {T}_3$ and $\mathbf {T}_4$, i.e. it is the coarsest topology on $\mathcal {Z}_T$ that is finer than each of $\mathbf {T}_1$, $\mathbf {T}_2$, $\mathbf {T}_3$ and $\mathbf {T}_4$.

4 The space $\mathcal {Z}_T \times \mathcal {Y}_T \times \mathcal {C}([0,T]; \mathbb {R}^N)$ satisfies the assumption of Theorem 6. Indeed, since $Z_T$ and $\mathcal {Y}_T^\varepsilon$, $\varepsilon > 0$ satisfies the assumptions (see [5, Lemma 4.10]) and $\mathcal {C}([0,T]; \mathbb {R}^N)$ is a Polish space and thus automatically satisfying the required assumptions.
